# Sorbicillinoid Derivatives From Sponge-Derived Fungus *Trichoderma reesei* (HN-2016-018)

**DOI:** 10.3389/fmicb.2020.01334

**Published:** 2020-06-23

**Authors:** Saif Ur Rehman, Lu-Jia Yang, Ya-Hui Zhang, Jing-Shuai Wu, Ting Shi, Waqas Haider, Chang-Lun Shao, Chang-Yun Wang

**Affiliations:** ^1^Key Laboratory of Marine Drugs, The Ministry of Education of China, School of Medicine and Pharmacy, Ocean University of China, Qingdao, China; ^2^Institute of Evolution & Marine Biodiversity, Ocean University of China, Qingdao, China; ^3^Laboratory for Marine Drugs and Bioproducts, Qingdao National Laboratory for Marine Science and Technology, Qingdao, China; ^4^Department of Pharmacy, Faculty of Medical and Health Sciences, University of Poonch Rawalakot, Rawalakot, Pakistan

**Keywords:** sponge-derived fungus, *Trichoderma reesei*, sorbicillinoid, naphthalene-trione, cytotoxic activity

## Abstract

Six new sorbicillinoids, trichoreeseione A (**1**) and B (**2**), trichodermolide B (**3**), 13-hydroxy**-**trichodermolide (**4**), 24-hydroxy-trichodimerol (**5**), 15-hydroxy**-**bisvertinol (**7**), together with three known analogs, trichodimerol (**6**), 24-hydroxy**-**bisvertinol (**8**), and bisvertinol (**9**), were isolated from the sponge-derived fungus *Trichoderma reesei* (HN-2016-018). Their structures including absolute configurations were elucidated by analysis of NMR, MS data, and calculated ECD spectra. Compounds **1** and **2** with a characteristic naphthalene-trione ring were firstly reported in sorbicillinoid family. Compounds **3** and **4** were two rare sorbicillinoids containing a unique bicycle [3.2.1] lactone skeleton, while **3** with a propan-2-one moiety was also recorded first time in this family. Compound **5** displayed cytotoxic activity against A549, MCF-7, and HCT116 cell lines with the IC_50_ values of 5.1, 9.5, and 13.7 μM, respectively.

## Introduction

Sorbicillionids are a family of hexaketide metabolites with a characteristic sorbyl side chain residue (Harned and Volp, 2011), which was firstly isolated as an impurity in penicillin in 1948 (Andrade et al., 1992). Since then more than 100 analogs of sorbicillinoids have been isolated, which could be classified into monomeric, dimeric, trimeric, and polycyclic sorbicillinoids, and vertinolides according to their basic structural features (Harned and Volp, 2011). Sorbicillinoid family possesses extensive pharmacological effects including cytotoxic (Du et al., 2009; Ei-Elimat et al., 2015), antimicrobial (Ei-Elimat et al., 2015), antiviral (Peng et al., 2014), anti-inflammatory (Zhang et al., 2019), radical scavenging (Abe et al., 1998), and anti-diabetic activities (Cao et al., 2019). Majority of sorbicillionids were reported from 10 genera of fungi: *Penicillium*, *Phaeoacremonium*, *Trichoderma*, *Aspergillus*, *Phialocephala*, *Scytalidium*, *Clonostachys*, *Acremonium*, *Paecilomyces*, and *Verticillium* (Meng et al., 2016). In recent years, marine-derived fungi have emerged as an important resource for sorbicillinoids. Diverse sorbicillinoid analogs with unique skeletons have been reported from sponge associated fungi. For example, a group of sorbicillionids, saturnispols A–H possessing excellent antibacterial activity, especially against gram negative bacteria, were isolated from sponge-derived fungus *Trichoderma saturnisporum* DI-IA (Meng et al., 2018), while a series of dimeric and monomeric sorbicillinoid derivatives with potent anti-inflammatory activity were reported from sponge-associated fungus *Trichoderma reesei* 4670 (Zhang et al., 2019).

**FIGURE 1:**
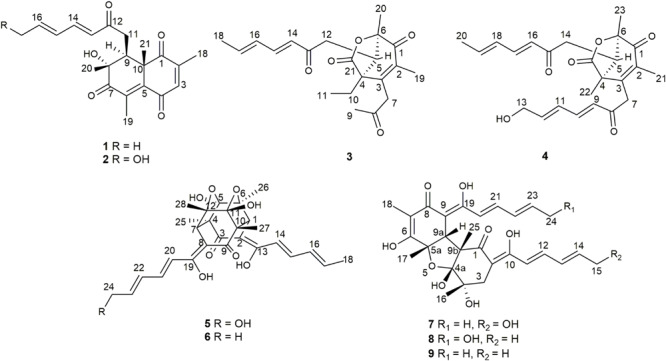
Structures of isolated compounds **1**–**9**.

During our recent research for exploration of new structurally diverse bioactive natural products from marine invertebrates and their symbiotic microorganisms (Liu et al., 2019), a variety of new secondary metabolites with multiple biological activities have been obtained from sponge-derived fungi (Li et al., 2012; Qi et al., 2013; Zhao et al., 2015; Wang et al., 2018). In this study, the strain *T. reesei* (HN-2016-018) isolated from an unidentified sponge collected from the South China Sea attracted our attention because the EtOAc extract from its fermentation broth exhibited cytotoxic activity. Chemical investigation led to the isolation of nine sorbicillionoid derivatives (Figure 1), including six new sorbicillionoids, trichoreeseione A (**1**), trichoreeseione B (**2**), trichodermolide B (**3**), 13-hydroxy-trichodermolide (**4**), 24-hydroxy-trichodimerol (**5**), and 24-hydroxy-bisvertinol (**7**), along with three known analogs, trichodimerol (**6**) (Andrade et al., 1992), 15 hydroxy-bisvertinol (**8**) (Zhang et al., 2019), and bisvertinol (**9**) (Trifonov et al., 1986). Herein, we report the isolation, structural elucidation, and cytotoxic activities of these compounds.

## Materials and Methods

### General Experimental Procedure

The optical rotations were measured on a JASCO P-1020 digital polarimeter. IR spectra were recorded on a Nicolet-Nexus-470 spectrometer using KBr pellets (Thermo Electron, Waltham, MA, United States). UV spectra were recorded using a Milton Roy UV-Vis spectrophotometer. NMR spectra were acquired using a JEOL JEMECP NMR spectrometer (JEOL, Tokyo, Japan) (600 MHz for ^1^H, 150 MHz for ^13^C) and BRUKER AVANCE NEO NMR spectrometer (BRUKER, United States) (400 MHz for ^1^H, and 100 MHz for ^13^C), using TMS as an internal standard. ECD spectra were recorded on a JASCO J-815 circular dichroism spectrometer (JASCO Electric Co., Ltd., Tokyo, Japan). ESIMS spectra were measured on a Micromass Q-TOF spectrometer (Waters Corp., Manchester, United Kingdom). HPLC separation was performed using a Hitachi L-2000 HPLC system (Hitachi High Technologies, Tokyo, Japan) coupled with a Hitachi L-2455 photodiode array detector. A Kromasil C_18_ semi-preparative HPLC column (250 × 10 mm, 5 μm) (Eka Nobel, Bohus, Sweden) was used. Silica gel (Qingdao Marine Chemical Group Co., Qingdao, China) and Sephadex LH-20 (Amersham Biosciences Inc., Piscataway, NJ, United States) were used for column chromatography. Precoated silica gel GF254 plates (Yantai Zifu Chemical Group Co., Yantai, China) were used for analytical TLC.

### Fungal Material

The fungal strain *T. reesei* (HN-2016-018) was separated from the internal fresh tissue of an unidentified sponge collected from the South China Sea in October 2016. The fungal strain was recognized on the basis of its morphological traits and by amplification and sequencing of the DNA sequences of the ITS region. The fungus was identified as *T. reesei* whose 500 base pair ITS sequence had 99% sequence identity to that of *T. reesei.* The phylogenetic tree (Supplementary Figure S50) was constructed using the neighbor joining method (Saitou and Nei, 1987). The distance calculations, tree construction, and bootstrap analysis were performed with the software MEGA 7 (Felsenstein, 1985). The strain was deposited in the Key Laboratory of Marine Drugs, the Ministry of Education of China, School of Medicine and Pharmacy, Ocean University of China, Qingdao, China, with the Gene Bank (NCBI) accession number MT367415.

### Fermentation, Extraction, and Isolation

The fungal strain *T. reesei* (HN-2016-018) was cultivated in 80 L PDB medium at r.t. for 1 month. Broth and mycelia in fermented culture were separated by filtration. The filtrate was extracted three times with equal volume of EtOAc, and the mycelia were soaked in EtOAc (3 × 5000 mL). The TLC profiles of these two parts were similar, so these extracts were combined and obtained the EtOAc extract (103 g). The extract was subjected to VLC on silica gel (100–200 mesh) eluting with a combination solvent of PE/EtOAc in increasing polarity (90:10, 50:50, 30:70, 0:100) followed by CH_2_Cl_2_/MeOH (from 90:10 to 30:70) to obtain six fractions (Fr.1–Fr.6). Fr.3 was subjected to silica gel column chromatography (CC) to obtain two sub-fractions (Fr.3-1 and Fr.3-2). Fr.3-1 was chromatographed over a Sephadex LH-20 column using MeOH as mobile phase and afterward isolated by semi-preparative HPLC (75% MeOH/H_2_O) to yield **1** (7.0 mg) and **2** (5.5 mg). Fr.3-2 was separated by HPLC (70% CH_3_CN/H_2_O) to give **4** (7 mg). Fr. 4 was fractionated with Sephadex LH-20 column using PE–CH_2_Cl_2_–MeOH (v/v, 2:1:1) as mobile phase to achieve four sub-fractions (Fr.4-1 to Fr.4-4). Pure compound **9** (10 mg) was obtained from Fr.4-1 by CC on Sephadex LH-20 with mobile phase CH_2_Cl_2_–MeOH (v/v, 1:1). Fr.4-2 was eluted with PE–EtOAc (v/v, 70:30) on silica gel CC and then purified with RP-HPLC (70% MeOH/H_2_O) to afford **3** (6 mg). Fr.4-3 was processed with Sephadex LH-20 column and then further fractioned by RP-HPLC (65% MeOH/H_2_O) to give **5** (8 mg) and **6** (25 mg). Fr.4-4 was subjected to RP-HPLC (45% MeOH/H_2_O) to afford **7** (4.5 mg) and **8** (12 mg).

Trichoreeseione A (**1**) yellow oil; [α]25 D − 42.7 (*c* 0.1, MeOH); UV (MeOH) λ_max_ (log ε) 324 (4.784) nm; ECD (*c* 2.8 mM, MeOH) λmax (Δε) 227 (−8.9), 252 (+ 25.1), 288 (−20.8) nm; IR (KBr) *v*_max_ 3614, 3566, 3525, 1700, 1683, 1509, 1398, 1026 cm^–1^; ^1^H and ^13^C NMR see Table 1; HR-ESIMS *m*/*z* 357.1692 [M + H]^+^ (calcd for C_21_H_25_O_5_, 357.1697), 379.1511 [M + Na] ^+^ (calcd for C_21_H_24_O_5_Na, 379.1516).

**TABLE 1:** ^1^H (600 MHz) and ^13^C NMR (150 MHz) spectroscopic data for compounds 1–2.

**No.**	**1 in CDCl_3_**		**2 in CDCl_3_**	
	**δ_C_ type**	**δ_H_, (*J* in Hz)**	**δ_*C*,_ type**	**δ_H_, (*J* in Hz)**
1	199.1, C		199.0, C	
2	148.8, C		148.7, C	
3	137.7, CH	6.75, s	137.7, CH	6.76, s
4	190.5, C		190.3, C	
5	147.6, C		147.6, C	
6	133.6, C		133.5, C	
7	202.5, C		202.3, C	
8	74.9, C		74.8, C	
9	41.9, CH	3.45, dd (7.5, 3.7)	42.0, CH	3.44, dd (7.7, 3.8)
10	54.4, C		54.3, C	
11	38.4, CH_2_	2.94, dd (15.5, 7.5)	38.6, CH_2_	2.96, dd (15.5, 7.7)
		2.84, dd (15.5, 3.7)		2.86, dd (15.5, 3.8)
12	198.8, C		198.5, C	
13	127.3, CH	6.27, d (15.5)	128.6, CH	6.38, d (15.5)
14	143.0, CH	7.30, dd (15.5, 9.7)	141.4, CH	7.34, dd (15.5, 10.9)
15	130.5, CH	6.21, m	129.4, CH	6.45, m
16	140.3, CH	6.20, m	141.6, CH	6.29, dt (15.5, 4.2)
17	18.9, CH_3_	1.87, d (5.1)	62.9, CH_2_	4.31, d (4.2)
18	17.0, CH_3_	2.03, s	16.9, CH_3_	2.04, s
19	12.9, CH_3_	2.01, s	12.8, CH_3_	2.02, s
20	22.4, CH_3_	1.34, s	22.4, CH_3_	1.34, s
21	21.5, CH_3_	1.57, s	21.4, CH_3_	1.57, s
8-OH		3.55, brs		3.76, brs

Trichoreeseione B (**2**) yellow oil; [α]25 D − 39.9 (*c* 0.1, MeOH); UV (MeOH) λ_max_ (log ε) 307 (4.236) nm; ECD (*c* 2.7 mM, MeOH) λmax (Δε) 226 (−6.8), 251 (+ 20.2), 286 (−18.2) nm; IR (KBr) *v*_max_ 3629, 3567, 1700, 1683, 1650, 1521, 1457 cm^–1^; ^1^H and ^13^C NMR see Table 1; HR-ESIMS *m*/*z* 373.1642 [M + H] ^+^ (calcd for C_21_H_25_O_6_, 373.1646).

Trichodermolide B (**3**) yellow oil; [α]25 D + 64.2 (*c* 0.1, MeOH); UV (MeOH) λ_max_ (log ε) 308 (4.65) nm; ECD (*c* 1.40 mM, MeOH) λmax (Δε) 221 (−27.4), 267 (+27.1) nm; IR (KBr) *v*_max_ 3023, 2934, 1785, 1721, 1683, 1634, 1597, 1436, 1381, 1186, 979 cm^–1^; ^1^H and ^13^C NMR see Table 2; HR-ESIMS *m*/*z* 359.1861 [M + H]^+^ (calcd for C_21_H_27_O_5_, 359.1853), 381.1672 [M + Na]^+^ (calcd for C_21_H_26_O_5_Na, 381.1672).

**TABLE 2:** ^1^H (600 MHz) and ^13^C NMR (150 MHz) for compounds 3–4.

**No.**	**3 in DMSO-*d*_6_**		**4 in CDCl_3_**	
	**δ_C_, type**	**δ_H_ (*J* in Hz)**	**δ_C_, type**	**δ_H_ (*J* in Hz)**
1	191.2, C		191.2, C	
2	133.9, C		134.3, C	
3	150.9, C		149.3, C	
4	55.9, C		51.7, C	
5	50.0, CH	3.37, m	55.9, CH	3.33, dd (6.4, 4.3)
6	86.5, C		87.2, C	
7	44.7, CH_2_	3.73, s	42.1, CH_2_	3.67, d (5.4)
8	205.0, C		195.4, C	
9	30.5, CH_3_	2.20, s	128.3, CH	6.20, d (15.5)
10	20.5, CH_2_	1.25, dd (14.1, 7.2)	143.9, CH	7.24, dd (15.5, 10.9)
		2.03, dd (14.1,7.2)		
11	8.6, CH_3_	0.82, t (7.2)	127.5, CH	6.46, m
12	35.0, CH_2_	2.54, dd (18.1, 6.2)	143.6, CH	6.35, dt (15.5, 4.6)
		2.99, dd (18.1, 6.2)		
13	197.2		62.6, CH_2_	4.34, d (4.6)
14	127.6, CH	6.14, d (15.5)	35.1, CH_2_	3.23, dd (18.4, 6.4)
				2.46, dd (18.4, 4.3)
15	143.9, CH	7.17, dd (15.5, 10.1)	196.6, C	
16	130.6, CH	6.27, m	126.8, CH	6.11, d (15.5)
17	141.9, CH	6.32, m	144.2, CH	7.20, dd (15.5, 10.7)
18	19.1, CH_3_	1.83, d (6.1)	130.2, CH	6.20, m
19	11.9, CH_3_	1.70, s	141.7, CH	6.28, m
20	16.5, CH_3_	1.37, s	18.9, CH_3_	1.88, d (6.6)
21	174.7, C		11.6, CH_3_	1.78, s
22			16.1, CH_3_	1.23, s
23			16.2, CH_3_	1.46, s
24			176.1, C	

13-Hydroxy-trichodermolide (**4**) yellow amorphous powder; [α]25 D + 27.6 (*c* 0.1, MeOH); UV (MeOH) λ_max_ (log ε) 272 (4.18) nm; IR (KBr) *v*_max_ 3023, 2934, 1785, 1721, 1683, 1634, 1597, 1436, 1381, 1186, 979 cm^–1^; ECD (*c* 1.12 mM, MeOH) λmax (Δε) 222 (−9.9), 266 (+7.8) nm; ^1^H and ^13^C NMR see Table 2; HR-ESIMS *m*/*z* 413.1956 [M + H]^+^ (calcd for C_24_H_29_O_6_, 413.1959), 435.1773 [M + Na]^+^ (calcd for C_24_H_28_O_6_Na, 435.1778).

24-Hydroxy-trichodimerol (**5**) yellow amorphous powder; [α]25 D − 405.6 (*c* 0.1, MeOH); UV (MeOH) λ_max_ (log ε) 358 (4.332) nm; ECD (*c* 1.07 mM, MeOH) λmax (Δε) 338 (+56.1), 383 (−83.5) nm; IR (KBr) *v*_max_ 3435, 2979, 2933, 1615, 1297, 1178, 1121 cm^–1^; ^1^H and ^13^C NMR see Table 3; HR-ESIMS *m*/*z* 513.2129 [M + H]^+^ (calcd for C_28_H_33_O_9_, 513.2130).

**TABLE 3:** ^1^H and ^13^C NMR spectroscopic data for compounds 5 and 7.

**No.**	**5^a^ in CD_3_OD**		**7^b^ in CD_3_OD**	
	**δ_*C*,_ type**	**δ_H_, (*J* in Hz)**	**δ_*C*,_ type**	**δ_H_, (*J* in Hz)**
1	58.7, CH	3.08, s	195.3, C	
2	104.6^c^, C		106.5, C	
3	201.3, C		36.5, CH_2_	2.46, d (14.5)
				2.74, d (14.5)
4	61.0^d^, C		74.3, C	
4a			107.3, C	
5	105.8, C			
5a			80.6, C	
6	80.4, C		169.1, C	
7	58.7, CH	3.09, s	110.7, C	
8	105.0^c^, C		193.5, C	
9	201.9, C		102.6, C	
9a			55.1, CH	3.65, s
9b			60.6, C	
10	61.1^d^, C		178.7, C	
11	105.8, C		123.6, CH	6.42, d (14.7)
12	80.4, C		142.3, CH	7.27, dd (14.7, 11.2)
13	175.9, C		129.7, CH	6.54, m
14	120.2, CH	6.31, d (14.7)	143.2, CH	6.26, dd (14.7, 4.3)
15	144.2, CH	7.28, dd (14.7, 11.1)	62.9, CH_2_	4.22, d (4.3)
16	132.3, CH	6.37, m	22.8, CH_3_	1.21, s
17	140.8, CH	6.21, dd (14.7, 6.6)	25.8, CH_3_	1.43, s
18	18.9, CH_3_	1.89, d (6.6)	7.1, CH_3_	1.41, s
19	175.2, C		168.6, C	
20	122.1, CH	6.43, d (14.7)	121.8, CH	6.49, d (14.7)
21	143.1, CH	7.33, dd (14.7, 11.3)	139.4, CH	7.19, dd (14.7, 11.2)
22	129.7, CH	6.56, dd (14.7, 11.3)	132.5, CH	6.35, m
23	143.4, CH	6.27, dt (14.7, 4.4)	137.3, CH	6.11, dq (14.7, 6.6)
24	62.9, CH_2_	4.22, d (4.4)	18.7, CH_3_	1.87, d (6.6)
25	19.8, CH_3_	1.37, s	20.1, CH_3_	1.28, s
26	21.8, CH_3_	1.35, s		
27	19.8, CH_3_	1.37, s		
28	21.8, CH_3_	1.35, s		

*^*a*^Recorded at 600 MHz for ^1^H NMR and at 150 MHz for ^13^C NMR. ^*b*^Recorded at 400 MHz for ^1^H NMR and at 100 MHz for ^13^C NMR. ^*c,d*^Assignment of signals might be exchanged.*

15-Hydroxy-bisvertinol (**7**) yellow powder; [α]25 D − 377 (*c* 0.1, MeOH); UV (MeOH) λ_max_ (log ε) 348 nm (3.874); ECD (*c* 1.95 mM, MeOH) λmax (Δε) 346 (+44.6), 403 (−71.4) nm; IR (KBr) *v*_max_ 3739, 3390, 3265, 2933, 1711, 1612, 1556, 1361, 1027 cm^–1^; ^1^H and ^13^C NMR see Table 3; HR-ESIMS *m*/*z* 513.2136 [M − H]^–^ (calcd for C_28_H_33_O_9_, 513.2130).

### ECD Computational Methods

The MMFF94S force field was used for conformational searches of (8*R*,9*S*,10*S*)-**1**, (8*S*,9*R*,10*R*)-**1**, and (4*S*,5*R*,6*S*)-**3**, respectively. All conformers [20 for (8*R*,9*S*,10*S*)-**1**, 32 for (8*S*,9*R*,10*R*)-**1** and 19 for (4*S*,5*R*,6*R*)-**3**] were optimized at the B3LYP/6-31G(d) level using the Gaussian 09 and then further optimized at the B3LYP/6-311 + G(d) level. The ECD spectrum was calculated by the TDDFT method with the basis set at B3LYP/6-311++G(2d,p) level and simulated by Boltzmann distributions in SpecDis 1.62 (Bruhn et al., 2013).

### Biological Assay

Human tumor cells, including colonic (HCT116 and SW480), lung carcinoma (A-549), hepatocellular carcinoma (HepG2), cervical carcinoma (HeLa), breast cancer (MCF-7), and human normal cells, including human umbilical vein endothelial cells (HUVEC) and hepatocytes CLiver cells were cultured in RPMI 1640 medium supplemented with 10% heat inactivated FBS (fetal bovine serum), 2 mM *L*-glutamine and combination of antibiotics penicillin 100 units/ml and streptomycin 100 g/ml were used to avoid contamination in culture medium. All samples were dissolved in DMSO. The adriamycin was used as a positive control and DMSO was used as a negative control (Wu et al., 2020).

The cytotoxicity of the isolated compounds was determined by sulphorhodamine B assay (Skehan et al., 1990). Cells in logarithmic growth stage were inoculated into 96-well tissue culture plates with 5000 cells/well (180 μL/well) for 24 h before treatment with the tested compounds to allow attachment of the cells to the plate. Cells were exposed to the six different concentrations (1.25, 2.5, 5, 10, 20, and 40 μM) in four parallel. After 72 h of drug action, cold trichloroacetic acid (TCA, 50% w/v) was added into each well to fix the cell. After several washings, cells were stained by 0.4% (w/v) SRB solution for 10 min in dark place. Excess stain was washed with 1% (v/v) glacial acetic acid. After drying overnight, the SRB-stained cells were dissolved with 150 μL/pore Tris solution and the color intensity was measured in microplate reader at 540 nm. The IC_50_ values were analyzed using Graph Pad Prism 5 (GraphPad Software, Inc., La Jolla, CA, United States). The biological assay was carried out under proper aseptic environment to prevent contamination from bacteria, fungi, mycoplasma, and cross contaminations with other cell lines.

### Statistical Analysis

The bioassay results were expressed as mean values ± SD. The IC_50_ values, i.e., the concentrations necessary for 50% inhibition, were calculated from the dose response curves using non-linear regression.

## Results

### Structure Elucidation

**FIGURE 2:**
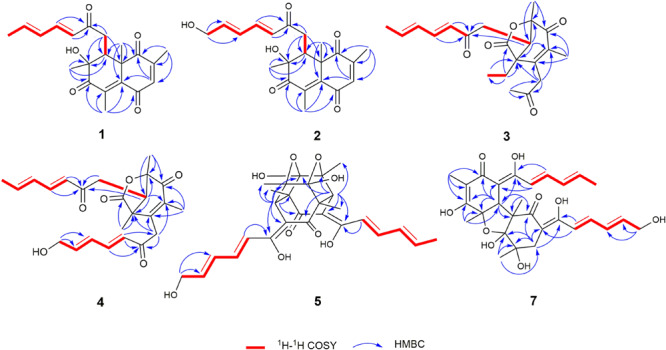
Key ^1^H–^1^H COSY and HMBC correlations of **1**–**5** and **7**.

Trichoreeseione A (**1**) was isolated as a yellow oil with a molecular formula C_21_H_24_O_5_, based on its HR-ESIMS *m*/*z* 357.1692 [M + H]^+^ (calcd for C_21_H_25_O_5_, 357.1697 [M + H]^+^), possessing 10 degrees of unsaturation. The ^1^H NMR spectrum of **1** presented five olefinic protons, one methine, one methylene, five methyls, and one hydroxyl proton (δ_*H*_ 3.55, brs) (Table 1). The ^13^C NMR spectrum exhibited the presence of 21 carbon signals, including eight olefinic carbons, four carbonyl groups and nine sp^3^ hybridized carbons (Table 1), which indicated that two rings should be present in **1**. A substituted 2,6,8,10-tetra-methyl-8-hydroxy naphthalene 1,4,7-trione skeleton was deduced based on its spectroscopic features, combining with the key HMBC correlations from H-3 to C-1 and C-5, from H_3_-18 to C-1, C-2, and C-3, from H_3_-19 to C-4, C-5, C-6, and C-7, from H_3_-20 to C-7, C-8, and C-9, and from H_3_-21 to C-1, C-5, C-9, and C-10 (Figure 2). A typical sorbyl side chain was deduced from the ^1^H-^1^H COSY correlations H-13/H-14/H-15/H-16/H_3_-17 and HMBC correlation from H-14 to C-12 (Figure 2). Further, the ^1^H-^1^H COSY signal H-9/H_2_-11 and the HMBC correlations from H_2_-11 to C-8, C-9, C-10, and C-12 (Figure 2) demonstrated that the sorbyl side chain was connected to C-9 bridged by CH_2_-11.

**FIGURE 3:**
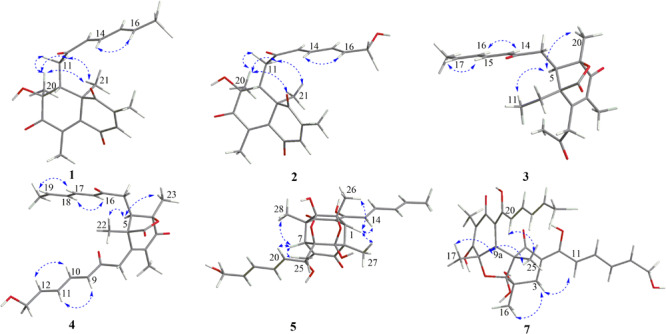
Key NOESY correlations of **1**–**5** and **7**.

**FIGURE 4:**
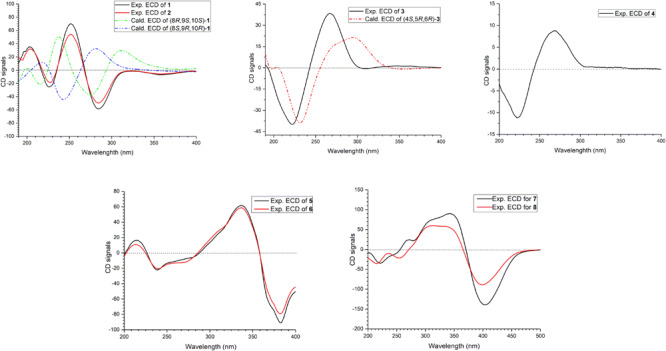
ECD spectra of compounds **1**–**8**.

The relative configuration of **1** was determined by coupling constants and NOESY spectrum (Figure 3). The *E*-configurations of double bonds in the sorbyl side chain were deduced on the basis of the large coupling constant (*J*_H–__13__/H–__14_ = 15.5 Hz) and the NOESY correlation between H-14 and H-16. The NOESY correlation between H_3_-20 and H_3_-21 indicated the same orientation of these two methyls, whereas the protons H_3_-20 and H_3_-21 were simultaneously correlated with H_2_-11, reflecting that proton H-9 should locate at the other orientation. Therefore, the relative configuration of **1** was assumed as 8*R*^∗^,9*S*^∗^,10*S*^∗^. The absolute stereochemistry of **1** was resolved by quantum chemical time-dependent density functional theory (TDDFT) calculation of its electronic circular dichroism (ECD) spectra of (8*R*,9*S*,10*S*)-**1** and (8*S*,9*R*10*R*)-**1**. The experimental ECD spectrum of **1** displayed the intense positive cotton effect at 252 nm and negative cotton effects at 227 and 288 nm, respectively, which were consistent with the theoretical ECD spectrum for (8*R*,9*S*,10*S*)-**1** (Figure 4). Therefore, the absolute configuration of **1** was determined as 8*R*,9*S*,10*S*.

Trichoreeseione B (**2**) was assigned a molecular formula of C_21_H_24_O_6_ by HR-ESIMS, showing one additional oxygen atom compared to that of **1**. The NMR data of **2** (Table 1) suggested the same skeleton as **1**, apart from a hydroxy-methylene resonance (δ_H_ 4.31, δ_*C*_ 62.9) in **2** instead of the methyl signals (δ_H_ 1.87, δ_*C*_ 18.9) in **1**. The ^1^H-^1^H COSY and HMBC correlations confirmed the planar structure of **2**, in which a terminal hydroxy-methylene in the sorbyl side chain replaced the methyl group in **1** (Figure 2). The coupling constants, NOESY correlations (Figure 3), and ECD spectrum of **2** (Figure 4) demonstrated that its stereochemistry was the same as **1**. It is worth mentioning that compounds **1** and **2** represented the first example of sorbicillinoids with a characteristic naphthalene-trione ring.

Trichodermolide B (**3**) was given a molecular formula C_21_H_26_O_5_ based on HR-ESIMS, indicating 9 degrees of unsaturation. The ^1^H NMR and ^13^C NMR data (Table 2) showed four carbonyls, three pairs of olefinic carbons, five methyls, three methylenes, one methine, and two quaternary carbons. These spectroscopic features suggested that **3** should be a sorbicillinoid analog and closely resembled to trichodermolide isolated from *Trichoderma longibrachiatum* (strain UAMH 4159) collected from cotton duck shelter (Andrade et al., 1996). The main differences were absence of four olefinic carbons, and addition of a propan-2-one moiety and one methylene. The HMBC correlations from H_2_-7 to C-2, C-3 and C-4 suggested the propan-2-one moiety was attached to C-3. The ^1^H-^1^H COSY linkage between H_2_-10 and H_3_-11 indicated the presence of one ethylene group, which was located at C-4 based on the HMBC correlations from H_3_-11 to C-4, and from H_2_-10 to C-4, C-5, and C-21 (Figure 2).

The two double bonds in sorbyl side chain of **3** were also assigned as *E* configuration on the basis of their coupling constants (*J*_H–__14__/H–__15_ = 15.5 Hz), and the NOESY correlation between H-14/H-16 and H-15/H-17 (Figure 3). The relative configurations of the stereocenters at C-4, C-5, and C-6 were determined by NOESY spectrum. The NOESY correlations of H-5/H_3_-20, and H-5/H_3_-11 suggested the same side of these protons. Therefore, the relative configuration of **3** was assumed as 4*S*^∗^,5*R*^∗^,6*R*^∗^. The absolute configuration of **3** was determined by ECD calculation. The Boltzmann-weighted ECD curve of (4*S*,5*R*,6*R*)**-3** agreed with the experimental one (Figure 4), and hence, the absolute configuration of **3** was assigned as 4*S*,5*R*,6*R*.

13-Hydroxy-trichodermolide (**4**) has a molecular formula C_24_H_28_O_6_ deduced by HR-ESIMS *m*/*z* 413.1956 [M + H]^+^ (calcd. for C_24_H_29_O_6_, 413.1959), indicating 11 degrees of unsaturation. The NMR data of **4** (Table 2) were very similar to those of trichodermolide isolated from *T. longibrachiatum* (strain UAMH 4159) (Andrade et al., 1996), except the terminal methyl group (δ_H_ 1.88, δ_C_ 18.9) at C-13 in trichodermolide was substituted by a hydroxy methylene group (δ_H_ 4.34, δ_C_ 62.6) in **4**.

The *E* configurations among the four double bonds in the side chains of **4** were confirmed by large coupling constant values (*J*_H–__9__/H–__10_ = *J*_H–__11__/H–__12_ = *J*_H–__16__/H–__17_ = 15.5 Hz) and the NOESY correlation between H-19/H-17 and H-16/H-18 (Figure 3). The relative configurations of three stereocenters, C-4, C-5, and C-6 of **4** were determined by comparison of its ^1^H NMR data with already reported compound dihydro-trichodermolide (Li et al., 2011). The similarity of chemical shifts of H-5 (δ_H_ 3.33), H_2_-14 (δ_H_ 3.23 and 2.46), and two methyl groups H_3_-22 (1.23) and H_3_-23 (δ_H_ 1.46) in **4** with those of in dihydro-trichodermolide, combing the NOESY correlations between H-5/H_3_-22 and H-5/H_3_-23, established that the relative configuration of **4** was in accordance with that of dihydro-trichodermolide. The absolute configuration of **4** was determined by comparing its ECD data (Figure 4) with those of known analogs (Andrade et al., 1996; Li et al., 2011; Cao et al., 2019). The ECD spectrum of **4** demonstrated nearly similar negative and positive cotton effects to those of reported analogs (Figure 4 and Supplementary Table S4). Consequently, the absolute configuration of **4** was determined as 4*S*,5*R*,6*R*.

A literature survey revealed that bicycle (3.2.1) lactone skeleton was rare in the sorbicillinoid family. So far, only three compounds with this distinctive skeleton have been reported, including trichodermolide (Andrade et al., 1996), dihydro-trichodermolide (Li et al., 2011) and 13-hydroxy-dihydro-trichodermolide (Cao et al., 2019). In this study, two new sorbicillinoids (**3** and **4**) with a bicycle [3.2.1] lactone skeleton was discovered. Among them, **3** was unique in a sense that the propan-2-one side chain was reported first time for sorbicillinoid family.

24-Hydroxy-trichodimerol (**5**) was assigned the molecular formula C_28_H_32_O_9_ by HR-ESIMS with 13 degrees of unsaturation. The NMR spectra (Table 3) of **5** exhibited resembling resonances with those of the co-isolated known compound trichodimerol (**6**), which was firstly isolated from *T. longibrachiatum* (strain UAMH 4159) (Andrade et al., 1992). The only obvious difference was the terminal methyl group (δ_H_ 1.89 δ_C_18.8) at C-24 of sorbyl residue in **6** was replaced by a hydroxylated methylene group (δ_H_ 4.22, δ_C_ 62.9) in **5**.

The stereochemistry of the double bonds in the sorbyl chains in **5** was assigned as *E* configurations based on their coupling constants (*J*_H–__14__/H–__15_ = *J*_H–__16__/H–__17_ = *J*_H–__20__/H–__21_ = *J*_H–__22__/H–__23_ = 14.7 Hz). Moreover, in the NOESY spectrum, the correlations between H-1 and H-14, H_3_-26, H_3_-27, respectively, and between H-7 and H-20, H_3_-25, H_3_-28, respectively, were observed (Figure 3), suggesting the same relative configurations of **5** and **6**. The absolute configuration of **5** was determined by comparing its ECD spectrum with **6**. Compound **5** displayed strong positive Cotton effect at 338 nm (+62) and negative Cotton effect at 383 nm (−91), which was similar with that of **6** (Figure 4). Therefore, the absolute configuration of **5** was assumed as 1*R*,4*R*,5*R*,6*S*,7*R*,10*R*,11*R*,12*S* (Figure 1). It is worth noting that bisorbicillinoids possessing open-ended cage skeleton were uncommon active compounds (Zhai et al., 2016), of which trichodimerol was biomimetic total synthesized by Nicolaou et al. (2000).

15-Hydroxy-bisvertinol (**7**) displayed a [M − H]^–^ ion at *m/z* 513.2136 in its HR-ESIMS, in accordance with the molecular formula C_28_H_33_O_9_, which indicated 12 degrees of unsaturation. The 1D NMR and HSQC spectra displayed the presence of five methyls, two methylenes, nine methines, and twelve quaternary carbons. Careful examination of the NMR data of **7** (Table 3) disclosed that its skeleton has resemblance with the known compound bisvertinol (**9**), primarily isolated from fungus *Verticillium* intertextum (Trifonov et al., 1986). The obvious difference was that the methyl group at C-15 on one of the sorbyl side chains in **9** was replaced by a hydroxy methylene in **7**.

The relative configuration of **7** was addressed by the coupling constants, NOESY spectrum and biogenetic relationship. The large coupling constants of the four double bonds in the sorbyl chains (*J*_H–__1__1__/H–__1__2_ = *J*_H–__1__3__/H–__1__4_ = *J*_H–__20__/H–__21_ = *J*_H–__22__/H–__23_ = 14.7 Hz) reflected their *E*-configurations. In the NOESY spectrum, key cross peaks were observed between H-9a and H_3_-25 and H_3_-17 (Figure 3), indicating that the methyls H_3_-17 and H_3_-25 were at the same side with H-9a. The 4a-OH was deduced in the same face with H-9a, H_3_-25 and H_3_-17 by considering the higher stability of a *cis* 5-6 ring junction over a *trans* 5-6 ring junction (Andrade et al., 1992). The configuration of C-4 was presumed to be identical to that of the co-isolated **8** and **9** on the basis of biogenetic relationship. The experimental ECD spectra of **7** and **8** (Figure 4) were parallel to each other, possessing positive Cotton effect at 346 nm and negative at 403 nm, and both gave negative optical rotation values. Consequently, the absolute configuration of **7** was deduced as 4*S*,4a*R*,5a*S*,9a*R*,9b*R*.

The structures of known compounds, trichodermol (**6**) (Andrade et al., 1992), 24-hydroxy-bisvertinol (**8**) (Zhang et al., 2019), and bisvertinol (**9**) (Trifonov et al., 1986), were recognized by comparing their spectroscopic data (^1^H and ^13^C NMR, and MS) with those reported in the literature.

### Bioassays of Compounds

**FIGURE 5:**
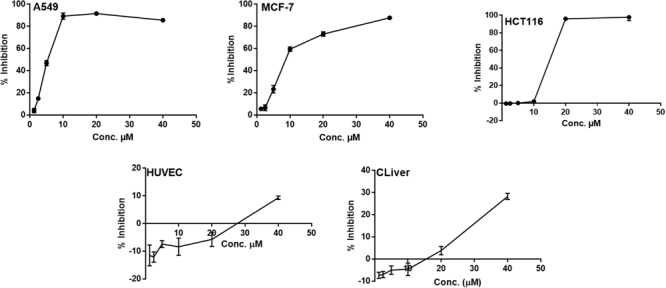
The dose response curve of the cytotoxicity of compound **5** toward A549, MCF-7 HCT 116, HUVEC and CLiver cell lines. Cells were exposed to compound **5** with different concentrations for 72 h. Cell inhibition was determined by SRB stain.

All of the isolated new compounds (**1**–**5** and **7**) were assessed for their cytotoxic activities against five human tumor cell lines, including A549, HepG2, HCT 116, HeLa, MCF-7 and two healthy human cell lines HUVEC and CLiver. Compound **5** displayed cytotoxic activity against A549, MCF-7 and HCT 116 cell lines (Figure 5) with the IC_50_ values of 5.1, 9.5 and 13.7 μM, respectively, whereas displayed no significant cytotoxic activity against normal cell lines (HUVEC and CLiver) with the IC_50_ values higher than 40 μM. The selectivity index values (SI, IC_50_ normal cell line/IC_50_ cancer cell line) for compound **5** were found to be higher than 7.8, 4.2, and 2.9, respectively, indicating its selective cytotoxicity.

## Conclusion

**FIGURE 6:**
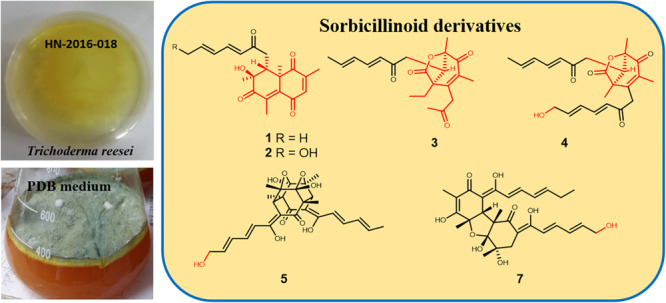
Structural features of isolated sorbicillinoids.

In summary, we report here nine sorbicillinoid derivatives (**1**–**9**), including six new compounds, isolated from the sponge-derived fungus *T. reesei* (HN-2016-018). To date, more than 130 sorbicillinoid derivatives have been reported (Meng et al., 2016, 2018, 2019; Zhang et al., 2018, 2019; Cao et al., 2019; Wang et al., 2019; Yu et al., 2019). In this study, we discovered two novel sorbicillinoids (**1**–**2**) with a characteristic naphthalene-trione ring and two rare sorbicillinoids (**3**–**4)** possessing a bicycle (3.2.1) lactone skeleton (Figure 6), where only three compounds with such distinctive skeleton have been reported previously. Furthermore, compound **3** represented the first reported sorbicillinoid with a propan-2-one side chain, while a terminal hydroxylation at the side chain of compounds **2**, **4**, **5,** and **7** was also rare in the sorbicillinoid family. Compound **5** displayed strong cytotoxic activity against A549, MCF-7, and HCT116 cell lines. This study enriched the structural diversity of sorbicillinoids and provided the chemical entities for the development of marine bioactive natural products.

## Data Availability Statement

The original contributions presented in the study are included in the article/Supplementary Material, and further inquiries can be directed to the corresponding author/s.

## Author Contributions

C-YW and C-LS conceived of and proposed the idea. SR contributed to fermentation, extraction, and isolation. SR and L-JY contributed to the manuscript preparation. WH contributed to bioactivities test. SR, L-JY, J-SW, TS, and Y-HZ contributed to data analysis, write up, revision, and proofreading of the manuscript. All authors read and approved the final manuscript.

## Conflict of Interest

The authors declare that the research was conducted in the absence of any commercial or financial relationships that could be construed as a potential conflict of interest.
