# Supplementary material for: Sorbicillinoid Derivatives From Sponge-Derived Fungus Trichoderma reesei (HN-2016-018)
Source: Front Microbiol. 2020 Jun 23;11:1334. doi: 10.3389/fmicb.2020.01334 (PMC7325520; doi:10.3389/fmicb.2020.01334)
Supplement: Supplementary file 1 [file Data_Sheet_1.doc]

Supplementary Material

**Sorbicillinoid derivatives from sponge-derived fungus *Trichoderma reesei* (HN-2016-018)**

**Saif Ur Rehman,^1,2,3†^ Lu-Jia Yang,^1,2†^ Ya-Hui Zhang,^1,2^ Jing-Shuai Wu,^1,2^ Ting Shi,****^1,2^ Waqas Haider,^1,2^ Chang-Lun Shao,^1,2^ Chang-Yun Wang^1,2*^**

^1^Key Laboratory of Marine Drugs, The Ministry of Education of China, School of Medicine and Pharmacy; Institute of Evolution & Marine Biodiversity, Ocean University of China, Qingdao 266003, People’s Republic of China

^2^Laboratory for Marine Drugs and Bioproducts, Qingdao National Laboratory for Marine Science and Technology, Qingdao 266237, People’s Republic of China

^3^Department of Pharmacy, Faculty of Medical & Health sciences, University of the Poonch, Rawalakot, District Poonch 12350, Azad Jammu & Kashmir, Pakistan

* **Correspondence:** changyun@ouc.edu.cn. Tel. +86-532-8203-1536 (C.Y.W.); Fax: +86-532-8203-1536 (C.Y.W.)

^†^These authors contributed equally to this work.

**List of Supporting Information**

**Figure S1**. ^1^H NMR (600 MHz, CDCl_3_) spectrum of trichoreeseione A (**1**)

**Figure S2**. Partial enlarged drawing of the ^1^H NMR spectrum of trichoreeseione A (**1**)

**Figure S3**. ^13^C NMR (150 MHz, CDCl_3_) spectrum of trichoreeseione A (**1**)

**Figure S4**. HSQC spectrum of trichoreeseione A (**1**) in CDCl_3_

**Figure S5**. ^1^H-^1^H COSY spectrum of trichoreeseione A (**1**) in CDCl_3_

**Figure S6**. HMBC spectrum of trichoreeseione A (**1**) in CDCl_3_

**Figure S7**. NOESY spectrum of trichoreeseione A (**1**) in CDCl_3_

**Figure S8**. HR-ESIMS spectrum of trichoreeseione A (**1**)

**Figure S9**. ^1^H NMR (600 MHz, CDCl_3_) spectrum of trichoreeseione B (**2**)

**Figure S10**. ^13^C NMR (150 MHz, CDCl_3_) spectrum of trichoreeseione B (**2**)

**Figure S11**. HSQC spectrum of trichoreeseione B (**2**) in CDCl_3_

**Figure S12**.^1^H-^1^H COSY spectrum of trichoreeseione B (**2**) in CDCl_3_

**Figure S13**. HMBC spectrum of trichoreeseione B (**2**) in CDCl_3_

**Figure S14**. NOESY spectrum of trichoreeseione B (**2**) in CDCl_3_

**Figure S15**. HR-ESIMS spectrum of trichoreeseione B (**2**)

**Figure S16**. ^1^H NMR (600 MHz, DMSO-*d_6_*) spectrum of trichodermolide B (**3**)

**Figure S17**. ^13^C NMR (150MHz, DMSO-*d_6_*) spectrum of trichodermolide B (**3**)

**Figure S18**. HSQC spectrum of trichodermolide B (**3**) in DMSO-*d_6_*

**Figure S19**. ^1^H-^1^H COSY spectrum of trichodermolide B (**3**) in DMSO-*d_6_*

**Figure S20**. HMBC spectrum of trichodermolide B (**3)** in DMSO-*d_6_*

**Figure S21**. NOSEY spectrum of trichodermolide B (**3)** in DMSO-*d_6_*

**Figure S22**. HR-ESIMS spectrum of trichodermolide B (**3**)

**Figure S23**. ^1^H NMR (600 MHz, CDCl_3_) spectrum of 13-hydroxy-trichodermolide (**4**)

**Figure S24**. Partial enlarged drawing of the ^1^H NMR spectrum of 13-hydroxy-trichodermolide (**4**)

**Figure S25**. ^13^C NMR (150MHz, CDCl_3_) spectrum of 13-hydroxy-trichodermolide (**4**)

**Figure S26**. HSQC spectrum of 13-hydroxy-trichodermolide (**4**) in CDCl_3_

**Figure S27**. ^1^H-^1^H COSY spectrum of 13-hydroxy-trichodermolide (**4**) in CDCl_3_

**Figure S28**. HMBC spectrum of 13-hydroxy-trichodermolide (**4**) in CDCl_3_

**Figure S29**. NOESY spectrum of 13-hydroxy-trichodermolide (**4**) in CDCl_3_

**Figure S30**. HR-ESIMS spectrum of 13-hydroxy-trichodermolide (**4**)

**Figure S31**. ^1^H NMR (600 MHz, CD_3_OD) spectrum of 24-hydroxy-trichodimerol (**5**)

**FigureS32**. Partial enlarged drawing of the ^1^H NMR spectrum of 24-hydroxy-trichodimerol (**5**)

**Figure S33**. ^13^C NMR (150 MHz, CD_3_OD) spectrum of 24-hydroxy-trichodimerol (**5**)

**Figure S34**. HSQC spectrum of 24-hydroxy-trichodimerol (**5**) in CD_3_OD

**Figure S35**. ^1^H-^1^H COSY spectrum of 24-hydroxy-trichodimerol (**5**) in CD_3_OD

**Figure S36**. HMBC spectrum of 24-hydroxy-trichodimerol (**5**) in CD_3_OD

**Figure S37**. NOESY spectrum of 24-hydroxy-trichodimerol (**5**) in CD_3_OD

**Figure S38**. HR-ESIMS spectrum of 24-hydroxy-trichodimerol (**5**)

**Figure S39**. ^1^H NMR (400 MHz, CD_3_OD) spectrum of 15-hydroxy-bisvertinol (**7**)

**Figure S40**. Partial enlarged drawing of the ^1^H NMR spectrum of 15-hydroxy-bisvertinol (**7**)

**Figure S41**. ^13^C NMR (100MHz, CD_3_OD) spectrum of15-hydroxy-bisvertinol (**7**)

**Figure S42**. HSQC spectrum of 15-hydroxy-bisvertinol (**7**) in CD_3_OD

**Figure S43**. ^1^H-^1^H COSY spectrum of 15-hydroxy-bisvertinol (**7**) in CD_3_OD

**Figure S44**. HMBC spectrum of 15-hydroxy-bisvertinol (**7**) in CD_3_OD

**Figure S45**. NOESY spectrum of 15-hydroxy-bisvertinol (**7**) in CD_3_OD

**Figure S46**. HR-ESIMS spectrum of 15-hydroxy-bisvertinol (**7**)

**Figure S47**. Structures and population of the low-energy B3LYP/6-311+G(d) conformers (>5%)

of (8*R*,9*S*,10*S*)-**1**

**Figure S48**. Structures and population of the low-energy B3LYP/6-311+G(d) conformers (>5%)

of (8*S*,9*R*,10*R*)-**1**

**Figure S49**. Structures and population of the low-energy B3LYP/6-311+G(d) conformers (>5%)

of (4*S*,5*R*,6*R*)-**3**

**Figure S50**. The neighbor-joining phylogenetic tree of fungal strain *T.reesei* (HN-2016-018)

**Table S1**. Cartesian coordinates of the low-energy conformers (≥5%) of (8*R*,9*S*,10*S*)-**1**

**Table S2**. Cartesian coordinates of the low-energy conformers (≥5%) of (8*S*,9*R*,10*R*)-**1**.

**Table S3**. Cartesian coordinates of the low-energy conformers (≥5%) of (4*S*,5*R*,6*R*)-**3**.

**Table S4**. The CD data of 13-hydroxy-trichodermolide (**4**), and the reported trichodermolide, dihydro-trichodermolide, and 13-hydroxy-dihydro-trichodermolide

**Figure S1**. ^1^H NMR (600 MHz, CDCl_3_) spectrum of trichoreeseione A (**1**)


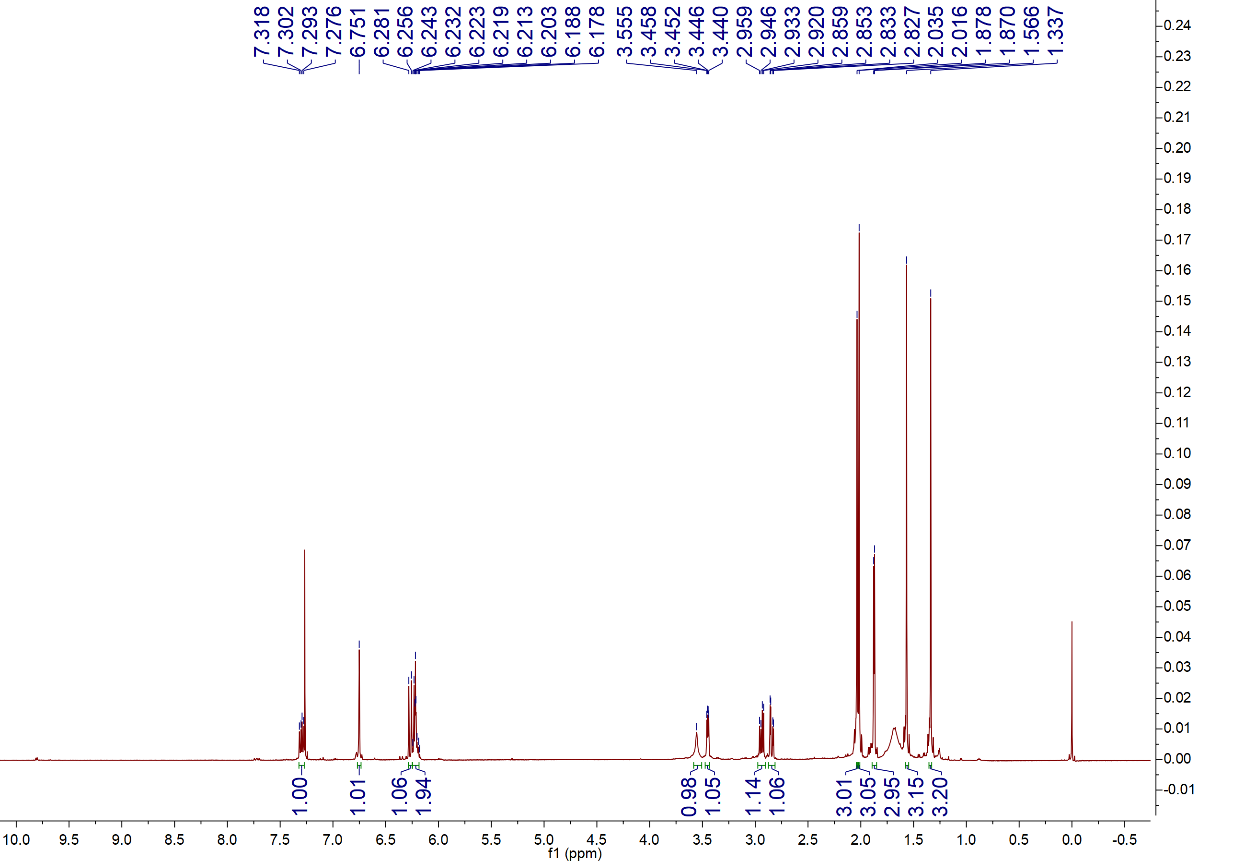


**Figure S2.** Partial enlarged drawing of the ^1^H NMR spectrum of trichoreeseione A (**1**)


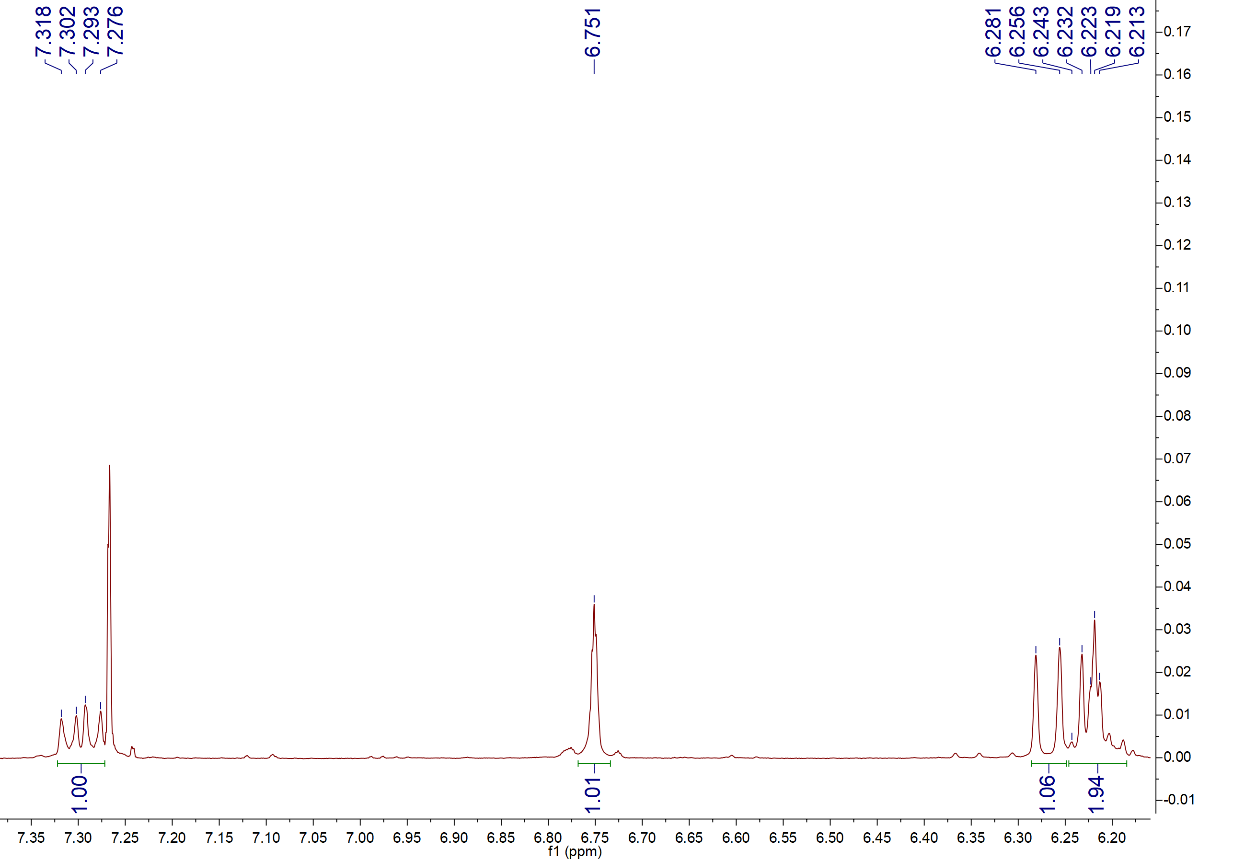


**Figure S3**.^13^C NMR (150 MHz, CDCl_3_) spectrum of trichoreeseione A (**1**)


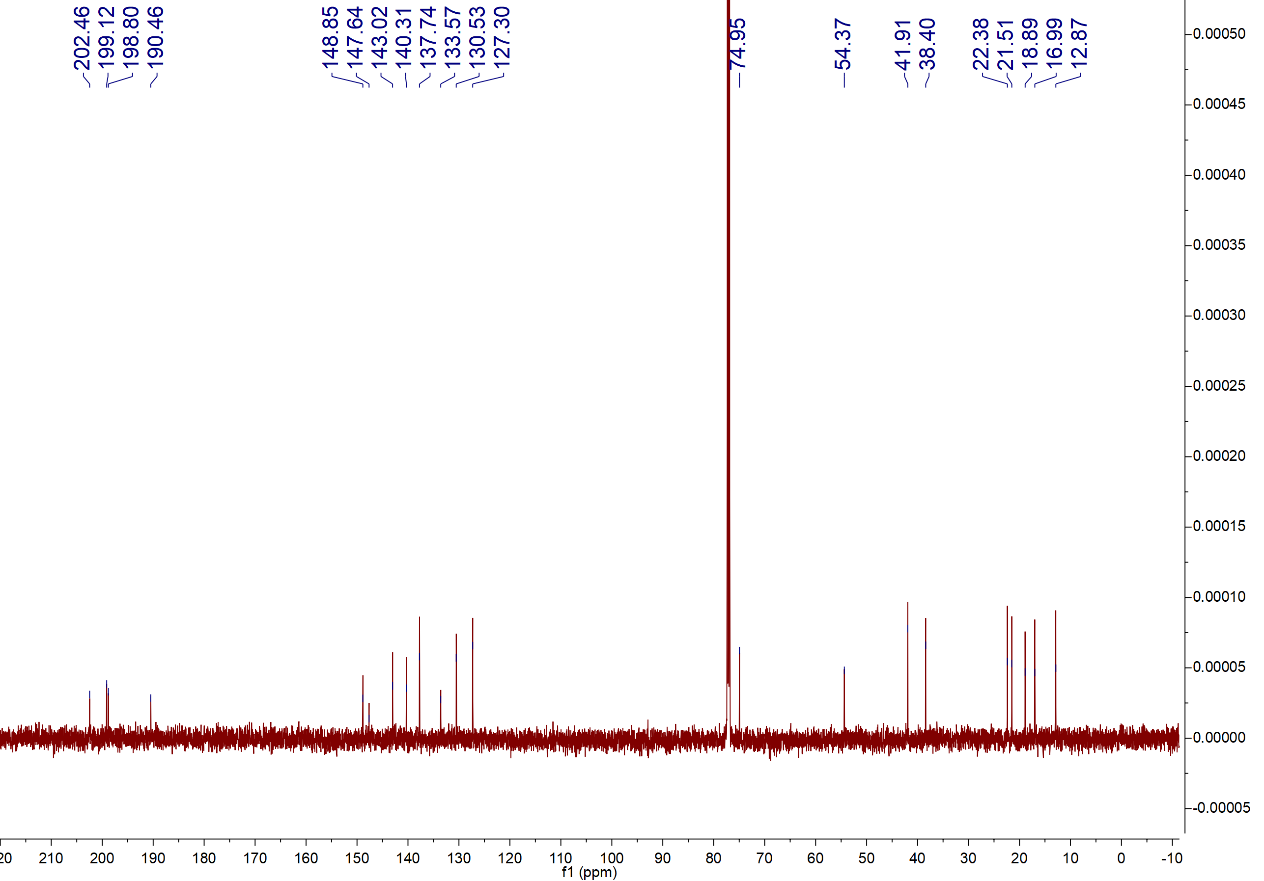


**Figure S4**. HSQC spectrum of trichoreeseione A (**1**) in CDCl_3_


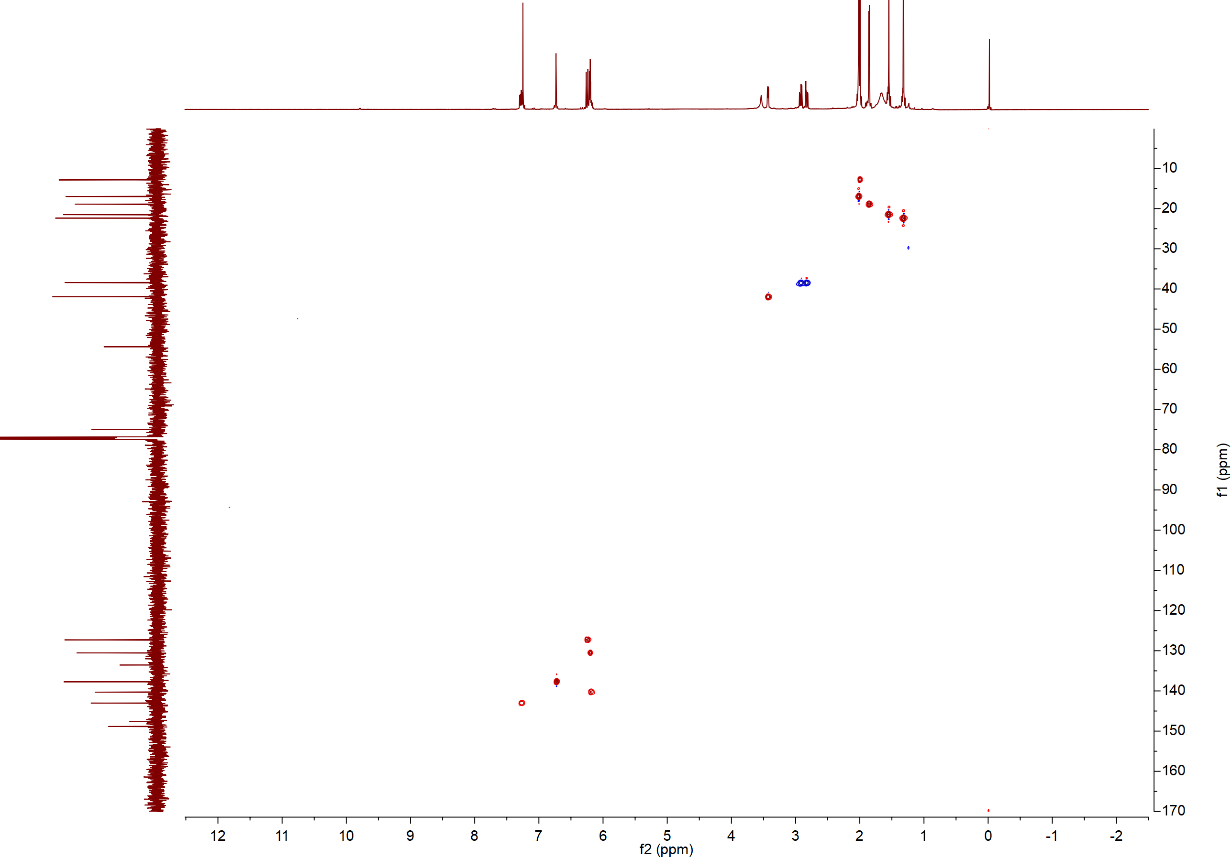


**Figure S5**. ^1^H-^1^H COSY spectrum of trichoreeseione A (**1**) in CDCl_3_


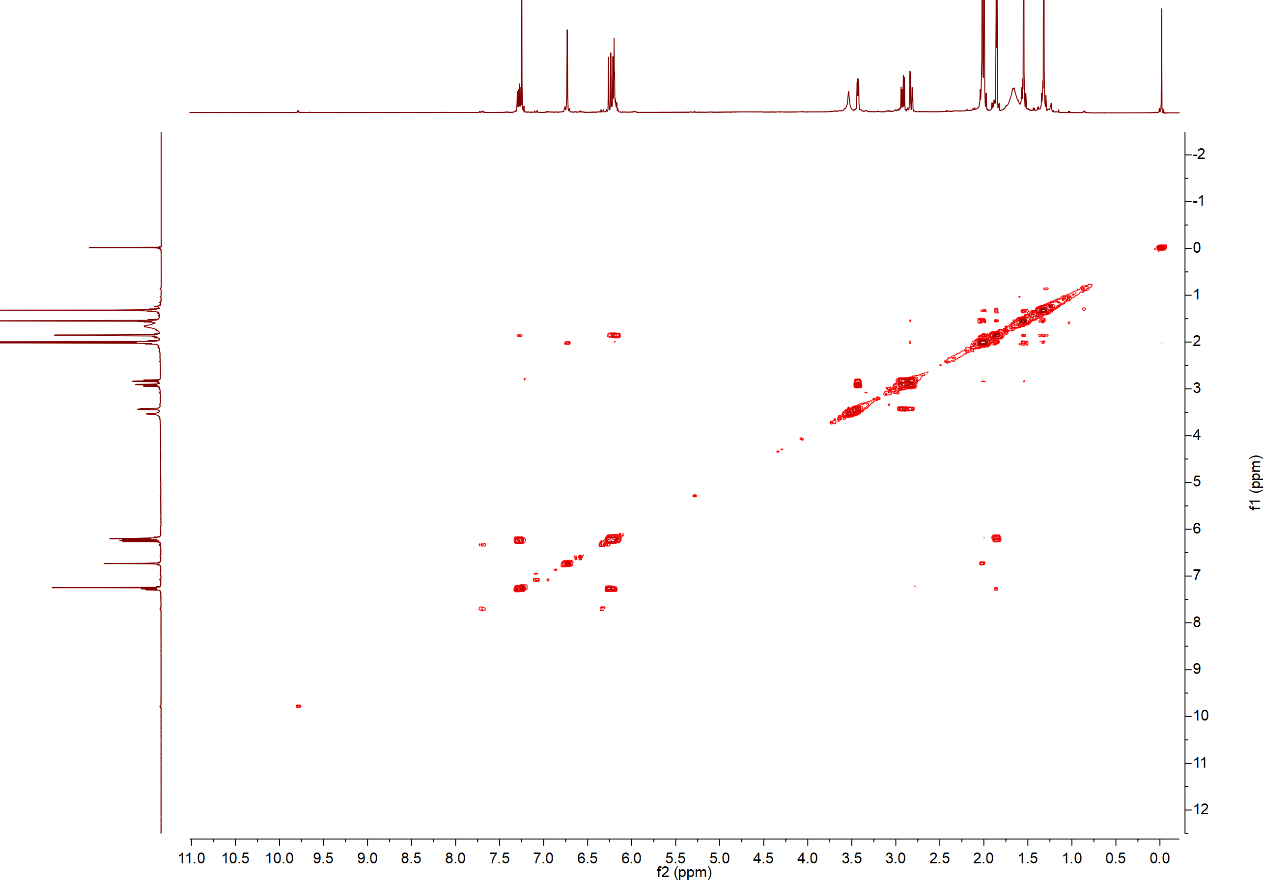


**Figure S6**. HMBC spectrum of trichoreeseione A (**1**) in CDCl_3_


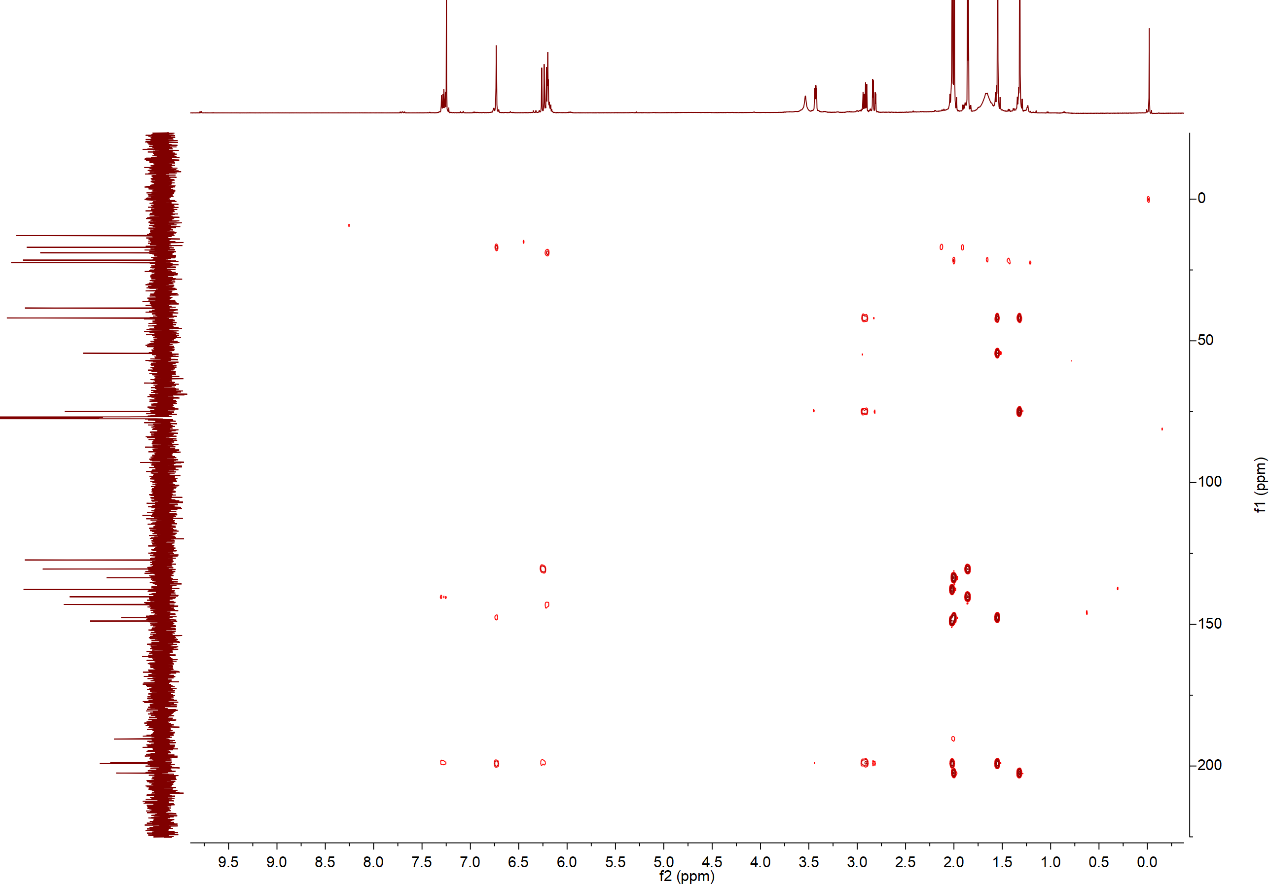


**Figure S7**. NOESY spectrum of trichoreeseione A (**1**) in CDCl_3_


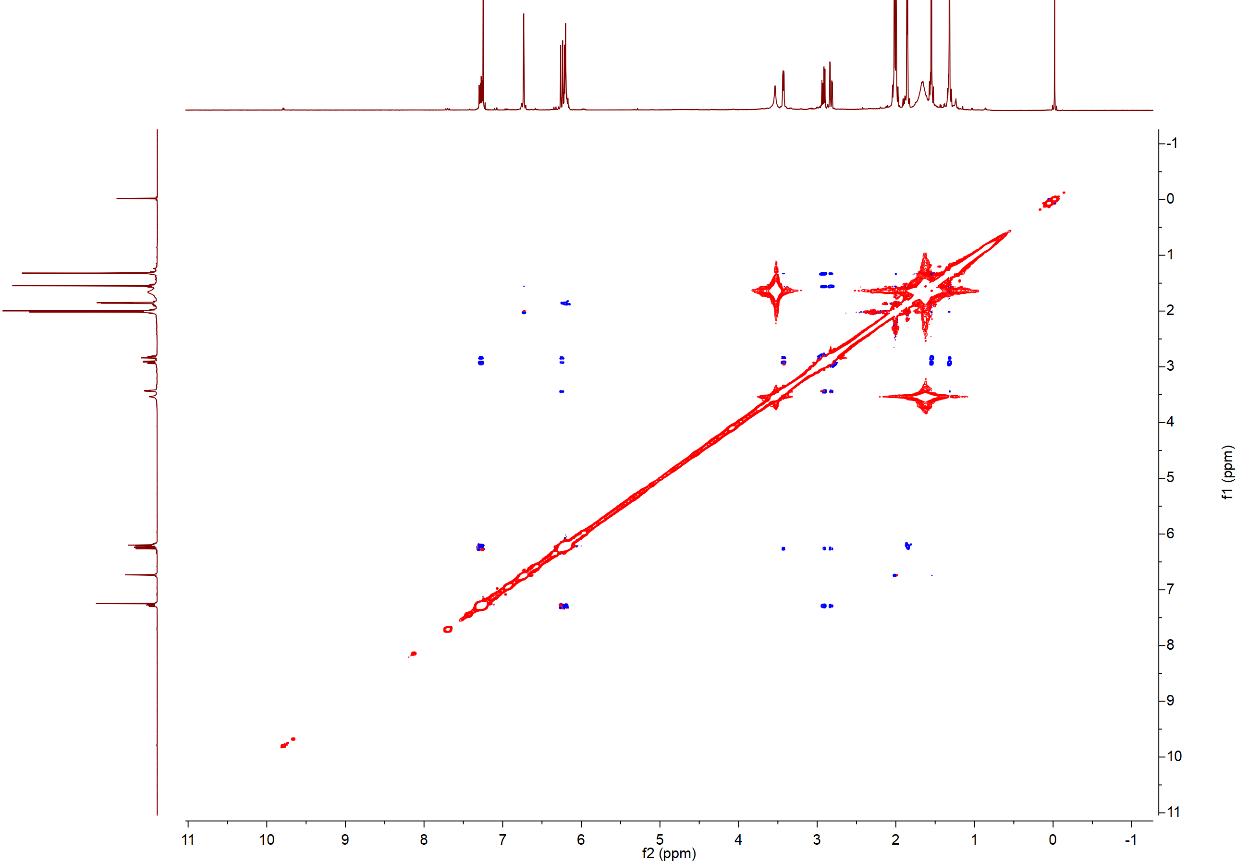


**Figure S8**. HR-ESIMS spectrum of trichoreeseione A (**1**)

**Figure S9**. ^1^H NMR (600 MHz, CDCl_3_) spectrum of trichoreeseione B (**2**)


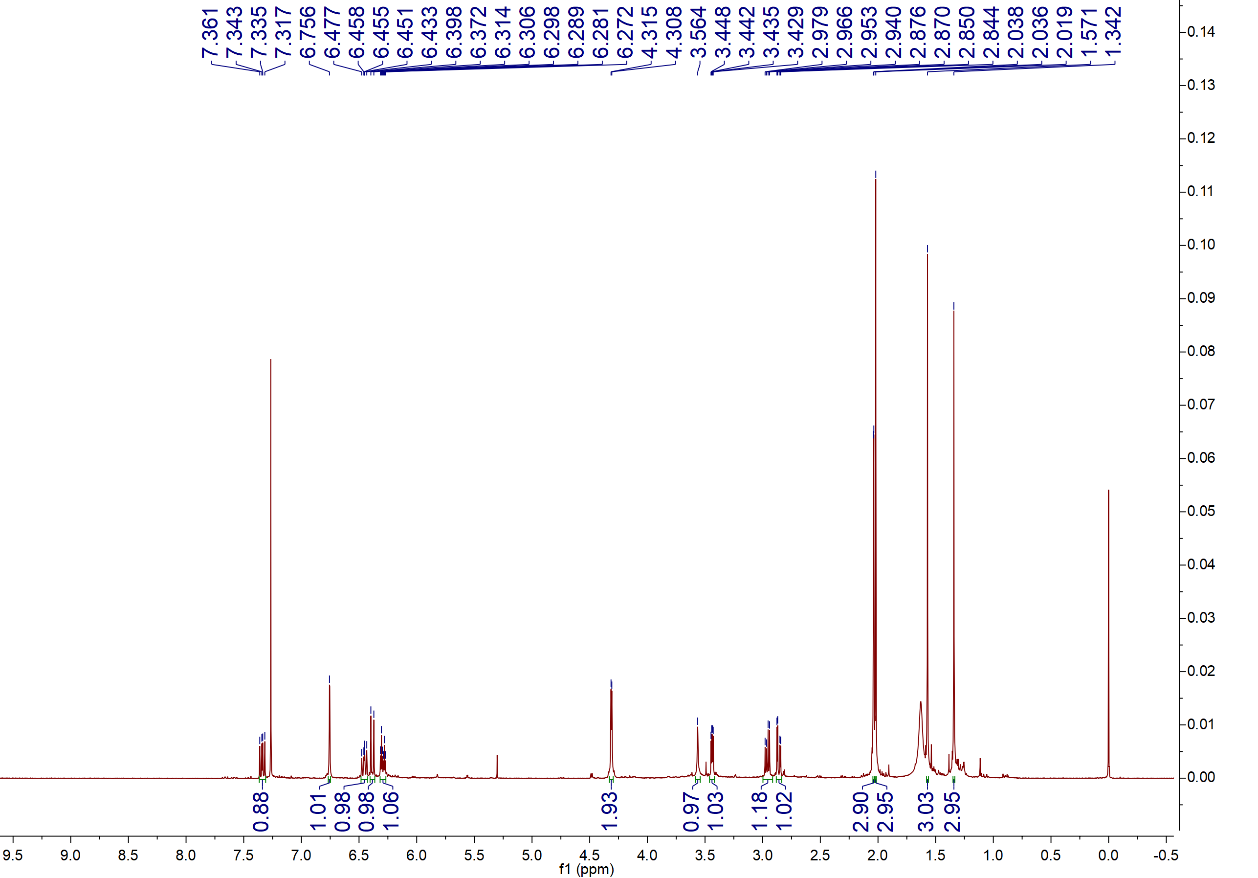


**Figure S10**. ^13^C NMR (150 MHz, CDCl_3_) spectrum of trichoreeseione B (**2**)


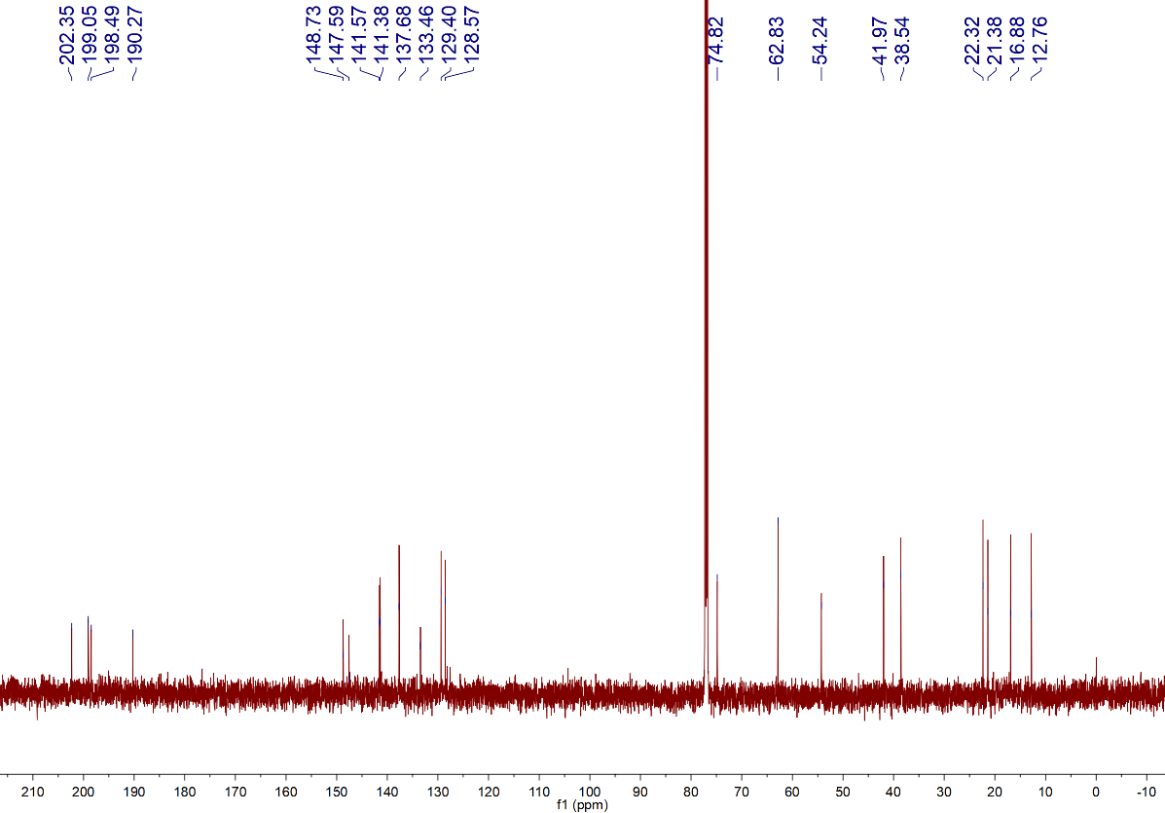


**Figure S11**. HSQC spectrum of trichoreeseione B (**2**) in CDCl_3_


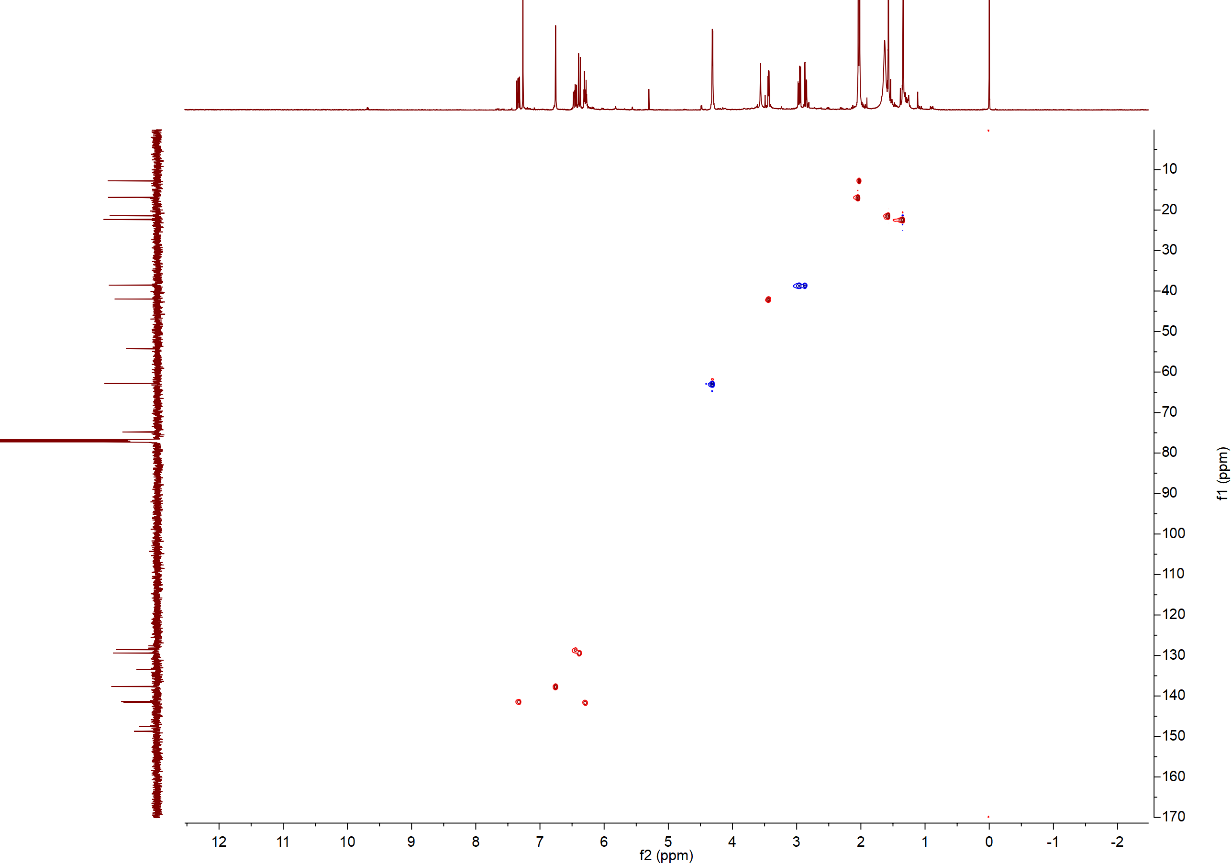


**Figure S12**. ^1^H-^1^H COSY spectrum of trichoreeseione B (**2**) in CDCl_3_


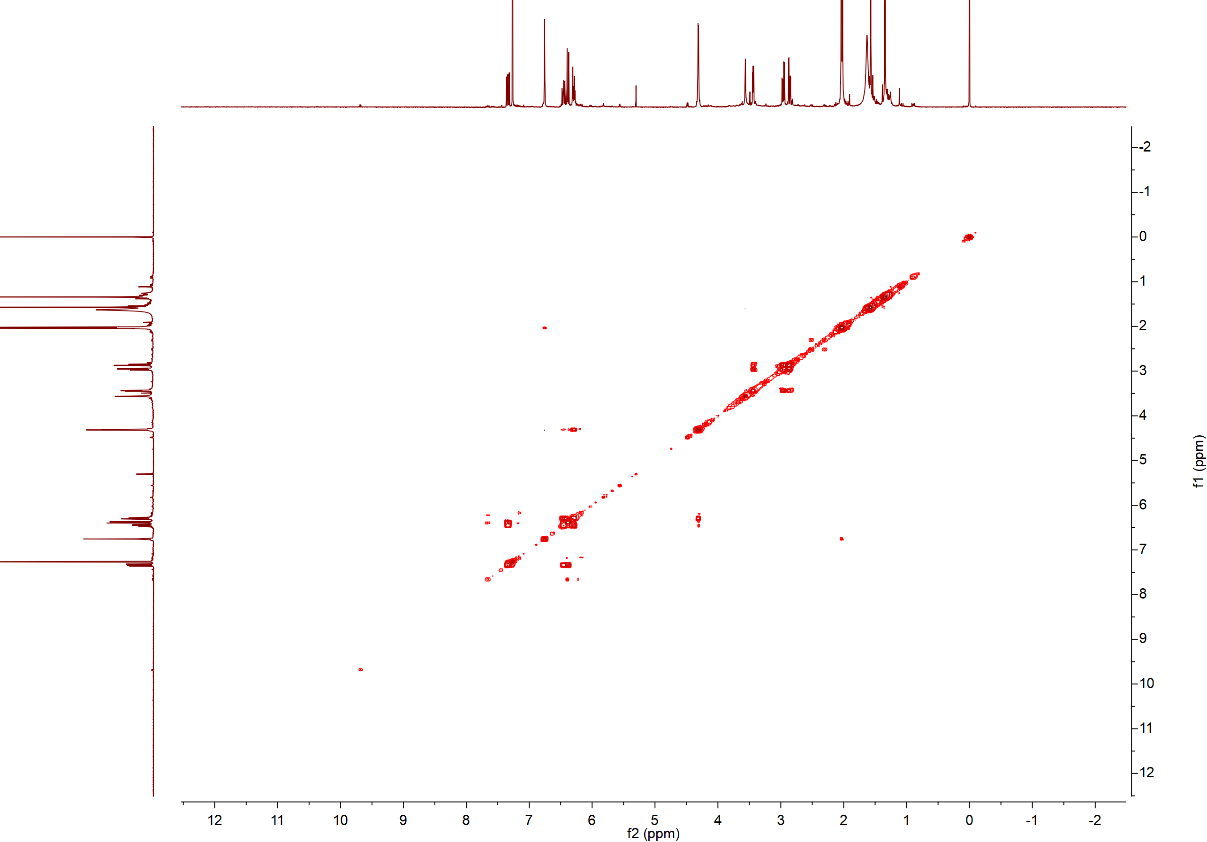


**Figure S13**. HMBC spectrum of trichoreeseione B (**2**) in CDCl_3_


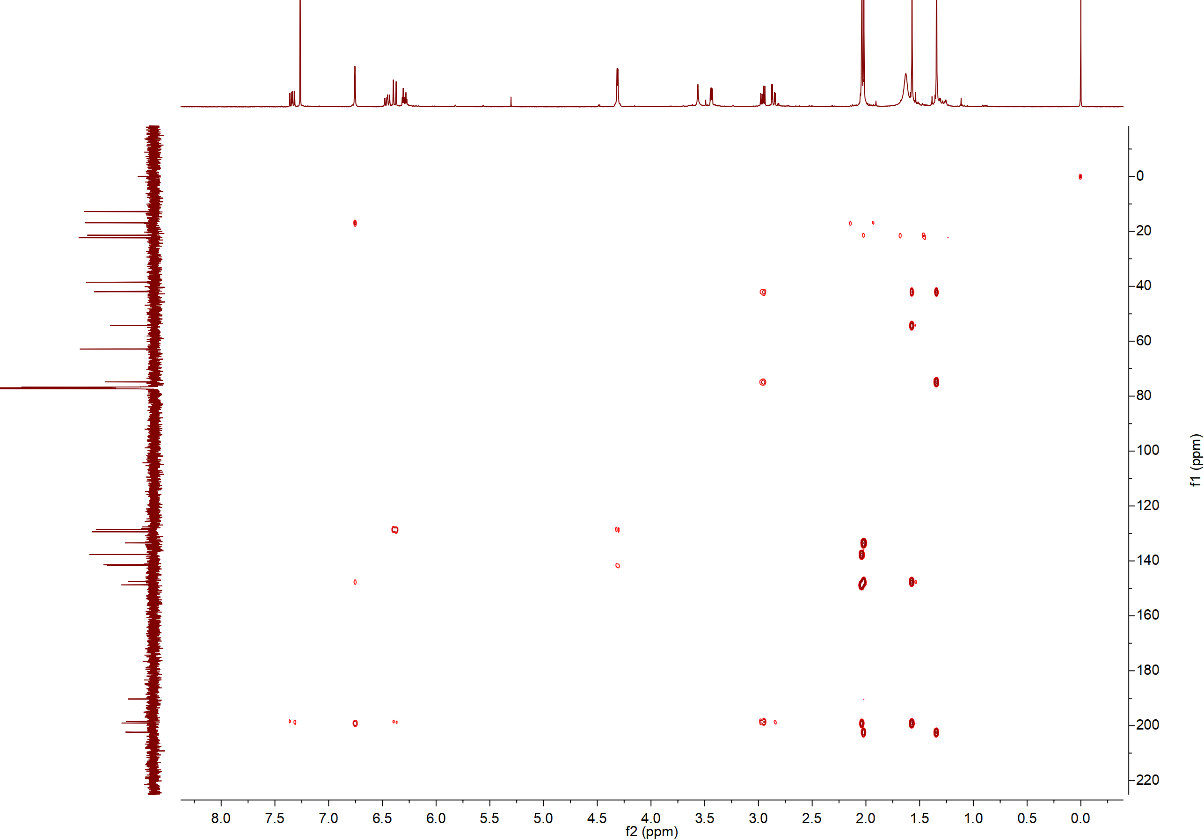


**Figure S14**. NOESY spectrum of trichoreeseione B (**2**) in CDCl_3_


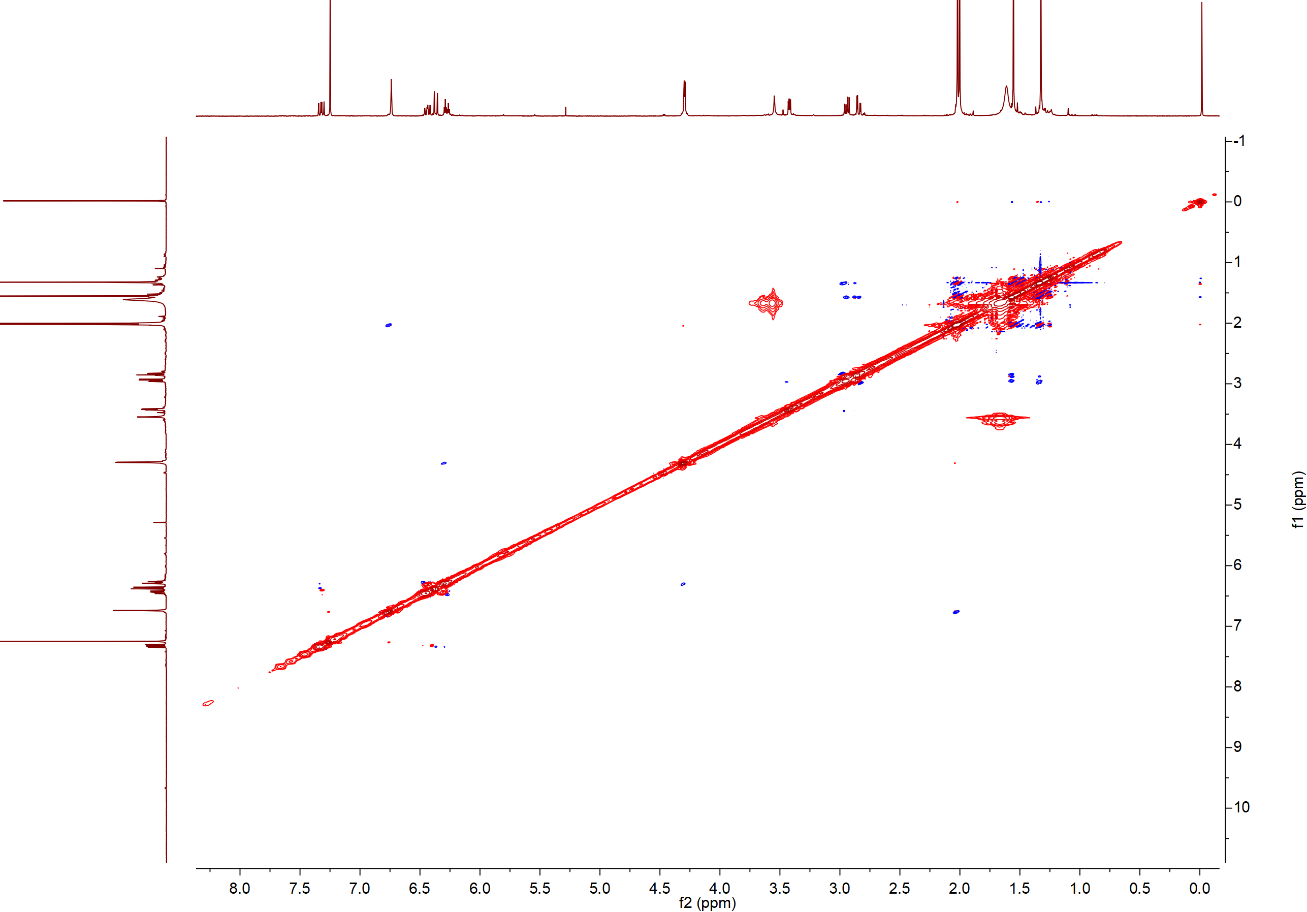


**Figure S15**. HR-ESIMS spectrum of trichoreeseione B (**2**)

**Figure S16**. ^1^H NMR (600 MHz, DMSO-*d_6_*) spectrum of trichodermolide B (**3**)


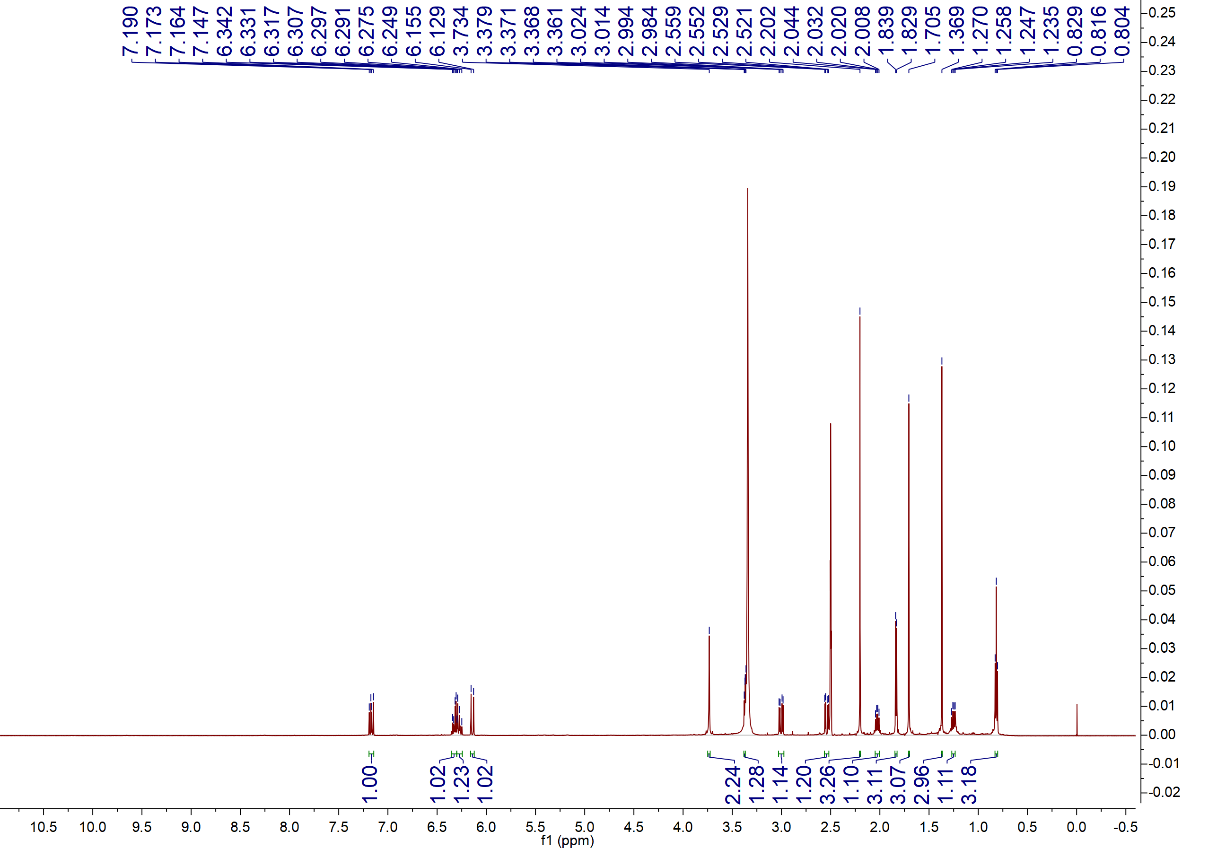


**Figure S17**. ^13^C NMR (150 MHz, DMSO-*d_6_*) spectrum of trichodermolide B (**3**)


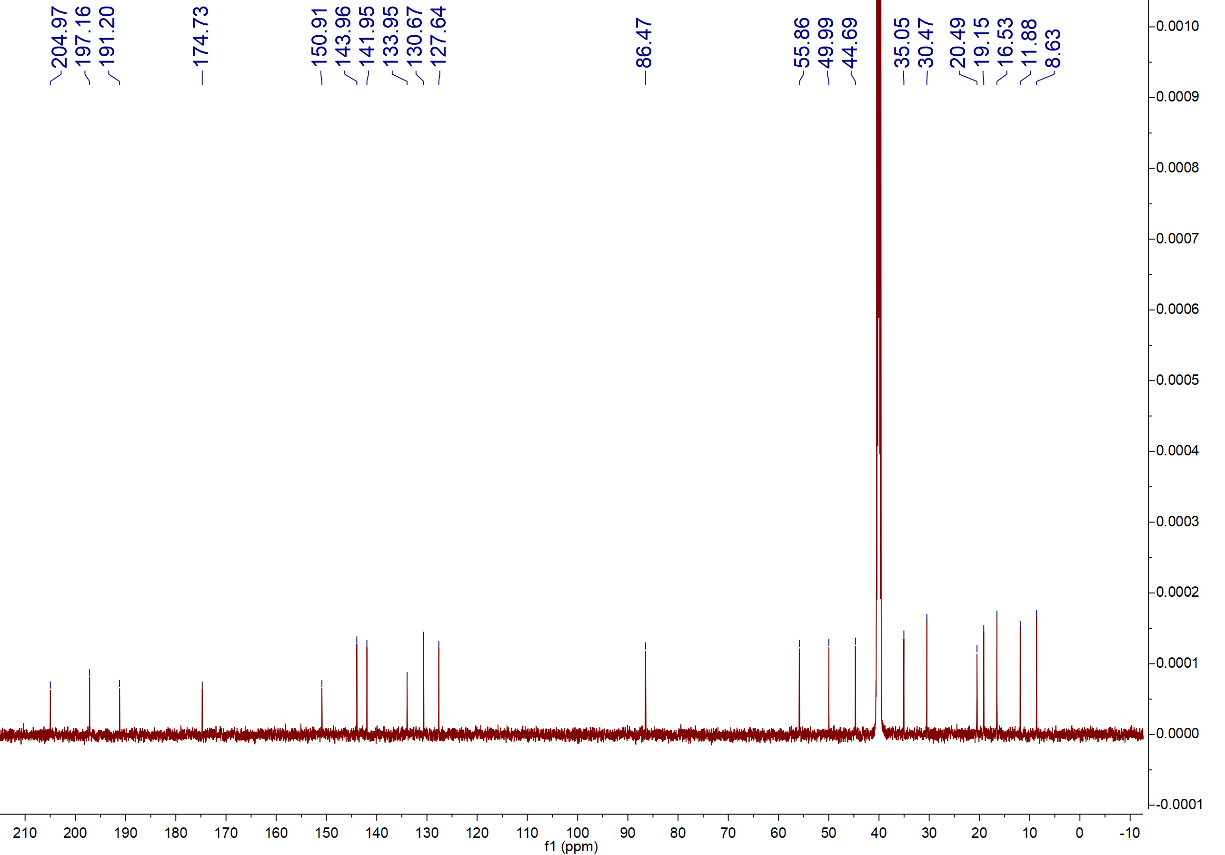


**Figure S18**. HSQC spectrum of trichodermolide B (**3**) in DMSO-*d_6_*


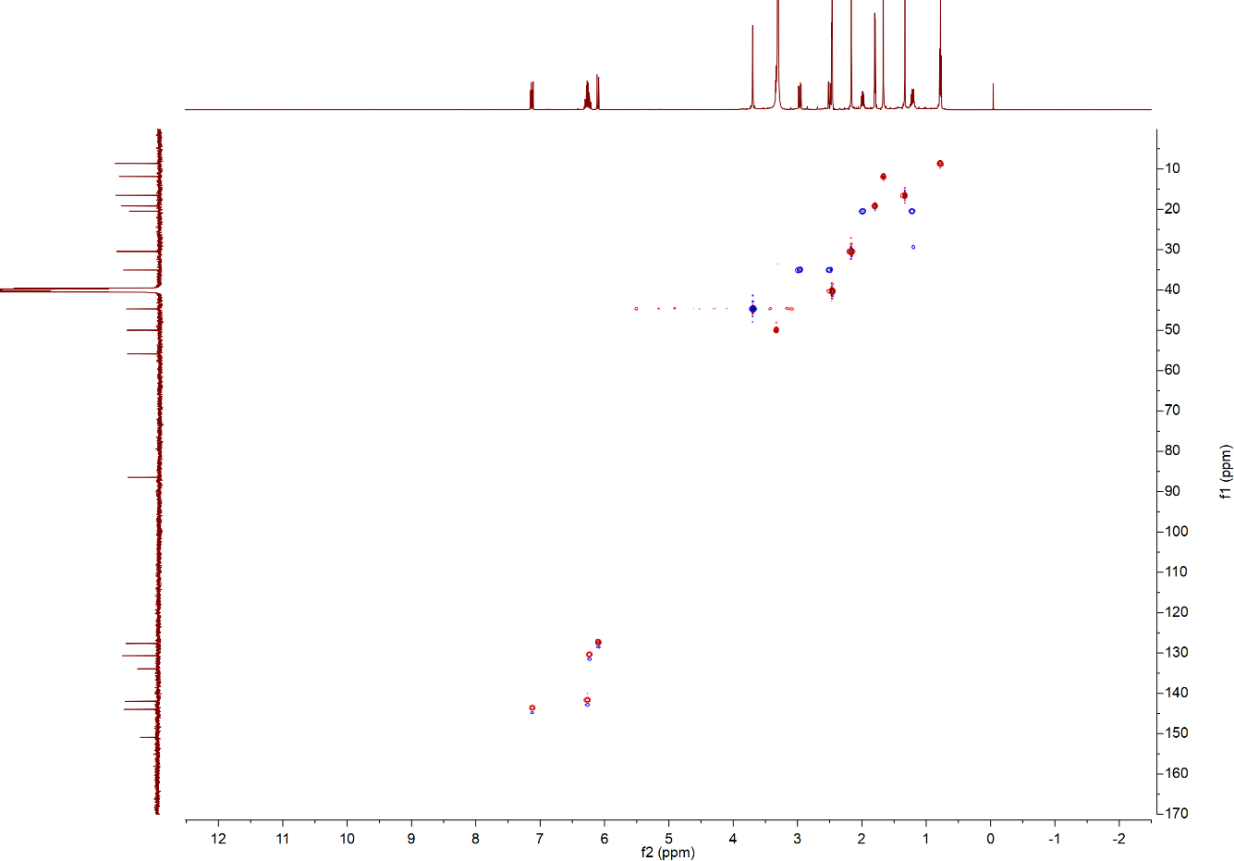


**Figure S19**. ^1^H-^1^H COSY spectrum of trichodermolide B (**3**) in DMSO-*d_6_*


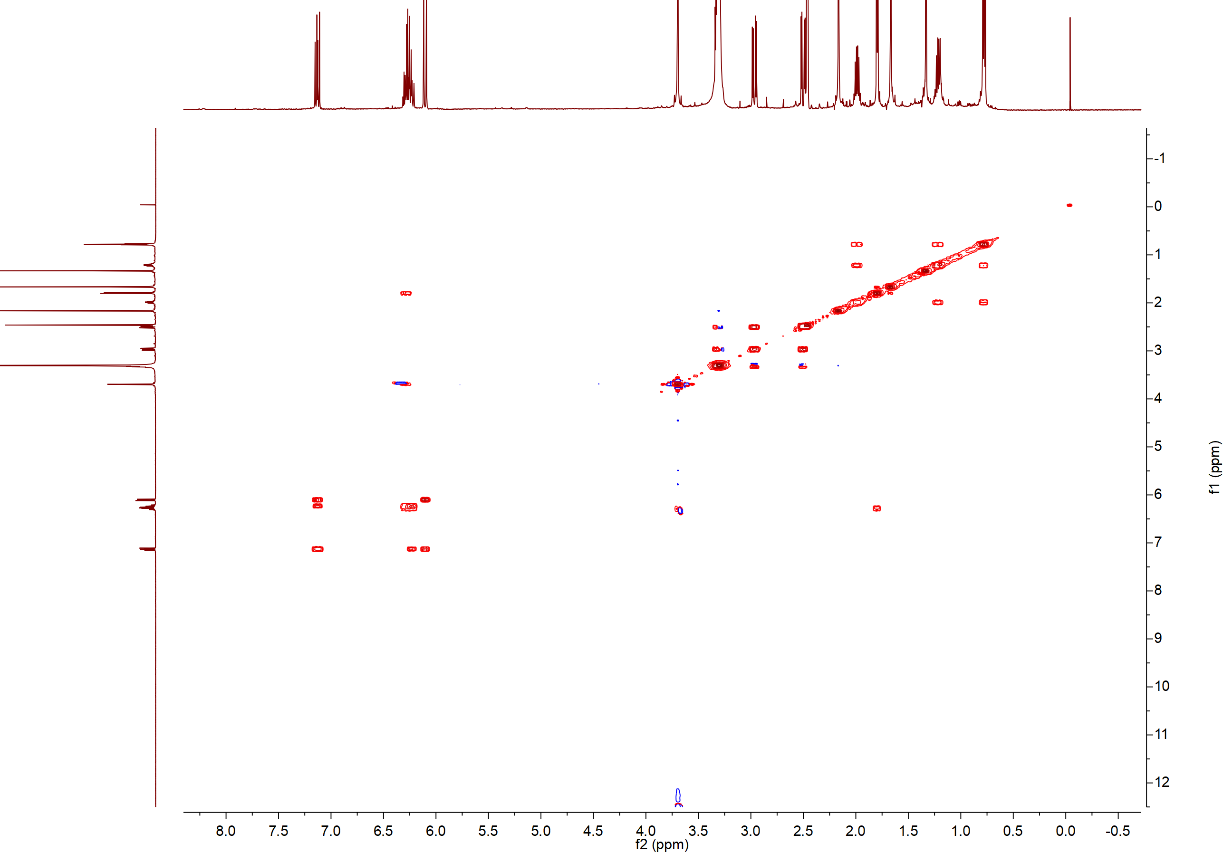


**Figure S20**. HMBC spectrum of trichodermolideB (**3)** inDMSO-*d_6_*


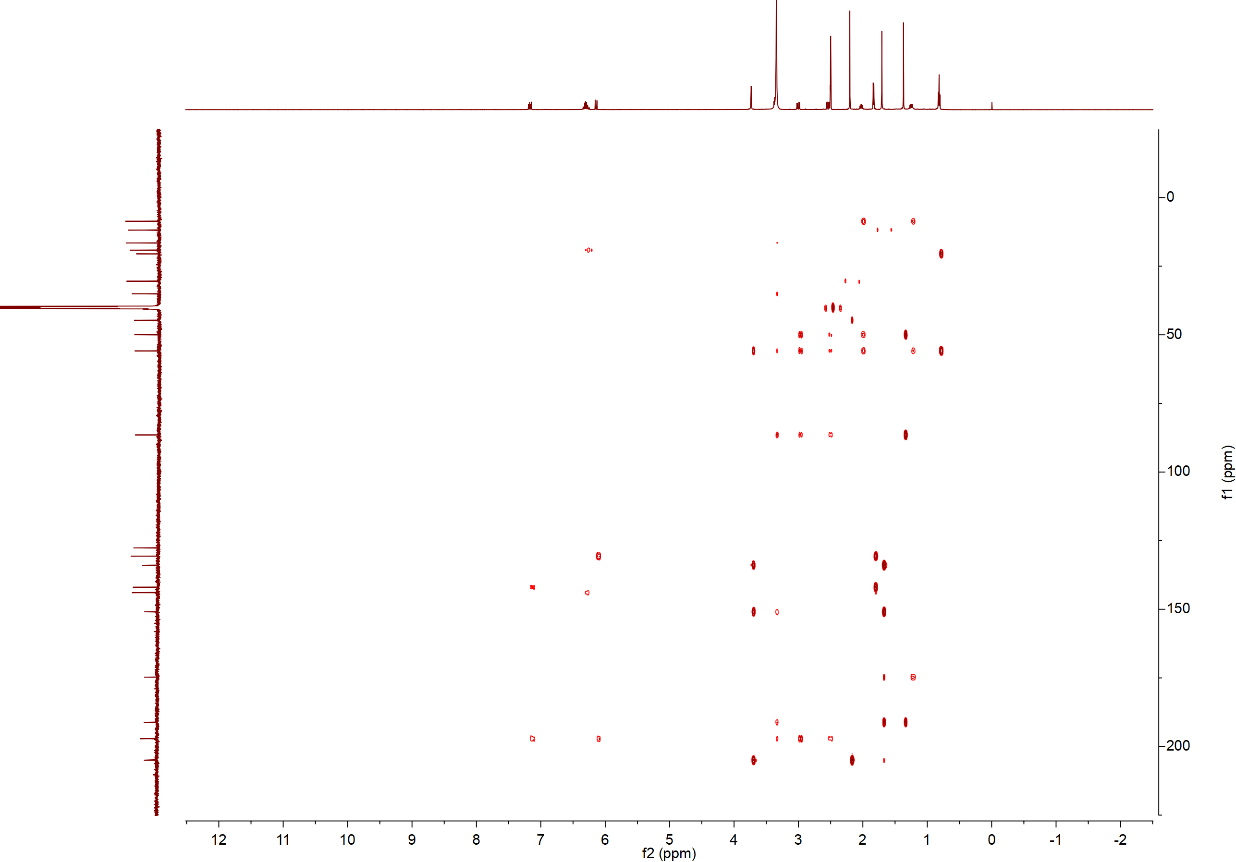


**Figure S21**. NOSEY spectrum of trichodermolideB(**3)** inDMSO-*d_6_*


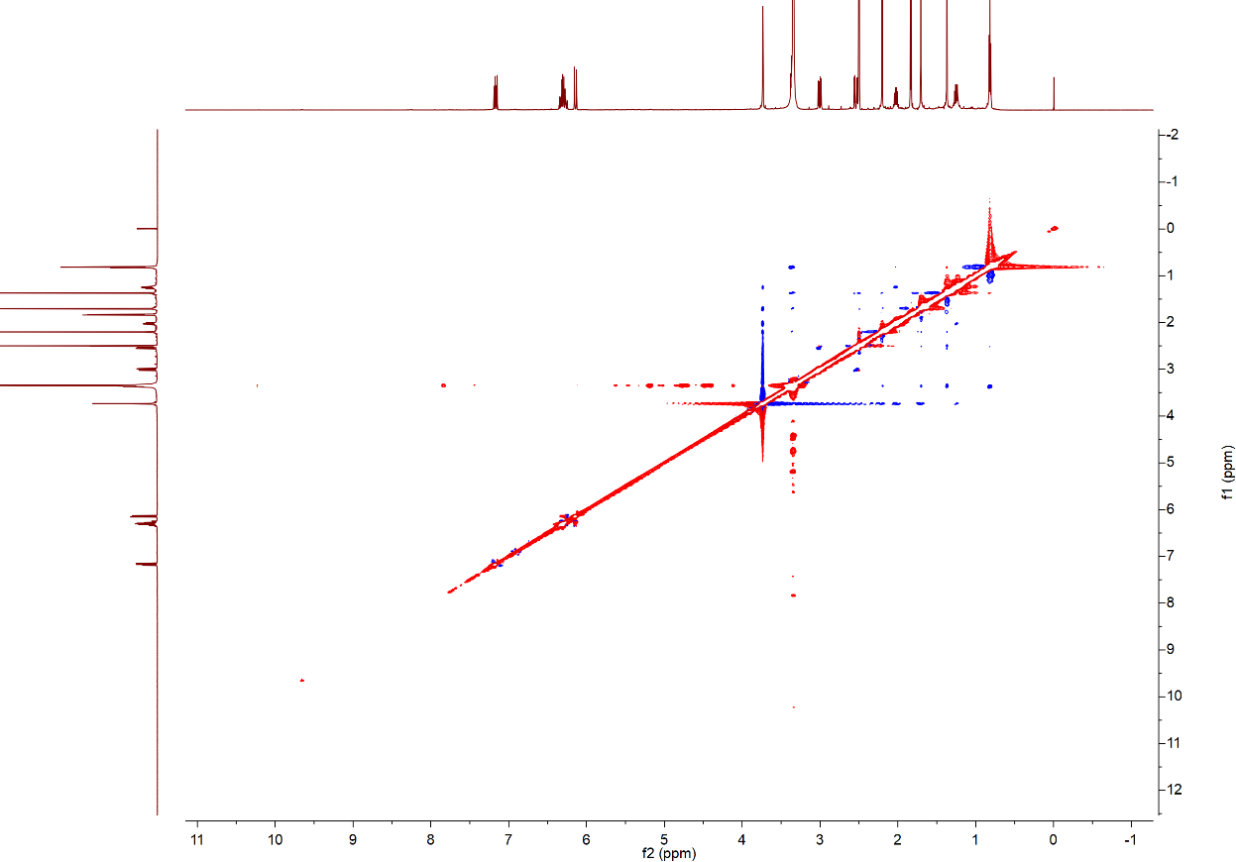


**Figure S22**. HR-ESIMS spectrum of trichodermolide B (**3**)

**Figure S23**. ^1^H NMR (600 MHz, CDCl_3_) spectrum of 13-hydroxy-trichodermolide (**4**)


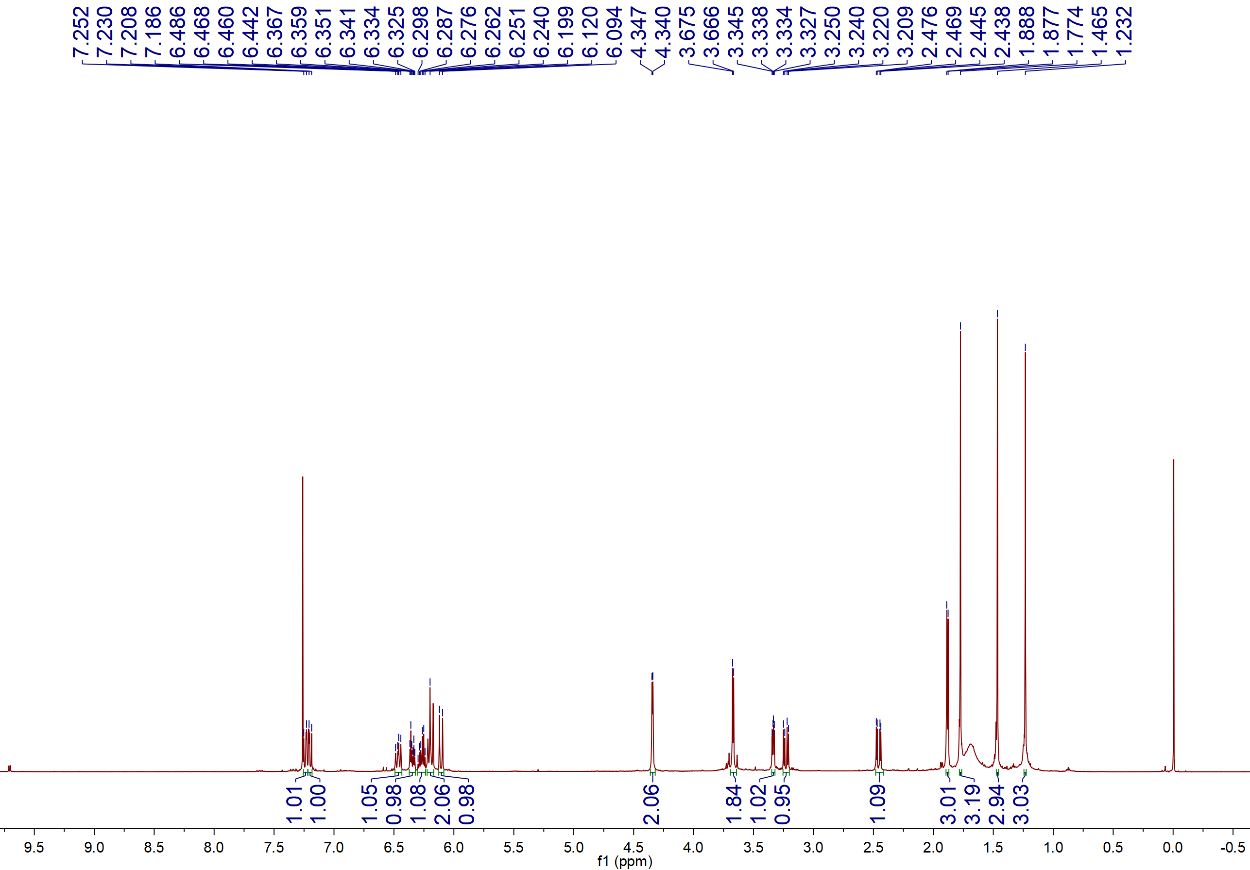


**Figure S24**. Partial enlarged drawing of the ^1^H NMR spectrum of 13-hydroxy-trichodermolide (**4**)


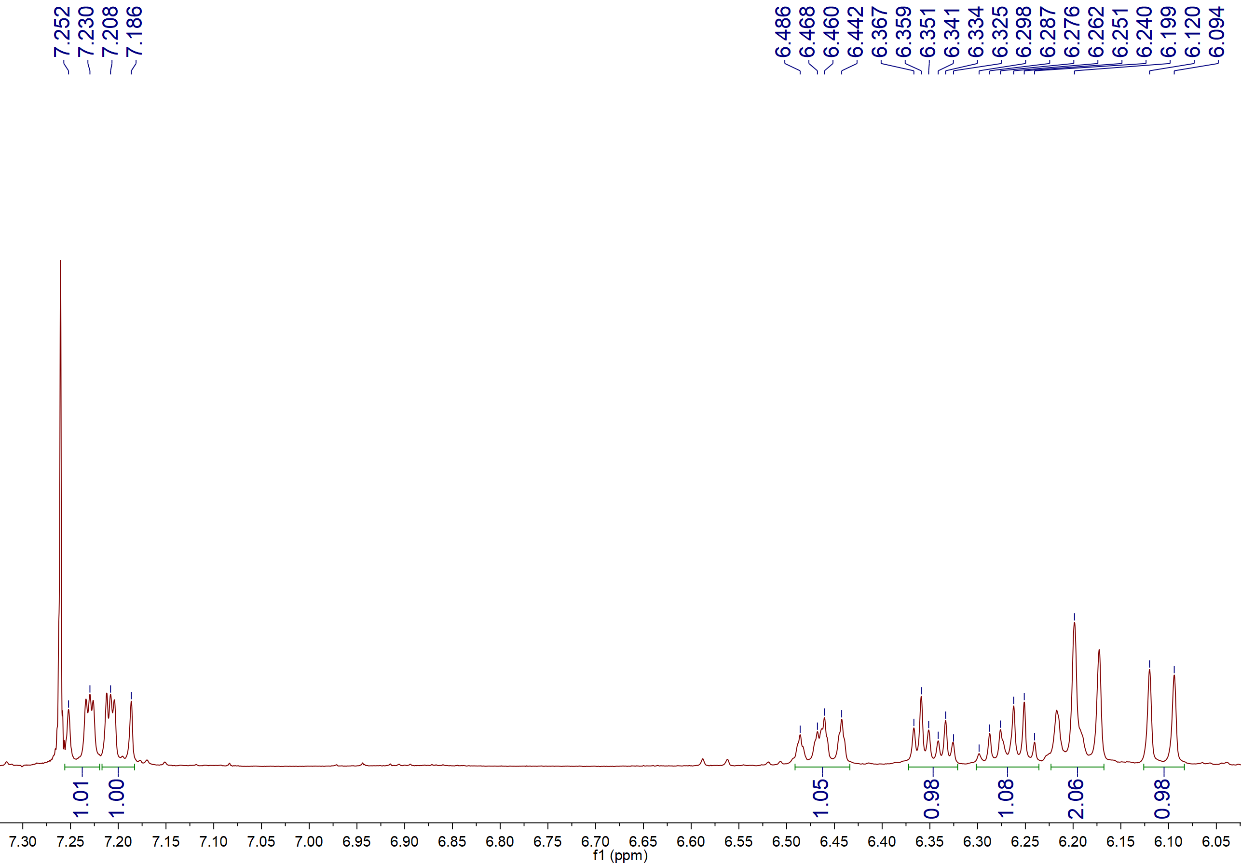


**Figure S25**. ^13^C NMR (150MHz, CDCl_3_) spectrum of 13-hydroxy-trichodermolide (**4**)


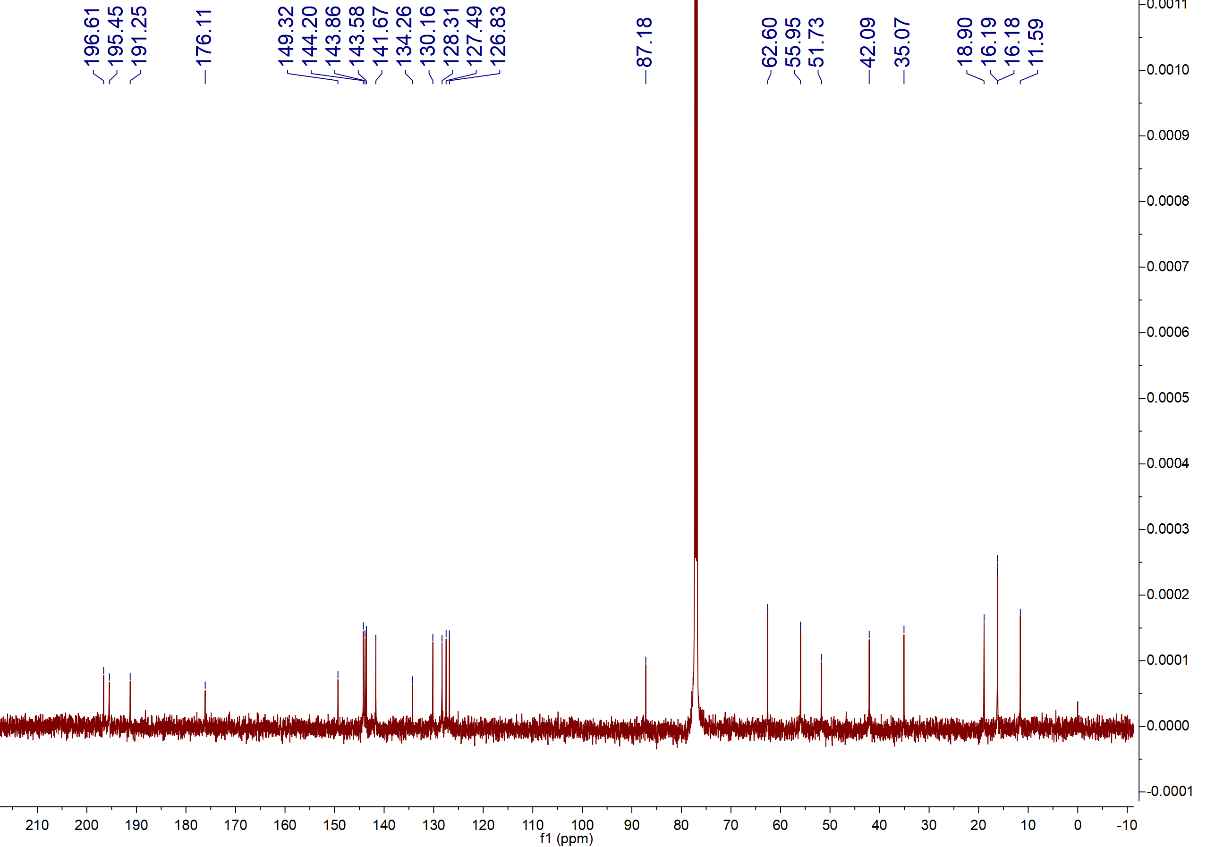


**Figure S26**. HSQC spectrum of 13-hydroxy-trichodermolide (**4**) in CDCl_3_


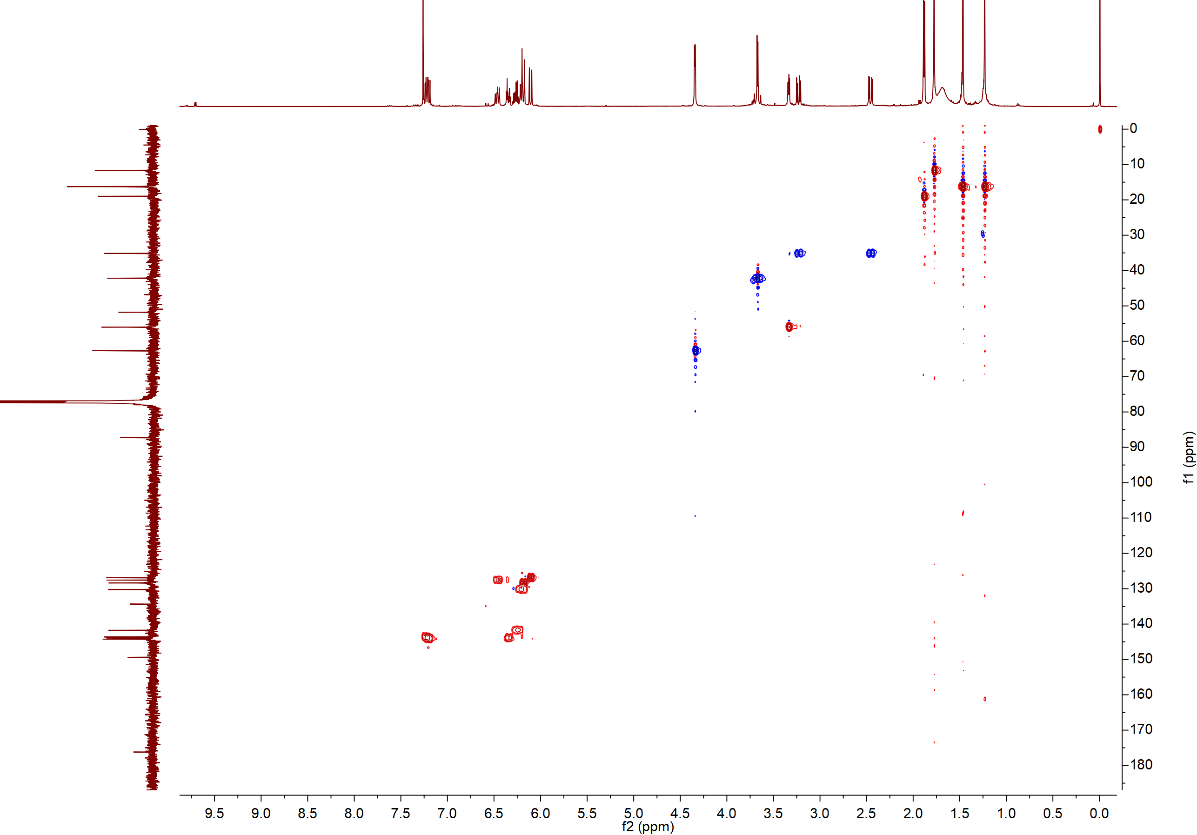


**Figure S27**. ^1^H-^1^H COSY spectrum of 13-hydroxy-trichodermolide (**4**) in CDCl_3_


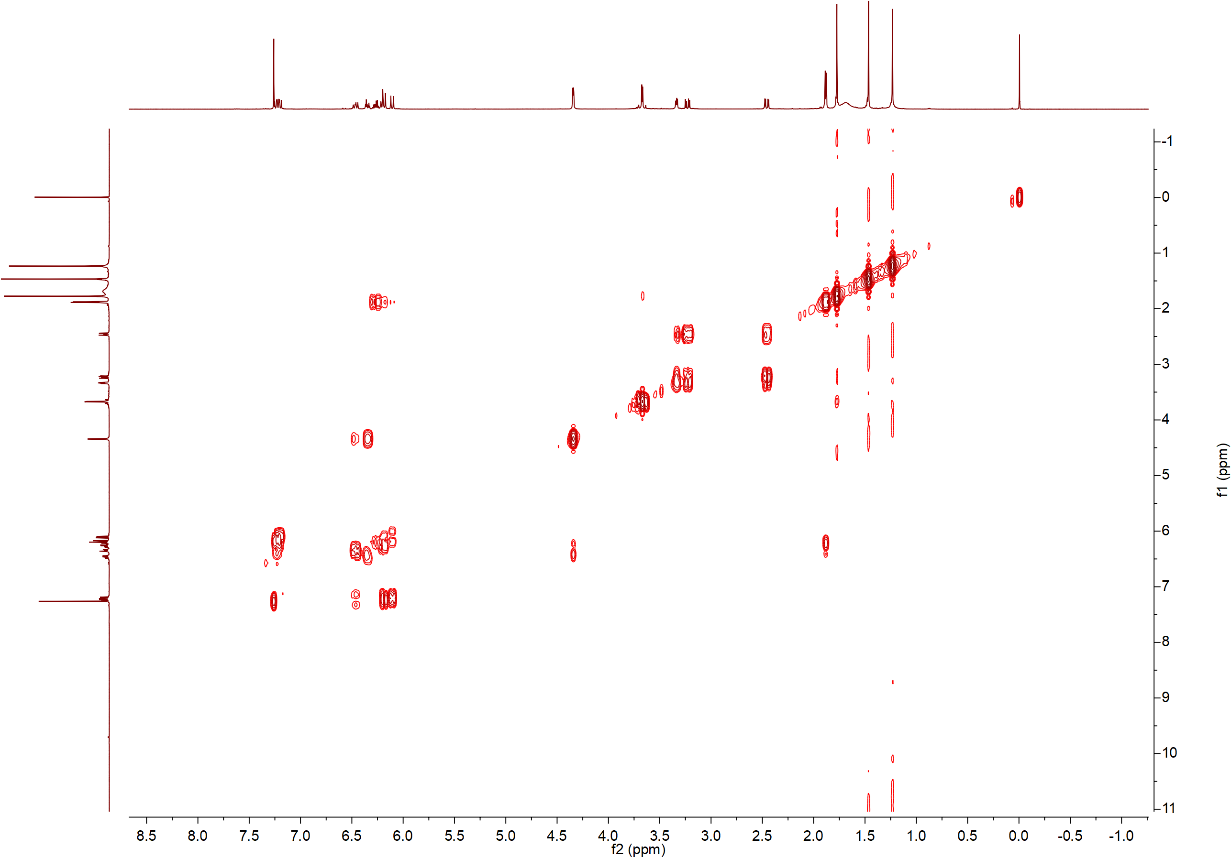


**Figure S28**. HMBC spectrum of 13-hydroxy-trichodermolide (**4**) in CDCl_3_


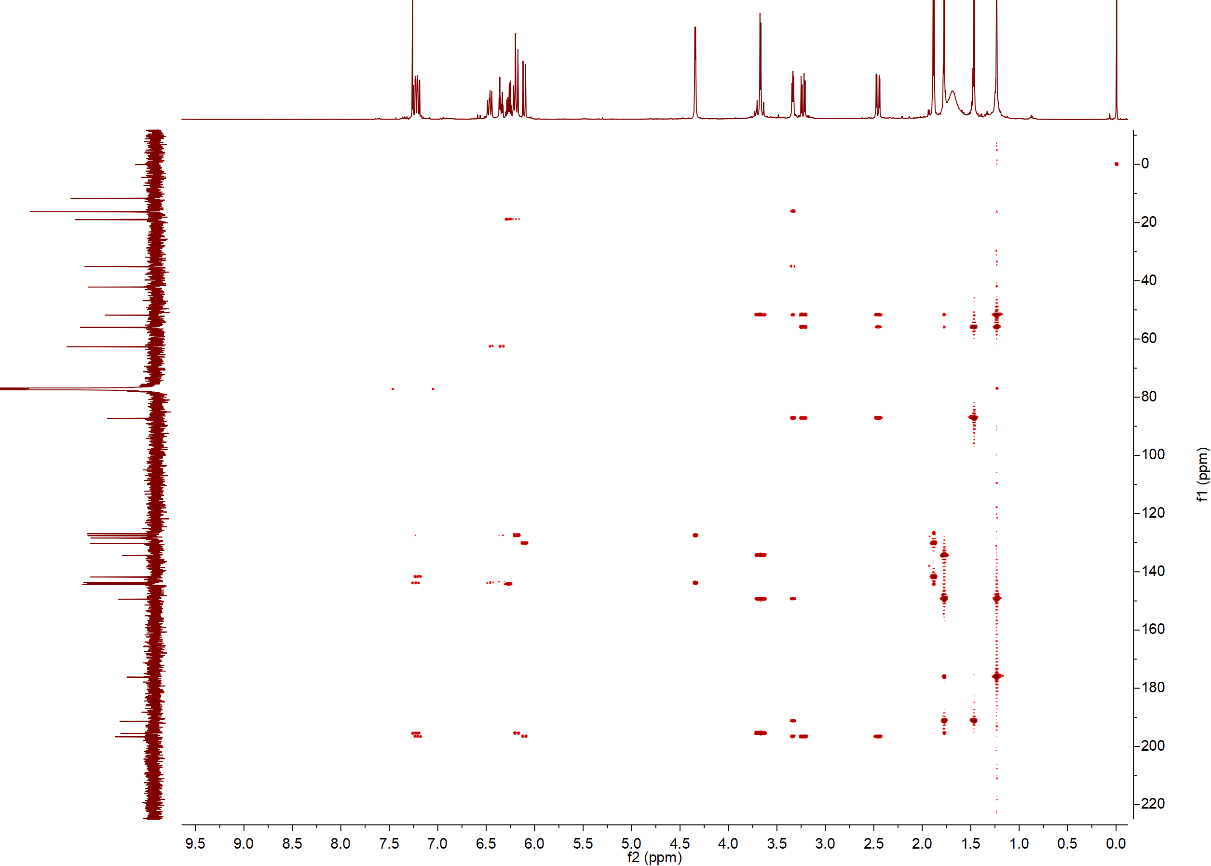


**Figure S29**. NOESY spectrum of 13-hydroxy-trichodermolide (**4**) in CDCl_3_


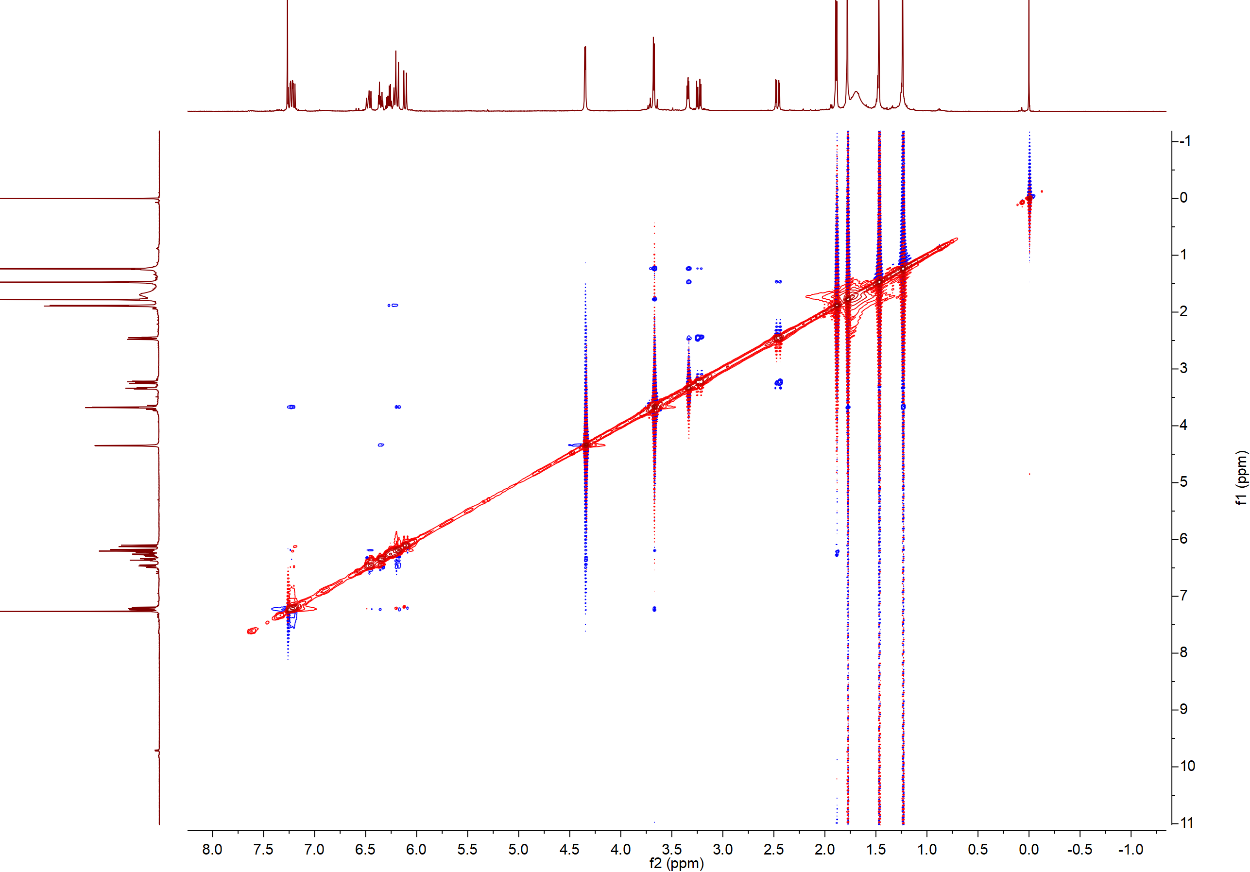


**Figure S30**. HR-ESIMS spectrum of 13-hydroxy-trichodermolide (**4**).

**Figure S31**.^1^NMR (600 MHz, CD_3_OD) spectrum of 24-hydroxy-trichodimerol (**5**)


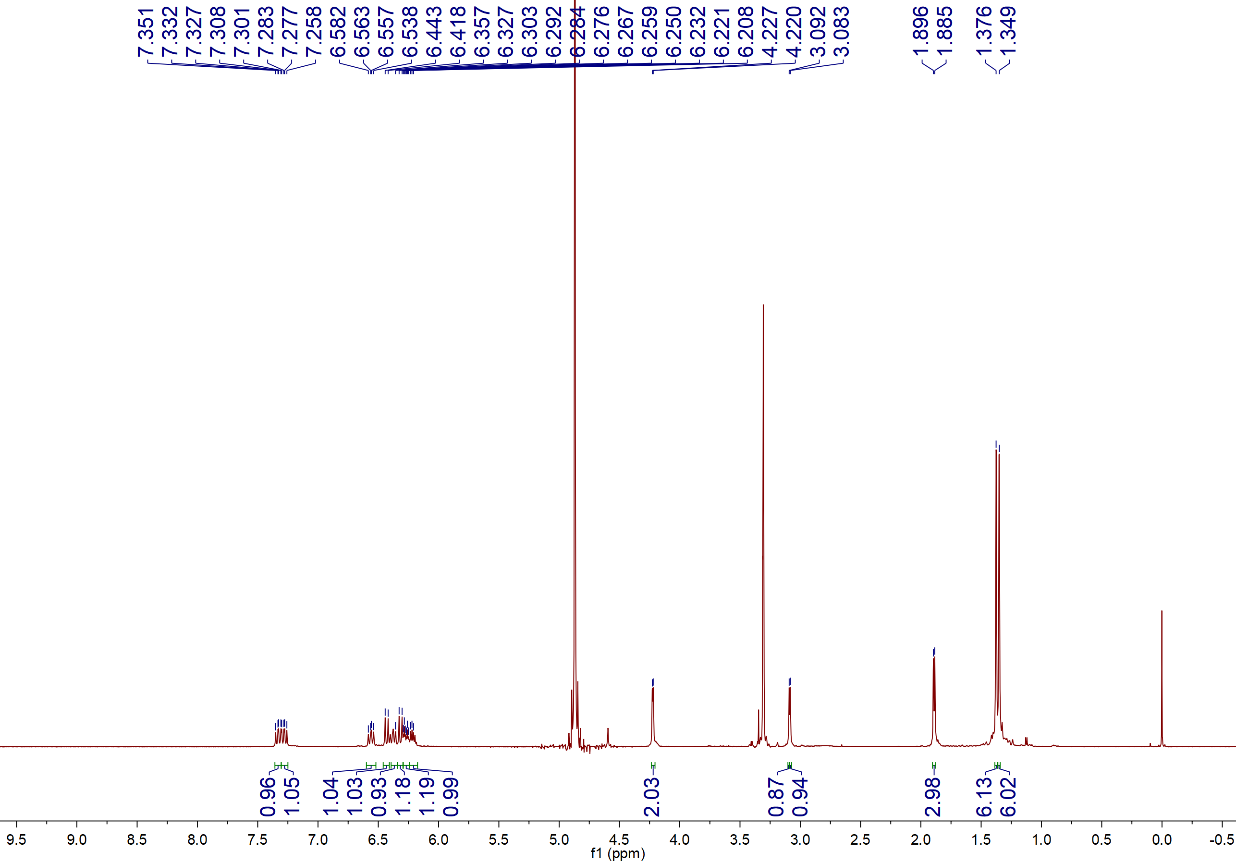


**Figure S32.** Partial enlarged drawing of the ^1^H NMR spectrum of 24-hydroxy-trichodimerol (**5**)


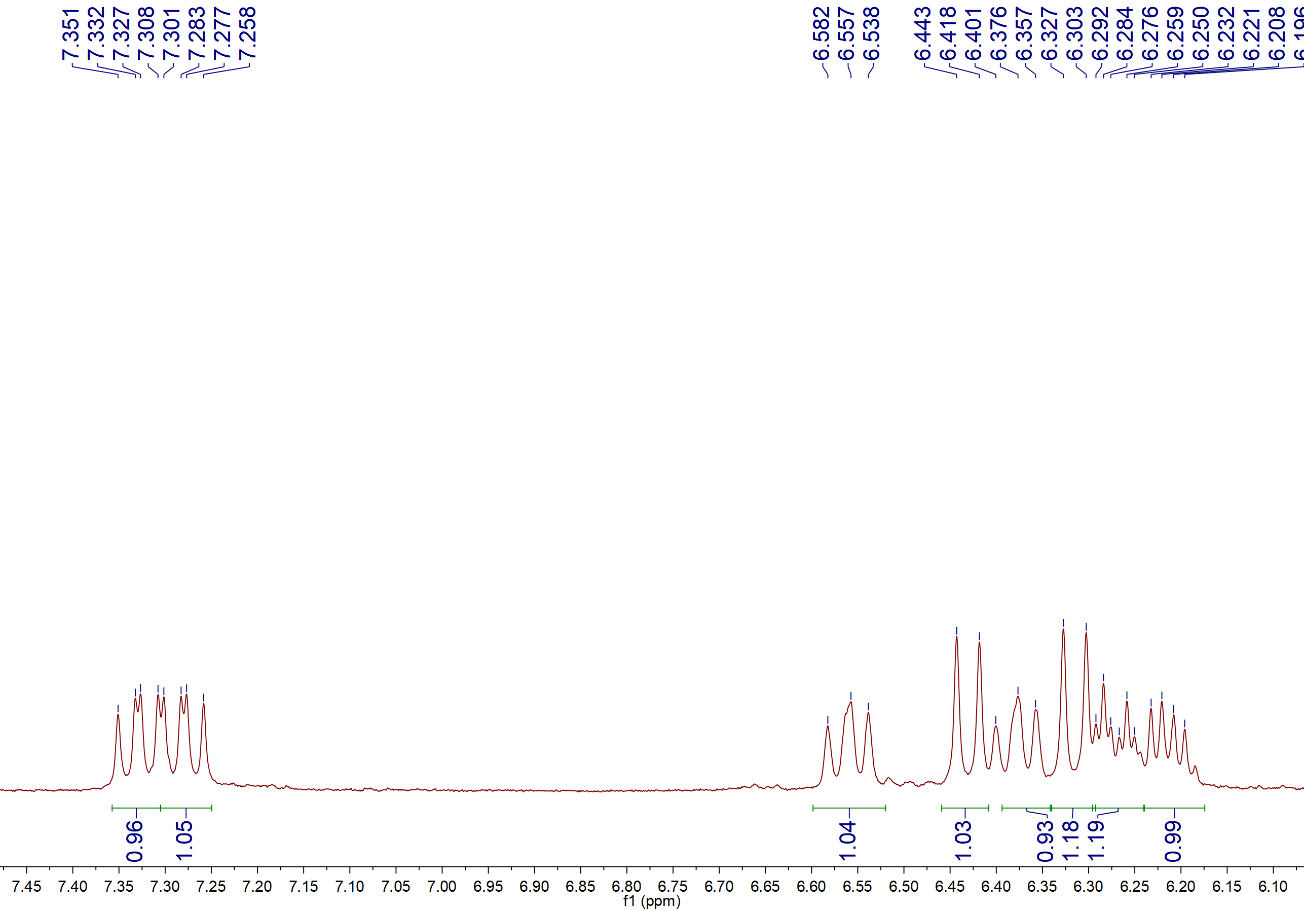


**Figure S33**. ^13^C NMR (150 MHz, CD_3_OD) spectrum of 24-hydroxy-trichodimerol (**5**)


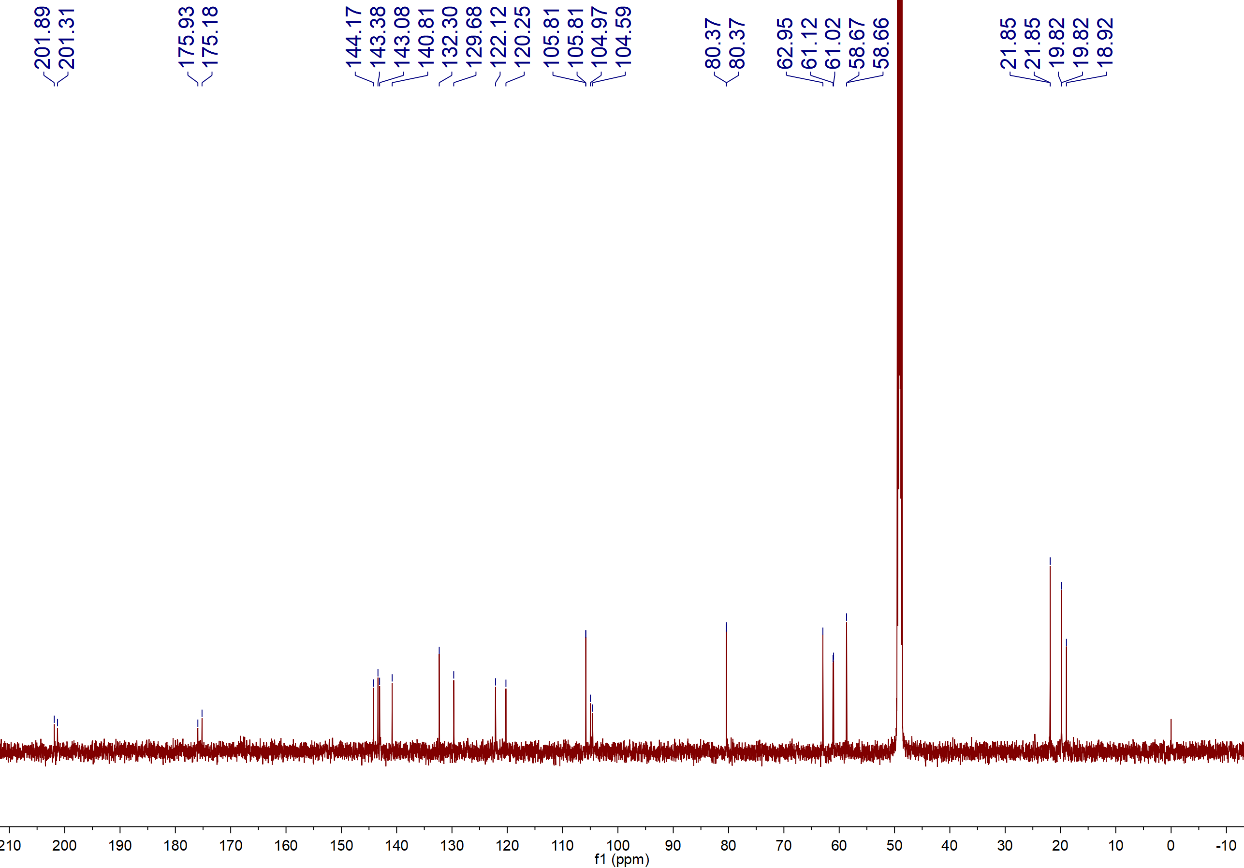


**Figure S34**. HSQC spectrum of 24-hydroxy-trichodimerol (**5**) in CD_3_OD


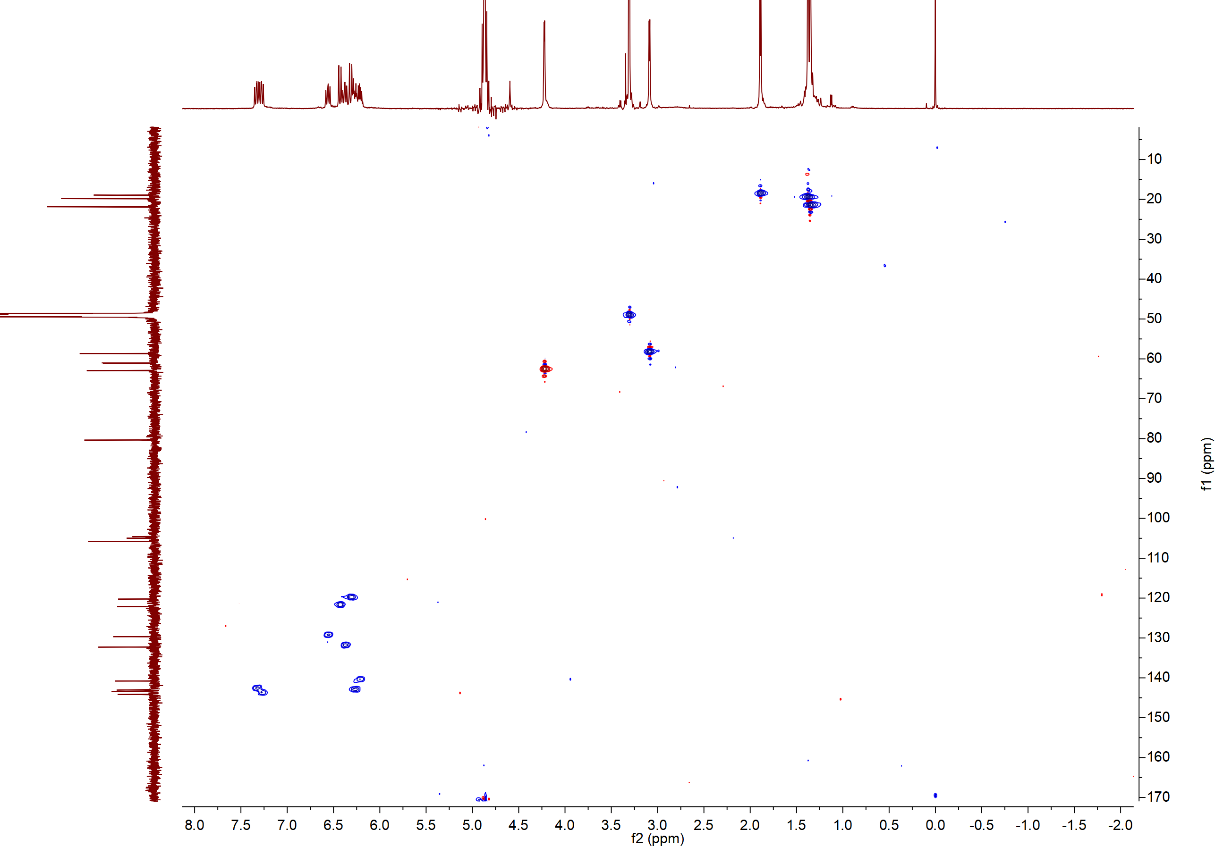


**Figure S35**. ^1^H-^1^H COSY spectrum of 24-hydroxy-trichodimerol (**5**) in CD_3_OD


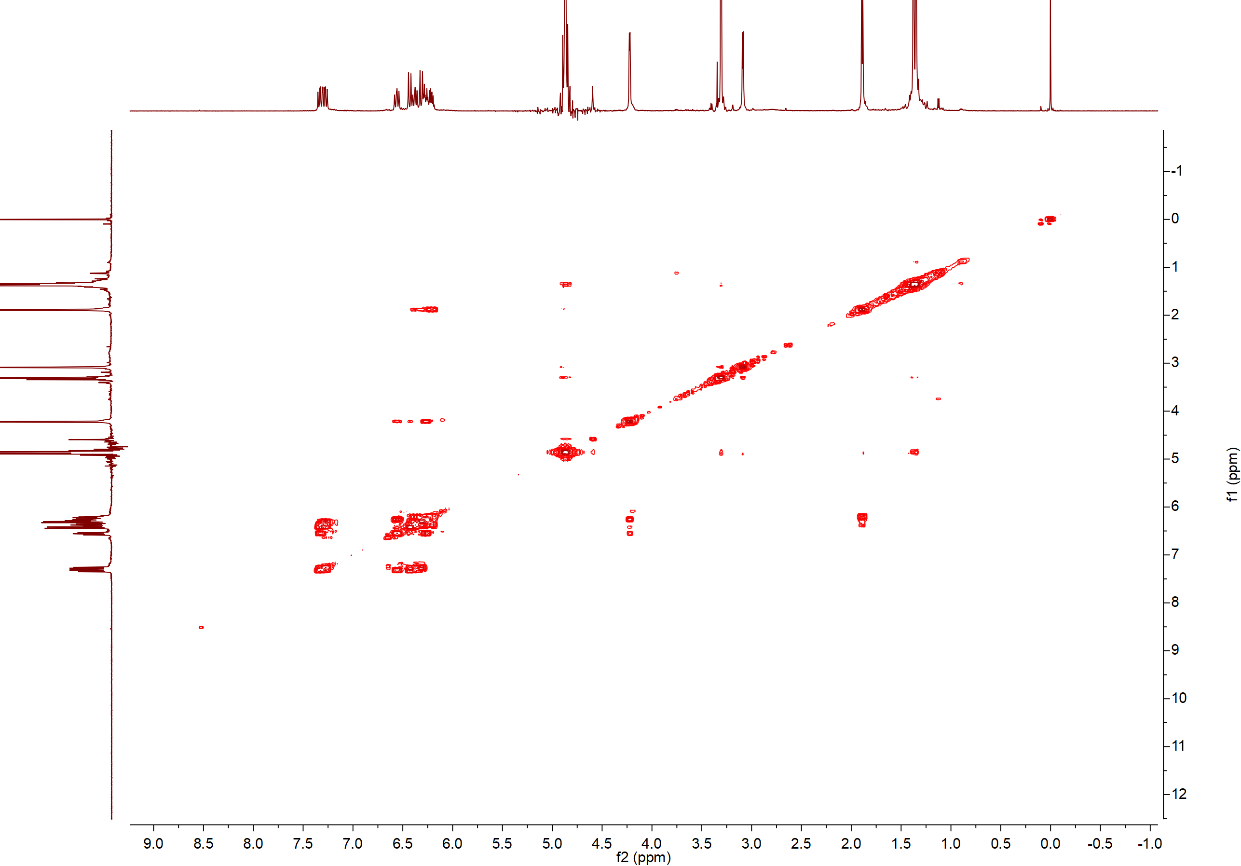


**Figure S36**. HMBC spectrum of 24-hydroxy-trichodimerol (**5**) in CD_3_OD


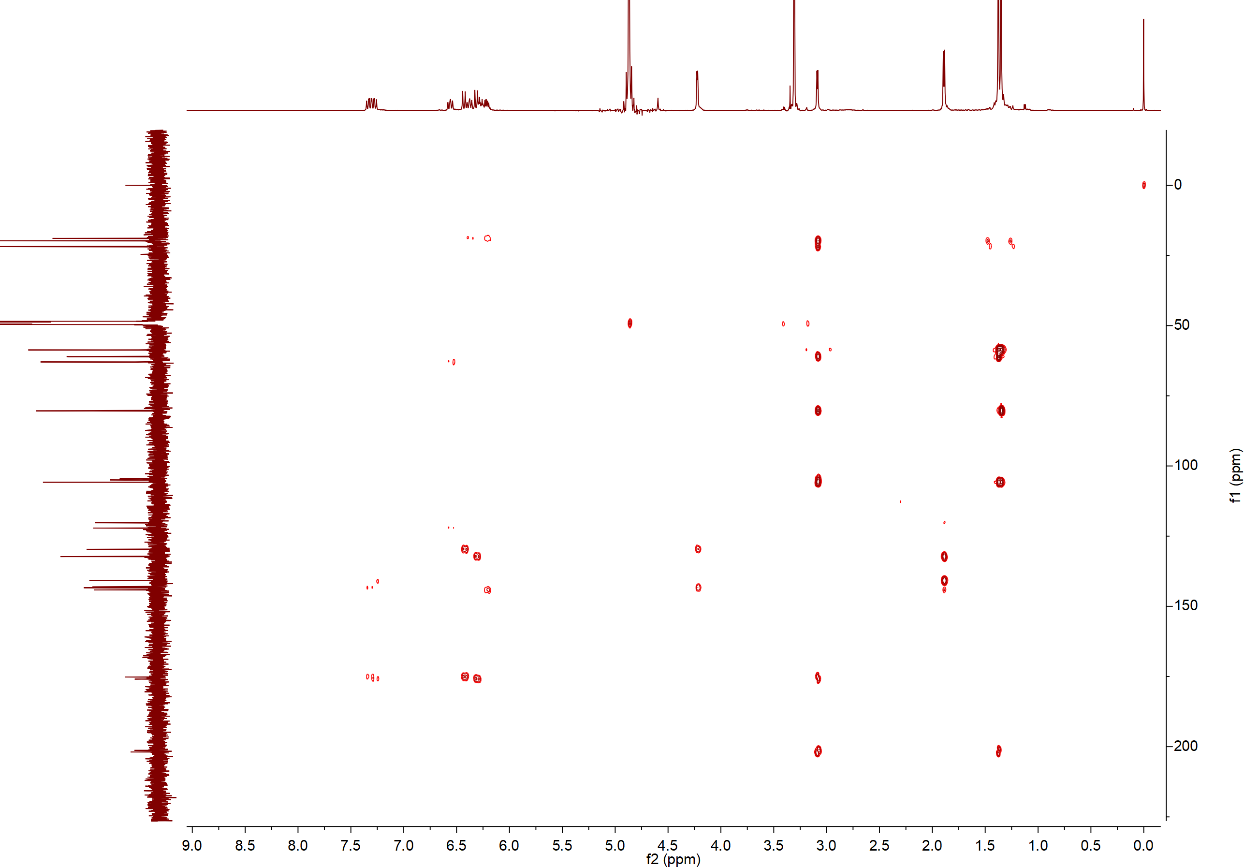


**Figure S37**. NOESY spectrum of 24-hydroxy-trichodimerol (**5**) in CD_3_OD


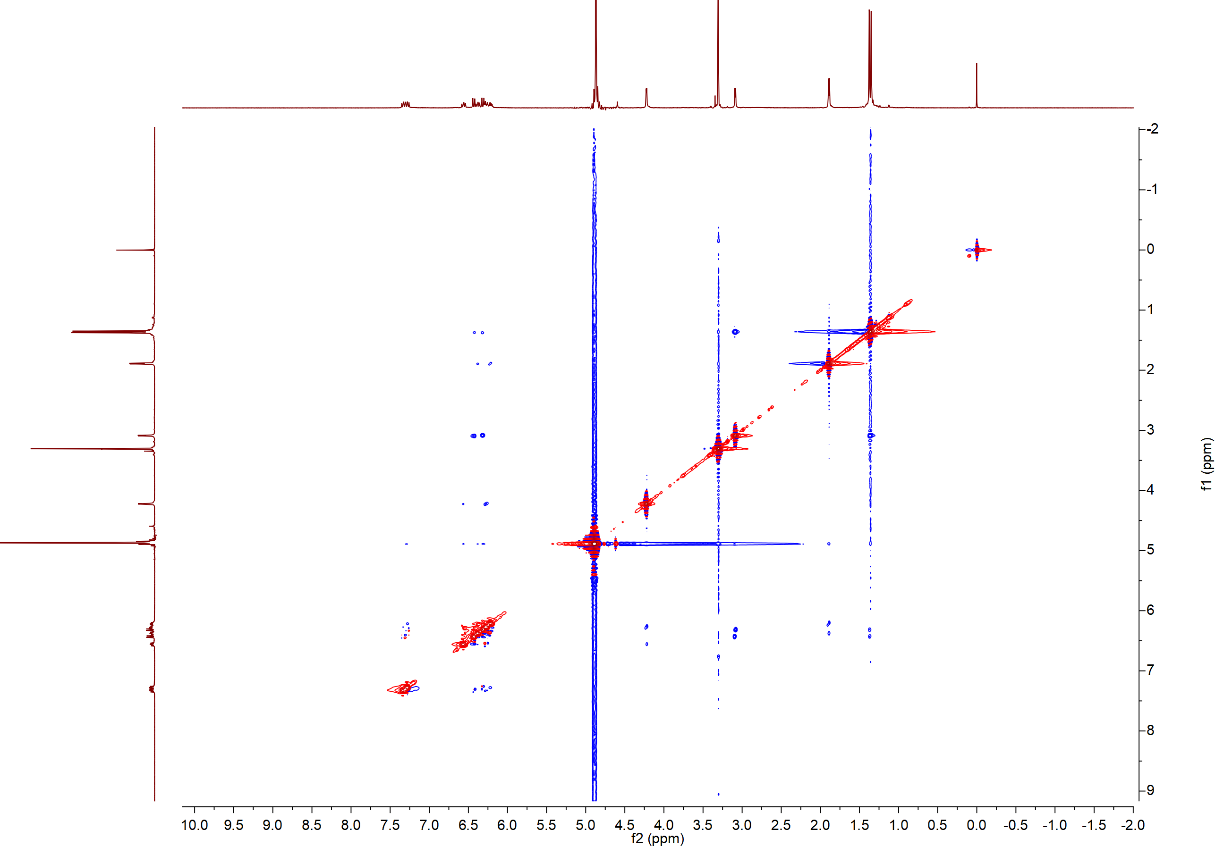


**Figure S38**. HR-ESIMS spectrum of 24-hydroxy-trichodimerol (**5**)

**Figure S39**. ^1^HNMR (400 MHz, CD_3_OD) spectrum of 15-hydroxy-bisvertinol (**7**)


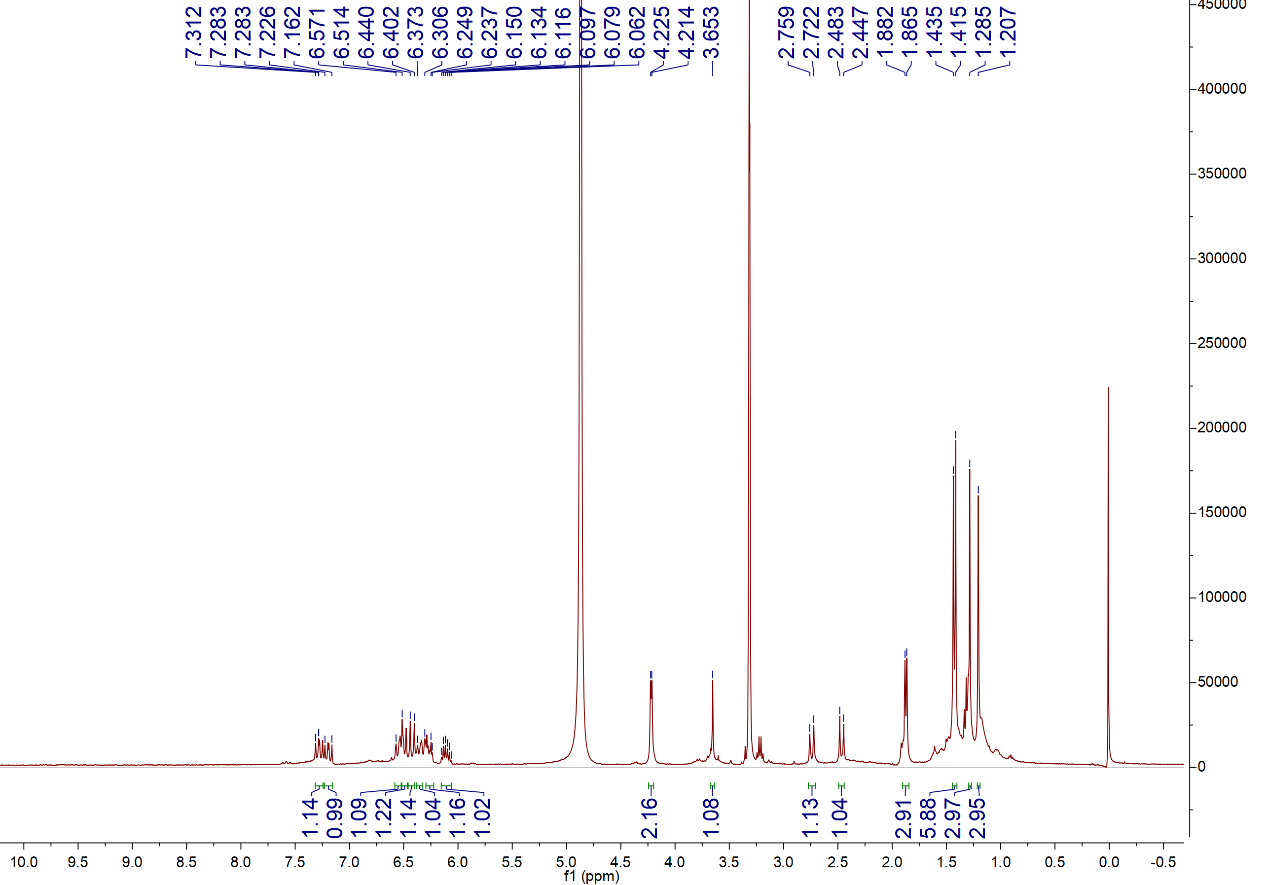


**Figure S40**. Partial enlarged drawing of the ^1^H NMR spectrum of 15-hydroxy-bisvertinol (**7**)


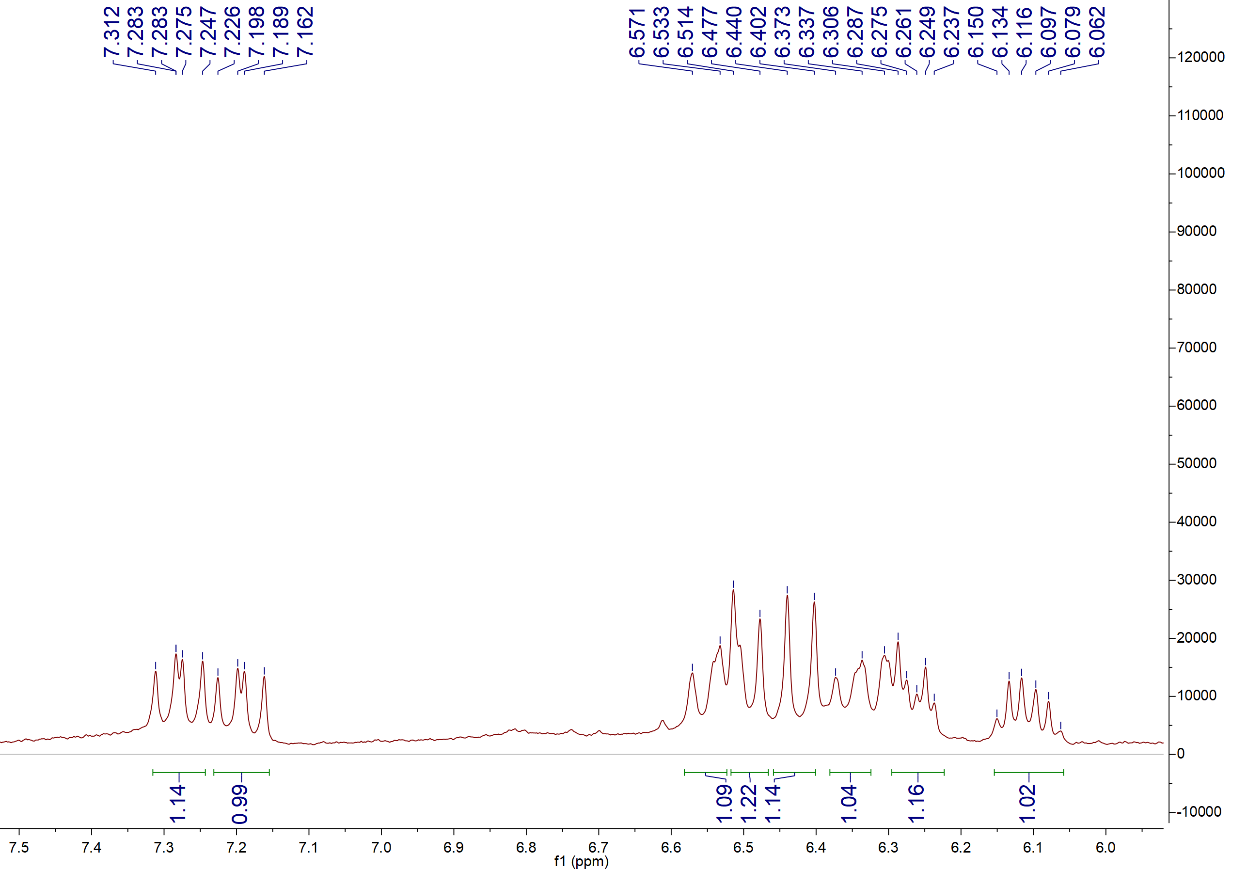


**Figure S41**. ^13^C NMR (100 MHz, CD_3_OD) spectrum of 15-hydroxy-bisvertinol (**7**)


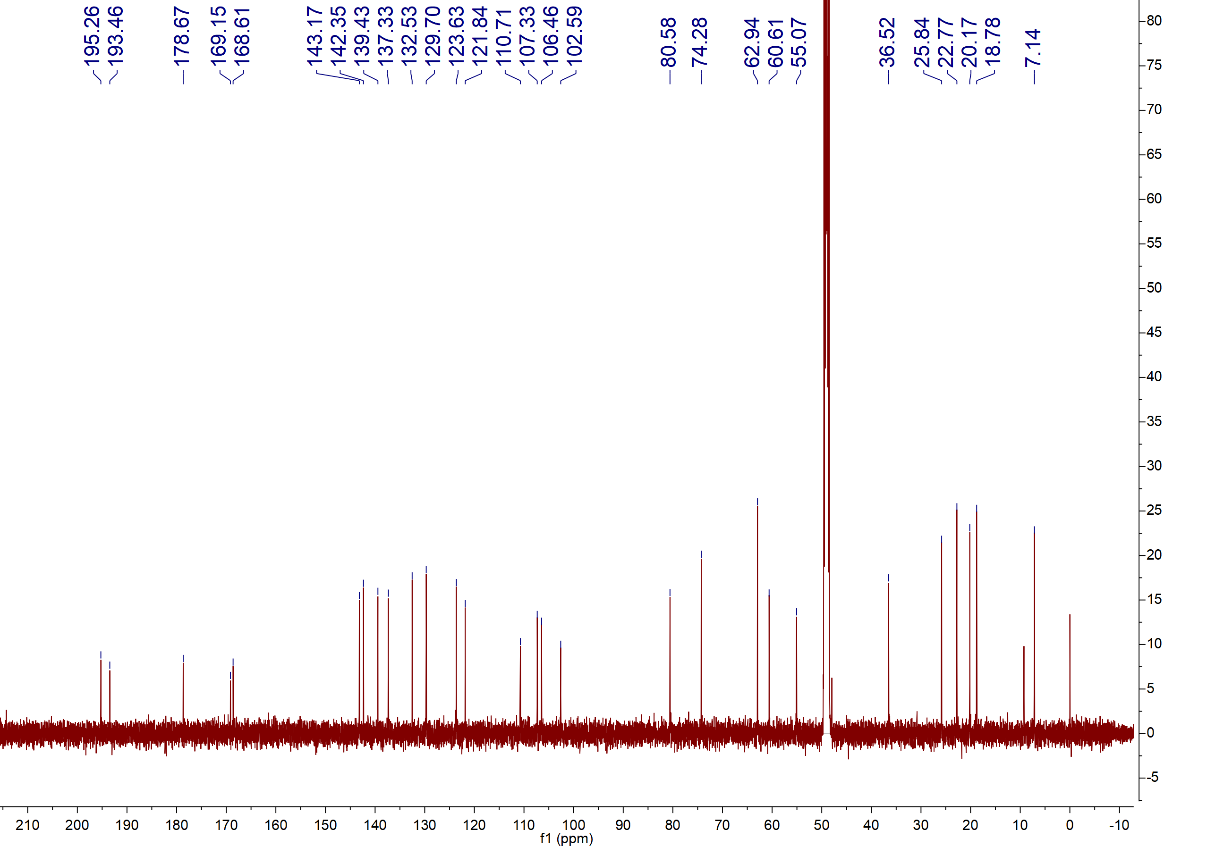


**Figure S42**. HSQC spectrum of 15-hydroxy-bisvertinol (**7**) in CD_3_OD


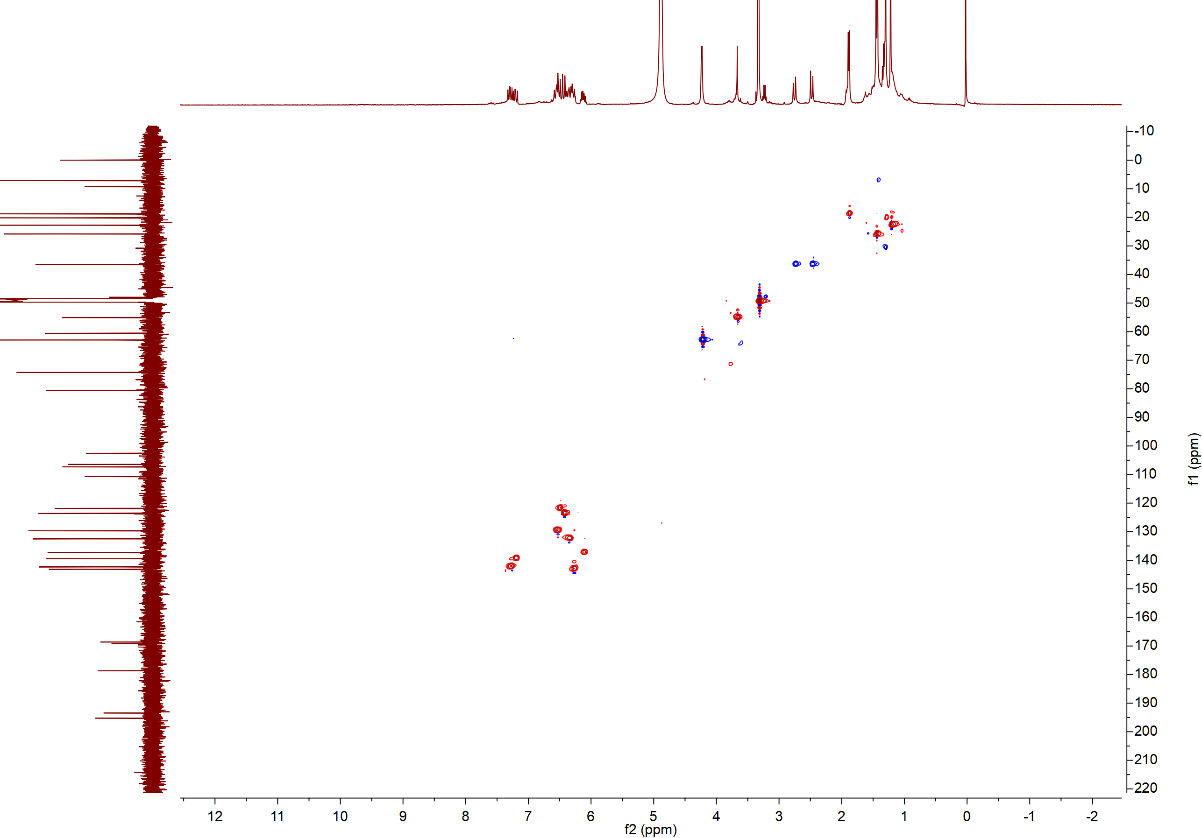


**Figure S43**.H-^1^H COSY spectrum of 15-hydroxy-bisvertinol (**7**) in CD_3_OD


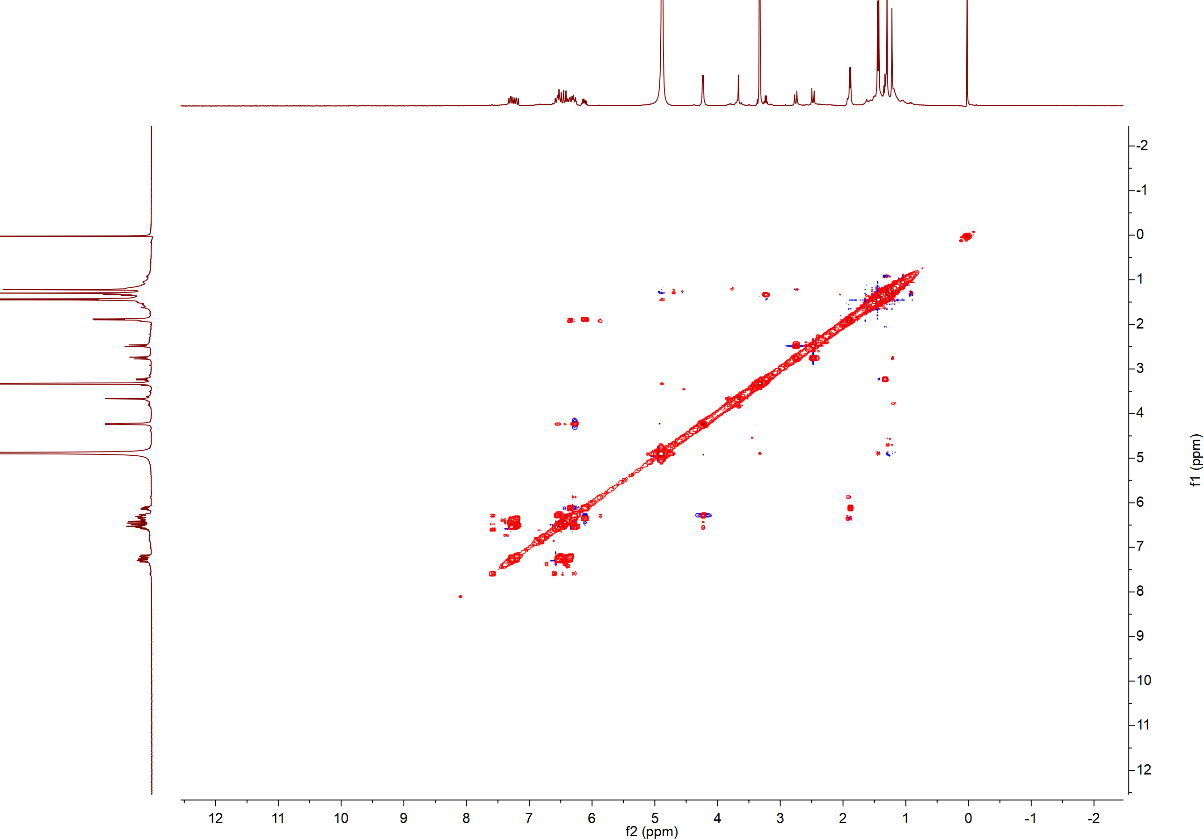


**Figure S44**. HMBC spectrum of 15-hydroxy-bisvertinol (**7**) in CD_3_OD


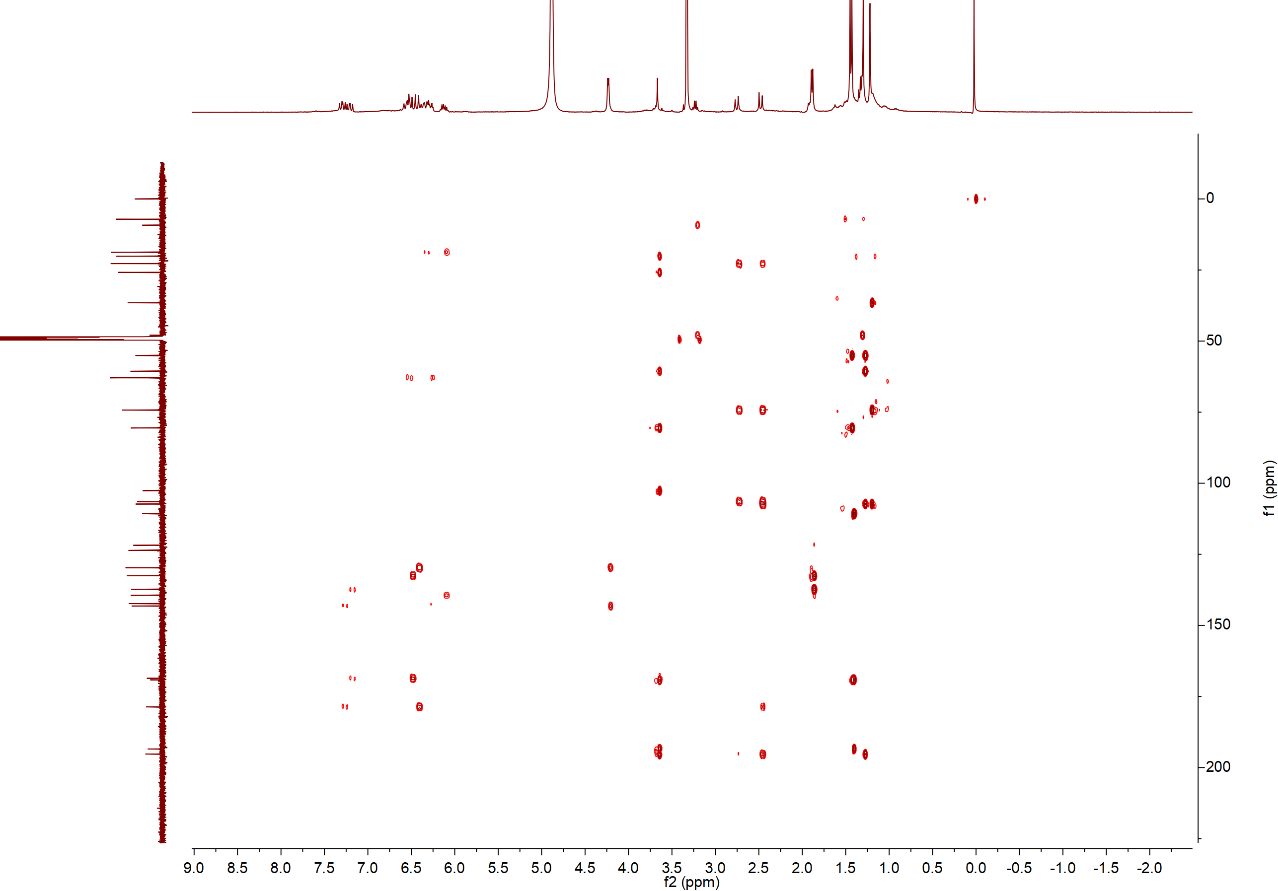


**Figure S45**. NOESY spectrum of 15-hydroxy-bisvertinol (**7**) in CD_3_OD


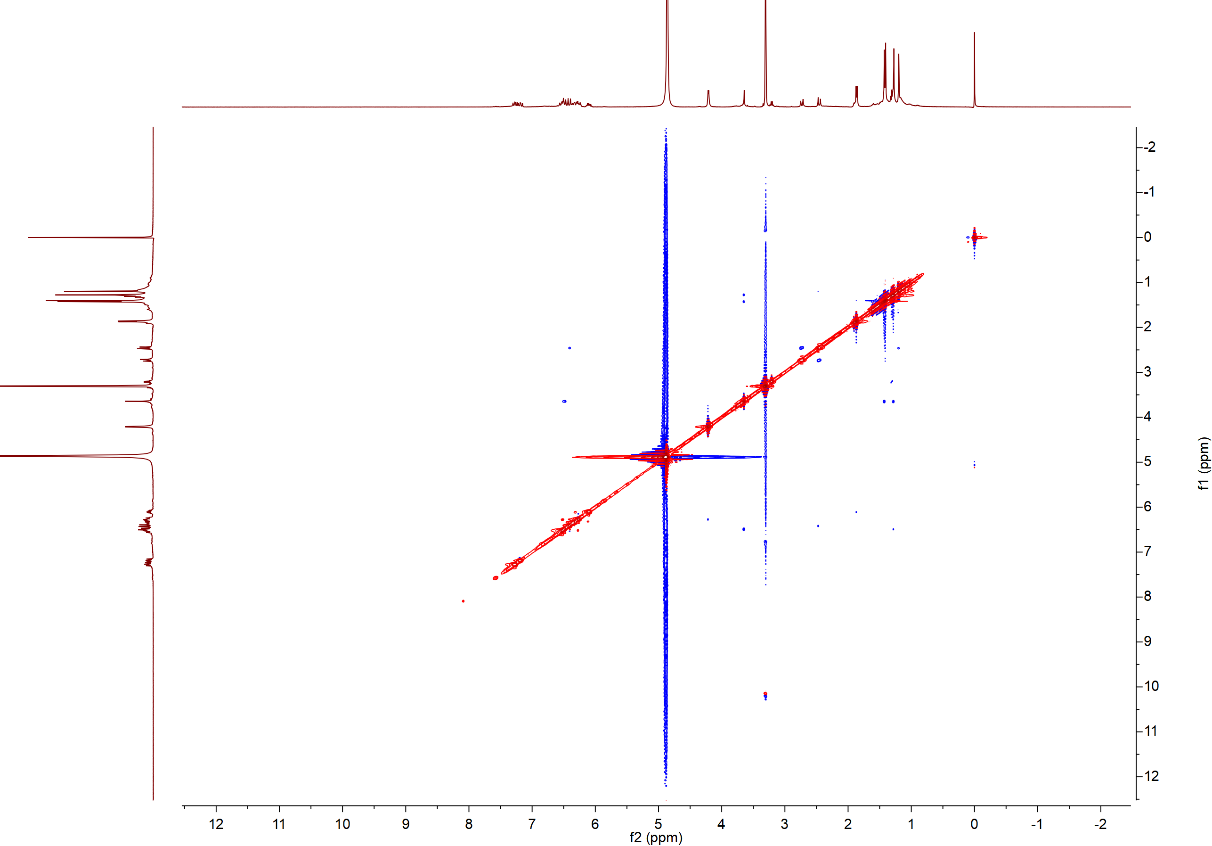


**Figure S46**. HR-ESIMS spectrum of 15-hydroxy-bisvertinol (**7**)

**Figure S47** Structures and population of the low-energy B3LYP/6-311+G(d) conformers (>5%)

of (8*R*,9*S*,10*S*)-**1**.


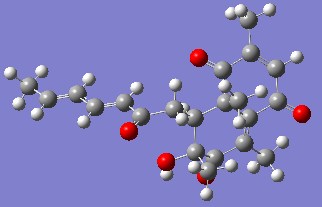

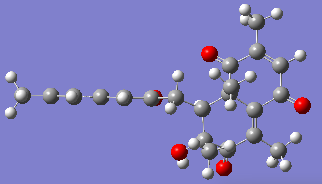

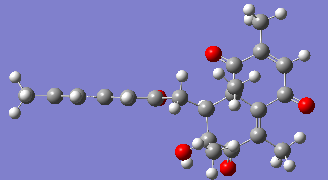


Conf. A 29.77% Conf. B 20.35% Conf. C 20.34%


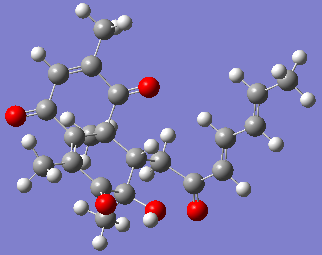

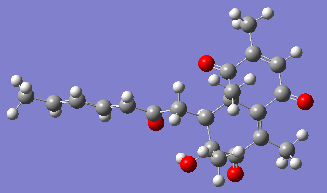

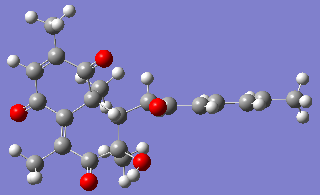
 Conf. D 10.55% Conf. E 9.41% Conf. F 5.10%

**Figure S48** Structures and population of the low-energy B3LYP/6-311+G(d) conformers (>5%)

of (8*S*,9*R*,10*R*)-**1**.


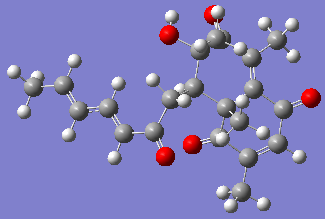

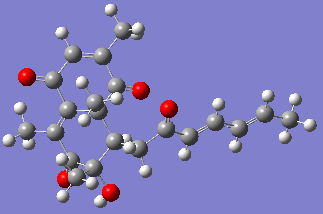

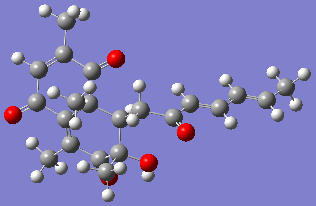


Conf. A 51.41% Conf. B 35.58% Conf. C 5.15%

**Figure S49**. Structures and population of the low-energy B3LYP/6-311+G(d) conformers (>5%)

of (4*S*,5*R*,6*R*)-**3**.


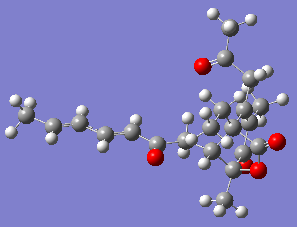

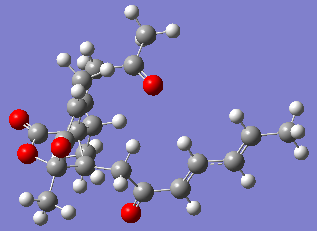


Conf. A 64.68% Conf. B 33.01%

**Figure S50**. The neighbor-joining phylogenetic tree of fungal strain *T. reesei* (HN-2016-018)


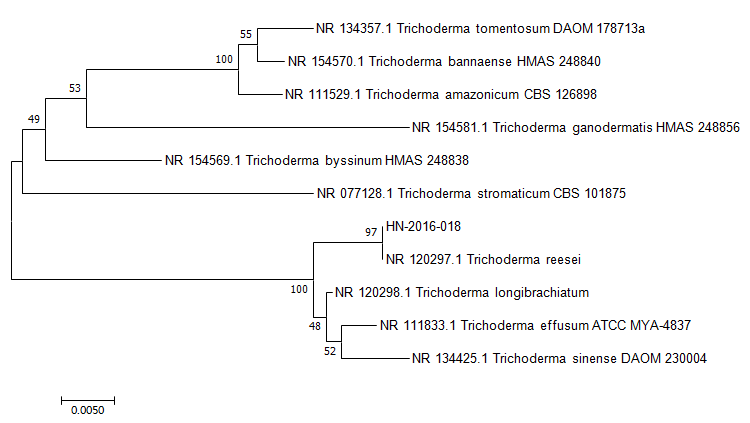


**Table S1**. Cartesian coordinates of the low-energy conformers (≥5%) of (8*R*,9*S*,10*S*)-**1**

Standard orientation of Conf. A

---------------------------------------------------------------------

Center Atomic Atomic Coordinates (Angstroms)

Number Number Type X Y Z

---------------------------------------------------------------------

1 6 0 0.193904 -1.829308 0.312255

2 6 0 1.050010 -2.102031 -0.923199

3 6 0 2.372413 -1.426977 -1.028290

4 6 0 2.578616 -0.293558 -0.318615

5 6 0 1.540610 0.386793 0.578582

6 6 0 0.133059 -0.293288 0.528734

7 6 0 3.342134 -2.067885 -1.984102

8 6 0 2.116908 0.479256 2.028105

9 6 0 -0.766528 0.097635 1.723640

10 8 0 -1.129800 -2.263195 0.052467

11 8 0 0.649202 -2.874312 -1.776737

12 6 0 -2.257817 -0.227772 1.608362

13 8 0 -2.805298 -0.892757 2.470952

14 6 0 -2.998031 0.396410 0.488947

15 6 0 -4.316300 0.189657 0.308998

16 6 0 -5.108677 0.775907 -0.748974

17 6 0 -6.422633 0.545079 -0.908824

18 6 0 -7.278869 1.127053 -1.987563

19 6 0 3.879245 0.449730 -0.440630

20 6 0 3.800391 1.924821 -0.464661

21 6 0 2.664674 2.607526 -0.235143

22 6 0 1.406254 1.853293 0.077747

23 8 0 0.331358 2.404212 -0.033343

24 8 0 4.957152 -0.112724 -0.537764

25 6 0 2.558813 4.099454 -0.310859

26 6 0 0.753471 -2.666737 1.480252

27 1 0 -0.349462 0.084146 -0.377055

28 1 0 2.790228 -2.500444 -2.819256

29 1 0 4.096371 -1.377636 -2.353278

30 1 0 3.874091 -2.887760 -1.490300

31 1 0 3.129892 0.882093 2.033273

32 1 0 1.501715 1.124669 2.656012

33 1 0 2.149990 -0.505400 2.491504

34 1 0 -0.421438 -0.357539 2.651929

35 1 0 -0.707562 1.183499 1.848953

36 1 0 -1.103255 -2.820828 -0.742353

37 1 0 -2.446416 1.047356 -0.184276

38 1 0 -4.821765 -0.470923 1.011039

39 1 0 -4.596026 1.438036 -1.446445

40 1 0 -6.919947 -0.119871 -0.202935

41 1 0 -6.710181 1.780608 -2.653115

42 1 0 -7.737655 0.338433 -2.595277

43 1 0 -8.105992 1.708709 -1.564135

44 1 0 4.733068 2.431686 -0.696647

45 1 0 3.514367 4.552752 -0.576973

46 1 0 1.807021 4.391262 -1.048657

47 1 0 2.224604 4.516425 0.644155

48 1 0 0.143910 -2.513339 2.371317

49 1 0 0.687764 -3.724746 1.216679

50 1 0 1.795877 -2.441328 1.712657

---------------------------------------------------------------------

Standard orientation of Conf. B

---------------------------------------------------------------------

Center Atomic Atomic Coordinates (Angstroms)

Number Number Type X Y Z

---------------------------------------------------------------------

1 6 0 0.335839 -1.805904 0.311074

2 6 0 1.471233 -2.257207 -0.599067

3 6 0 2.780589 -1.558354 -0.446957

4 6 0 2.798334 -0.317944 0.095339

5 6 0 1.560076 0.473390 0.525934

6 6 0 0.238129 -0.260137 0.139182

7 6 0 3.967427 -2.314708 -0.979712

8 6 0 1.661779 0.807392 2.049021

9 6 0 -1.053226 0.281304 0.772218

10 8 0 -0.885396 -2.380161 -0.119514

11 8 0 1.297255 -3.150830 -1.407878

12 6 0 -2.251994 0.252903 -0.182428

13 8 0 -2.113272 0.270247 -1.391383

14 6 0 -3.584293 0.279534 0.464307

15 6 0 -4.721319 0.315717 -0.257106

16 6 0 -6.056660 0.338430 0.295639

17 6 0 -7.169454 0.368948 -0.456960

18 6 0 -8.569305 0.391222 0.066570

19 6 0 4.094914 0.426908 0.222406

20 6 0 4.064838 1.861203 -0.124175

21 6 0 2.927108 2.544388 -0.343144

22 6 0 1.601296 1.859232 -0.180396

23 8 0 0.589778 2.424950 -0.534070

24 8 0 5.137538 -0.107420 0.563821

25 6 0 2.889943 3.985503 -0.748854

26 6 0 0.599642 -2.341904 1.732436

27 1 0 0.109293 -0.105880 -0.937580

28 1 0 3.668815 -2.865736 -1.872570

29 1 0 4.816230 -1.674047 -1.204272

30 1 0 4.307774 -3.053216 -0.246208

31 1 0 2.597456 1.316768 2.285975

32 1 0 0.844116 1.461654 2.354744

33 1 0 1.616216 -0.094726 2.655338

34 1 0 -1.312003 -0.245248 1.694507

35 1 0 -0.940404 1.335062 1.043080

36 1 0 -0.701382 -2.920633 -0.904397

37 1 0 -3.623179 0.263594 1.551120

38 1 0 -4.624151 0.324851 -1.341251

39 1 0 -6.148214 0.328150 1.381489

40 1 0 -7.059000 0.377508 -1.541121

41 1 0 -8.598700 0.380172 1.158526

42 1 0 -9.105168 1.282455 -0.280047

43 1 0 -9.139557 -0.470821 -0.298452

44 1 0 5.038632 2.337528 -0.200620

45 1 0 3.895552 4.394347 -0.854730

46 1 0 2.356375 4.100329 -1.696082

47 1 0 2.338230 4.584044 -0.018044

48 1 0 -0.160168 -1.977257 2.426367

49 1 0 0.521210 -3.430510 1.705703

50 1 0 1.585427 -2.082175 2.118745

---------------------------------------------------------------------

Standard orientation of Conf. C

---------------------------------------------------------------------

Center Atomic Atomic Coordinates (Angstroms)

Number Number Type X Y Z

---------------------------------------------------------------------

1 6 0 0.335858 -1.806001 0.311080

2 6 0 1.471260 -2.257219 -0.599100

3 6 0 2.780638 -1.558386 -0.446860

4 6 0 2.798372 -0.317907 0.095292

5 6 0 1.560093 0.473396 0.526022

6 6 0 0.238167 -0.260203 0.139358

7 6 0 3.967596 -2.314764 -0.979341

8 6 0 1.662065 0.807227 2.049127

9 6 0 -1.053240 0.281104 0.772467

10 8 0 -0.885346 -2.380170 -0.119604

11 8 0 1.297245 -3.150659 -1.408085

12 6 0 -2.251924 0.252985 -0.182280

13 8 0 -2.113106 0.270391 -1.391223

14 6 0 -3.584271 0.279690 0.464356

15 6 0 -4.721245 0.315782 -0.257143

16 6 0 -6.056616 0.338462 0.295540

17 6 0 -7.169398 0.368825 -0.457079

18 6 0 -8.569255 0.391024 0.066439

19 6 0 4.094901 0.427031 0.222055

20 6 0 4.064640 1.861363 -0.124397

21 6 0 2.926841 2.544537 -0.343025

22 6 0 1.601118 1.859239 -0.180234

23 8 0 0.589534 2.424838 -0.533937

24 8 0 5.137706 -0.107222 0.563076

25 6 0 2.889499 3.985672 -0.748624

26 6 0 0.599593 -2.342171 1.732393

27 1 0 0.109283 -0.105868 -0.937393

28 1 0 3.668729 -2.867302 -1.871173

29 1 0 4.815819 -1.673870 -1.205465

30 1 0 4.309014 -3.051887 -0.244940

31 1 0 2.597519 1.317116 2.285868

32 1 0 0.844144 1.460984 2.355261

33 1 0 1.617276 -0.095015 2.655317

34 1 0 -1.312119 -0.245787 1.694547

35 1 0 -0.940424 1.334744 1.043747

36 1 0 -0.701441 -2.920163 -0.904836

37 1 0 -3.623222 0.263823 1.551168

38 1 0 -4.624010 0.324825 -1.341282

39 1 0 -6.148187 0.328276 1.381392

40 1 0 -7.058938 0.377298 -1.541242

41 1 0 -8.598653 0.380100 1.158399

42 1 0 -9.105209 1.282167 -0.280275

43 1 0 -9.139423 -0.471117 -0.298482

44 1 0 5.038406 2.337725 -0.201006

45 1 0 3.895047 4.395010 -0.853198

46 1 0 2.357168 4.100321 -1.696585

47 1 0 2.336505 4.583878 -0.018527

48 1 0 -0.160012 -1.977290 2.426423

49 1 0 0.520817 -3.430748 1.705587

50 1 0 1.585484 -2.082748 2.118615

---------------------------------------------------------------------

Standard orientation of Conf. D

---------------------------------------------------------------------

Center Atomic Atomic Coordinates (Angstroms)

Number Number Type X Y Z

---------------------------------------------------------------------

1 6 0 -0.409557 -2.054143 0.148552

2 6 0 -1.319434 -1.814375 1.353383

3 6 0 -2.429965 -0.832918 1.223782

4 6 0 -2.337838 0.122729 0.271196

5 6 0 -1.164878 0.298516 -0.697519

6 6 0 0.029796 -0.681082 -0.427177

7 6 0 -3.535663 -0.980146 2.233774

8 6 0 -1.719234 0.194847 -2.156364

9 6 0 0.961481 -0.820932 -1.657455

10 8 0 0.765227 -2.706468 0.600680

11 8 0 -1.135419 -2.445406 2.379944

12 6 0 2.243480 -1.645736 -1.485844

13 8 0 2.370410 -2.687132 -2.110250

14 6 0 3.351507 -1.147066 -0.651766

15 6 0 3.454397 0.091462 -0.130664

16 6 0 4.589022 0.553221 0.643222

17 6 0 4.689224 1.793263 1.149323

18 6 0 5.838906 2.308124 1.955319

19 6 0 -3.406769 1.172251 0.152677

20 6 0 -2.955103 2.547959 -0.140796

21 6 0 -1.685165 2.855777 -0.457958

22 6 0 -0.662913 1.761620 -0.541425

23 8 0 0.517534 2.044415 -0.523963

24 8 0 -4.592273 0.932777 0.309405

25 6 0 -1.203370 4.252615 -0.703347

26 6 0 -1.142593 -2.997217 -0.827420

27 1 0 0.616734 -0.232500 0.377806

28 1 0 -3.118819 -1.350466 3.170712

29 1 0 -4.080576 -0.055668 2.407370

30 1 0 -4.265310 -1.720115 1.889019

31 1 0 -2.605253 0.814885 -2.293769

32 1 0 -0.975907 0.516648 -2.886405

33 1 0 -1.995954 -0.832450 -2.389198

34 1 0 0.434522 -1.289200 -2.488118

35 1 0 1.248212 0.182862 -1.980736

36 1 0 0.580873 -3.053277 1.488956

37 1 0 4.151782 -1.870006 -0.512923

38 1 0 2.657346 0.814219 -0.288781

39 1 0 5.394452 -0.160302 0.814712

40 1 0 3.874214 2.493615 0.966713

41 1 0 6.614028 1.550561 2.092207

42 1 0 6.297812 3.180980 1.476401

43 1 0 5.509250 2.639570 2.946939

44 1 0 -3.726224 3.310300 -0.071850

45 1 0 -2.015089 4.974760 -0.608505

46 1 0 -0.411584 4.512799 0.004065

47 1 0 -0.762355 4.345301 -1.700331

48 1 0 -0.490495 -3.229424 -1.669762

49 1 0 -1.363972 -3.935771 -0.314101

50 1 0 -2.083511 -2.585406 -1.197915

---------------------------------------------------------------------

Standard orientation of Conf. E

---------------------------------------------------------------------

Center Atomic Atomic Coordinates (Angstroms)

Number Number Type X Y Z

---------------------------------------------------------------------

1 6 0 0.554109 -1.926989 0.338361

2 6 0 1.735703 -2.249361 -0.587327

3 6 0 2.953999 -1.378984 -0.437817

4 6 0 2.822783 -0.136176 0.080312

5 6 0 1.494730 0.499162 0.504565

6 6 0 0.274136 -0.401592 0.145538

7 6 0 4.228942 -1.994478 -0.945673

8 6 0 1.560098 0.863580 2.022154

9 6 0 -1.062180 0.017729 0.778302

10 8 0 -0.589821 -2.679767 -0.006842

11 8 0 1.699847 -3.151288 -1.395245

12 6 0 -2.273440 -0.189821 -0.130920

13 8 0 -2.174448 -0.643821 -1.261498

14 6 0 -3.570890 0.211352 0.448381

15 6 0 -4.725249 0.086223 -0.236577

16 6 0 -6.031446 0.446170 0.263870

17 6 0 -7.160938 0.302336 -0.450437

18 6 0 -8.534369 0.654609 0.020483

19 6 0 4.012145 0.769409 0.194282

20 6 0 3.792772 2.193740 -0.135093

21 6 0 2.577548 2.723727 -0.360178

22 6 0 1.359566 1.857339 -0.238403

23 8 0 0.296175 2.251507 -0.669665

24 8 0 5.123361 0.377787 0.513222

25 6 0 2.352614 4.150627 -0.757337

26 6 0 0.899884 -2.404664 1.756862

27 1 0 0.132225 -0.281295 -0.934542

28 1 0 4.002944 -2.618624 -1.810997

29 1 0 4.987802 -1.259075 -1.201125

30 1 0 4.662876 -2.648877 -0.182035

31 1 0 2.435537 1.472984 2.250971

32 1 0 0.678369 1.429114 2.327884

33 1 0 1.613972 -0.033355 2.635585

34 1 0 -1.258462 -0.512379 1.716756

35 1 0 -1.063060 1.080908 1.030823

36 1 0 -0.919806 -2.403858 -0.874549

37 1 0 -3.570781 0.609742 1.460406

38 1 0 -4.666923 -0.319177 -1.245032

39 1 0 -6.086783 0.850574 1.274097

40 1 0 -7.085686 -0.104347 -1.458702

41 1 0 -8.530116 1.053864 1.037133

42 1 0 -8.995804 1.399835 -0.637609

43 1 0 -9.192577 -0.221611 0.000469

44 1 0 4.696118 2.794220 -0.200364

45 1 0 3.294492 4.695714 -0.830271

46 1 0 1.836490 4.200952 -1.719751

47 1 0 1.703381 4.660132 -0.039038

48 1 0 0.142946 -2.081641 2.474510

49 1 0 0.900148 -3.496204 1.749638

50 1 0 1.878697 -2.066575 2.095871

---------------------------------------------------------------------

Standard orientation of Conf. F

---------------------------------------------------------------------

Center Atomic Atomic Coordinates (Angstroms)

Number Number Type X Y Z

---------------------------------------------------------------------

1 6 0 -0.126029 -1.761940 -0.028234

2 6 0 -1.459294 -2.309335 0.465257

3 6 0 -2.692371 -1.618287 -0.010830

4 6 0 -2.599786 -0.332730 -0.425231

5 6 0 -1.323289 0.512904 -0.396767

6 6 0 -0.142878 -0.236996 0.297509

7 6 0 -3.951296 -2.438393 0.073199

8 6 0 -0.984190 0.991862 -1.845208

9 6 0 1.253607 0.380530 0.107413

10 8 0 0.936814 -2.361618 0.690586

11 8 0 -1.494525 -3.268494 1.214564

12 6 0 2.144652 0.214698 1.345519

13 8 0 1.661849 0.148457 2.462350

14 6 0 3.613648 0.220774 1.193509

15 6 0 4.311031 0.341731 0.046856

16 6 0 5.757627 0.359580 -0.027791

17 6 0 6.444752 0.486774 -1.174549

18 6 0 7.935072 0.507013 -1.293513

19 6 0 -3.830304 0.401955 -0.870400

20 6 0 -3.962425 1.798852 -0.413657

21 6 0 -2.970414 2.474258 0.193703

22 6 0 -1.630435 1.824146 0.383066

23 8 0 -0.796272 2.365832 1.075008

24 8 0 -4.702587 -0.114434 -1.549837

25 6 0 -3.114748 3.873286 0.708398

26 6 0 0.057445 -2.165237 -1.504717

27 1 0 -0.336042 -0.179875 1.373586

28 1 0 -3.904431 -3.076741 0.956483

29 1 0 -4.854000 -1.833381 0.099018

30 1 0 -4.032547 -3.098416 -0.796816

31 1 0 -1.833598 1.494353 -2.310997

32 1 0 -0.152005 1.697185 -1.831937

33 1 0 -0.706766 0.155827 -2.483404

34 1 0 1.746947 -0.019452 -0.779539

35 1 0 1.173436 1.460803 -0.037380

36 1 0 0.551893 -2.964522 1.346702

37 1 0 4.141756 0.128615 2.139830

38 1 0 3.786098 0.438174 -0.900918

39 1 0 6.300283 0.261520 0.911559

40 1 0 5.886211 0.583780 -2.105892

41 1 0 8.423959 0.404343 -0.322177

42 1 0 8.289842 -0.303856 -1.940129

43 1 0 8.281638 1.440370 -1.752053

44 1 0 -4.932716 2.253275 -0.594907

45 1 0 -4.120174 4.258319 0.534103

46 1 0 -2.899558 3.909023 1.779678

47 1 0 -2.391080 4.541971 0.233138

48 1 0 0.976685 -1.736277 -1.908483

49 1 0 0.158057 -3.251377 -1.550953

50 1 0 -0.778361 -1.874289 -2.141073

---------------------------------------------------------------------

**Table S2**. Cartesian coordinates of the low-energy conformers (≥5%) of (8*S*,9*R*,10*R*)-**1**

Standard orientation of Conf. A

---------------------------------------------------------------------

Center Atomic Atomic Coordinates (Angstroms)

Number Number Type X Y Z

---------------------------------------------------------------------

1 6 0 0.148453 1.968089 0.762708

2 6 0 1.027131 2.427762 -0.399440

3 6 0 2.266067 1.660584 -0.683224

4 6 0 2.345531 0.382276 -0.246922

5 6 0 1.226446 -0.385217 0.466069

6 6 0 -0.091380 0.440473 0.634298

7 6 0 3.306637 2.394675 -1.486273

8 6 0 1.765765 -0.939801 1.821453

9 6 0 -1.033089 -0.028350 1.775156

10 8 0 -1.120583 2.603299 0.646289

11 8 0 0.713526 3.422529 -1.032133

12 6 0 -1.728063 -1.372421 1.564120

13 8 0 -1.271898 -2.385514 2.080382

14 6 0 -2.967037 -1.441774 0.774564

15 6 0 -3.601694 -0.395914 0.205708

16 6 0 -4.816951 -0.510653 -0.572284

17 6 0 -5.435368 0.537832 -1.140815

18 6 0 -6.686540 0.466323 -1.955640

19 6 0 3.568085 -0.445635 -0.546621

20 6 0 3.340047 -1.855991 -0.918759

21 6 0 2.125711 -2.432653 -0.883844

22 6 0 0.945806 -1.606927 -0.457399

23 8 0 -0.164827 -1.896772 -0.839671

24 8 0 4.696778 0.014637 -0.504655

25 6 0 1.849052 -3.842227 -1.302417

26 6 0 0.806987 2.439995 2.074841

27 1 0 -0.643885 0.332731 -0.302146

28 1 0 2.810497 3.065290 -2.188734

29 1 0 3.926643 3.014322 -0.830370

30 1 0 3.978242 1.728030 -2.021351

31 1 0 2.736890 -1.420036 1.704123

32 1 0 1.887013 -0.129901 2.541025

33 1 0 1.074833 -1.670813 2.242095

34 1 0 -0.492552 -0.104739 2.717963

35 1 0 -1.785716 0.750584 1.902071

36 1 0 -1.007697 3.340097 0.022519

37 1 0 -3.362240 -2.448764 0.664149

38 1 0 -3.196812 0.608002 0.314261

39 1 0 -5.234556 -1.509253 -0.695071

40 1 0 -5.001340 1.528743 -1.007962

41 1 0 -7.059421 -0.556546 -2.044023

42 1 0 -7.479406 1.081545 -1.514708

43 1 0 -6.520848 0.857213 -2.966245

44 1 0 4.223587 -2.399730 -1.241586

45 1 0 2.755733 -4.343122 -1.643814

46 1 0 1.417841 -4.413900 -0.474603

47 1 0 1.105071 -3.859267 -2.103045

48 1 0 0.886300 3.529530 2.064527

49 1 0 0.181392 2.163367 2.924610

50 1 0 1.806882 2.028531 2.222947

---------------------------------------------------------------------

Standard orientation of Conf. B

---------------------------------------------------------------------

Center Atomic Atomic Coordinates (Angstroms)

Number Number Type X Y Z

---------------------------------------------------------------------

1 6 0 -1.352960 -2.205272 0.172594

2 6 0 -2.373167 -1.885892 -0.919004

3 6 0 -3.082695 -0.582741 -0.849938

4 6 0 -2.505284 0.427776 -0.160464

5 6 0 -1.128367 0.370302 0.511826

6 6 0 -0.390014 -0.993948 0.314756

7 6 0 -4.376197 -0.515829 -1.616427

8 6 0 -1.271892 0.761468 2.015397

9 6 0 0.699061 -1.322523 1.370695

10 8 0 -0.573495 -3.317528 -0.245888

11 8 0 -2.604129 -2.710368 -1.787427

12 6 0 1.939897 -0.432701 1.359866

13 8 0 2.060604 0.480633 2.165623

14 6 0 2.988775 -0.774273 0.378709

15 6 0 4.166340 -0.120802 0.337548

16 6 0 5.240344 -0.409569 -0.584708

17 6 0 6.405897 0.259445 -0.599318

18 6 0 7.542043 -0.002197 -1.534647

19 6 0 -3.164692 1.780665 -0.098899

20 6 0 -2.278822 2.955463 -0.223994

21 6 0 -0.938201 2.858305 -0.261554

22 6 0 -0.310359 1.495974 -0.185597

23 8 0 0.784038 1.307155 -0.666468

24 8 0 -4.369525 1.918741 0.030815

25 6 0 -0.013548 4.021664 -0.436720

26 6 0 -2.124389 -2.609444 1.444898

27 1 0 0.112098 -0.938909 -0.654860

28 1 0 -4.281959 -1.100514 -2.532609

29 1 0 -5.186824 -0.964261 -1.032600

30 1 0 -4.677831 0.500924 -1.853349

31 1 0 -1.872353 1.661753 2.143629

32 1 0 -1.761095 -0.038858 2.570615

33 1 0 -0.293463 0.938181 2.462157

34 1 0 0.287485 -1.288193 2.379265

35 1 0 1.013588 -2.350635 1.175621

36 1 0 -1.034589 -3.718972 -1.001281

37 1 0 2.784084 -1.586831 -0.313826

38 1 0 4.319413 0.684624 1.053627

39 1 0 5.079116 -1.215063 -1.300463

40 1 0 6.549886 1.062164 0.123759

41 1 0 7.322413 -0.818242 -2.226689

42 1 0 7.780051 0.890527 -2.124635

43 1 0 8.454512 -0.258345 -0.983774

44 1 0 -2.786210 3.913326 -0.298787

45 1 0 -0.563914 4.958157 -0.535056

46 1 0 0.672879 4.101440 0.412101

47 1 0 0.612052 3.877474 -1.321398

48 1 0 -2.740276 -3.484827 1.226134

49 1 0 -1.422162 -2.890046 2.230936

50 1 0 -2.777467 -1.819157 1.818142

---------------------------------------------------------------------

Standard orientation of Conf. C

---------------------------------------------------------------------

Center Atomic Atomic Coordinates (Angstroms)

Number Number Type X Y Z

---------------------------------------------------------------------

1 6 0 -0.193904 -1.829308 0.312255

2 6 0 -1.050010 -2.102031 -0.923199

3 6 0 -2.372413 -1.426977 -1.028290

4 6 0 -2.578616 -0.293558 -0.318615

5 6 0 -1.540610 0.386793 0.578582

6 6 0 -0.133059 -0.293288 0.528734

7 6 0 -3.342134 -2.067885 -1.984102

8 6 0 -2.116908 0.479256 2.028105

9 6 0 0.766528 0.097635 1.723640

10 8 0 1.129800 -2.263195 0.052468

11 8 0 -0.649202 -2.874312 -1.776736

12 6 0 2.257817 -0.227772 1.608362

13 8 0 2.805298 -0.892757 2.470952

14 6 0 2.998031 0.396410 0.488947

15 6 0 4.316300 0.189657 0.308998

16 6 0 5.108677 0.775907 -0.748974

17 6 0 6.422633 0.545079 -0.908824

18 6 0 7.278869 1.127053 -1.987563

19 6 0 -3.879245 0.449730 -0.440630

20 6 0 -3.800391 1.924822 -0.464661

21 6 0 -2.664674 2.607526 -0.235143

22 6 0 -1.406254 1.853293 0.077747

23 8 0 -0.331358 2.404212 -0.033343

24 8 0 -4.957152 -0.112724 -0.537764

25 6 0 -2.558813 4.099454 -0.310859

26 6 0 -0.753471 -2.666737 1.480252

27 1 0 0.349462 0.084146 -0.377055

28 1 0 -4.096371 -1.377636 -2.353279

29 1 0 -2.790228 -2.500444 -2.819256

30 1 0 -3.874092 -2.887760 -1.490300

31 1 0 -2.149990 -0.505400 2.491504

32 1 0 -1.501715 1.124669 2.656012

33 1 0 -3.129892 0.882093 2.033273

34 1 0 0.707562 1.183499 1.848953

35 1 0 0.421438 -0.357539 2.651929

36 1 0 1.103255 -2.820828 -0.742353

37 1 0 2.446416 1.047356 -0.184276

38 1 0 4.821765 -0.470923 1.011039

39 1 0 4.596026 1.438036 -1.446445

40 1 0 6.919947 -0.119871 -0.202935

41 1 0 6.710181 1.780608 -2.653115

42 1 0 8.105992 1.708709 -1.564135

43 1 0 7.737655 0.338433 -2.595277

44 1 0 -4.733068 2.431686 -0.696647

45 1 0 -3.514367 4.552752 -0.576972

46 1 0 -2.224604 4.516425 0.644155

47 1 0 -1.807021 4.391262 -1.048657

48 1 0 -0.143910 -2.513339 2.371318

49 1 0 -1.795877 -2.441328 1.712657

50 1 0 -0.687764 -3.724746 1.216679

---------------------------------------------------------------------

**Table S3**. Cartesian coordinates of the low-energy conformers (≥5%) of(4*S*,5*R*,6*R*)-**3**

Standard orientation of Conf. A

---------------------------------------------------------------------

Center Atomic Atomic Coordinates (Angstroms)

Number Number Type X Y Z

---------------------------------------------------------------------

1 6 0 1.639762 -2.022915 -0.403018

2 6 0 2.082582 -1.261417 -1.669026

3 6 0 2.372210 0.188514 -1.489296

4 6 0 2.139582 0.771769 -0.288591

5 8 0 2.174483 -1.820988 -2.744458

6 6 0 2.903039 0.920508 -2.697704

7 6 0 2.448579 2.230093 -0.075871

8 6 0 1.253870 3.176275 -0.252713

9 8 0 0.202228 2.808919 -0.728205

10 6 0 1.477997 4.603987 0.197251

11 6 0 0.634683 -1.149844 0.368497

12 6 0 1.606868 -0.060444 0.897186

13 6 0 2.818473 -0.941905 1.275508

14 8 0 3.671900 -0.720555 2.087365

15 8 0 2.807682 -2.049687 0.492901

16 6 0 1.105326 0.762369 2.095434

17 6 0 1.224264 -3.450502 -0.670983

18 6 0 -0.587951 -0.667719 -0.423507

19 6 0 -1.921592 -0.849469 0.300928

20 6 0 -3.093912 -0.267136 -0.384059

21 6 0 -4.340515 -0.394670 0.113702

22 6 0 -5.536375 0.144185 -0.491040

23 6 0 -6.762575 -0.008310 0.038215

24 6 0 -8.030031 0.526744 -0.544406

25 8 0 -2.009011 -1.446973 1.362321

26 6 0 0.583457 -0.057141 3.281160

27 1 0 0.276469 -1.744025 1.213054

28 1 0 2.299753 1.798581 -2.941842

29 1 0 3.935174 1.255082 -2.546877

30 1 0 2.897691 0.257963 -3.561944

31 1 0 2.887886 2.407416 0.909894

32 1 0 3.209674 2.566626 -0.787840

33 1 0 1.464774 4.643904 1.292165

34 1 0 2.454504 4.979009 -0.121703

35 1 0 0.686828 5.247521 -0.185617

36 1 0 1.930527 1.382474 2.454890

37 1 0 0.320233 1.443840 1.752336

38 1 0 2.017874 -3.994670 -1.182979

39 1 0 0.994621 -3.951237 0.271378

40 1 0 0.338719 -3.480818 -1.308945

41 1 0 -0.493047 0.382050 -0.717712

42 1 0 -0.684866 -1.210648 -1.373710

43 1 0 -2.920342 0.275810 -1.309882

44 1 0 -4.453258 -0.947110 1.044902

45 1 0 -5.421115 0.699570 -1.421114

46 1 0 -6.856810 -0.566804 0.969309

47 1 0 -7.855831 1.073578 -1.473679

48 1 0 -8.738773 -0.283134 -0.752302

49 1 0 -8.532134 1.200158 0.159712

50 1 0 0.333399 0.614872 4.106611

51 1 0 -0.317124 -0.620551 3.035188

52 1 0 1.340465 -0.753005 3.651302

---------------------------------------------------------------------

Standard orientation of Conf. B

---------------------------------------------------------------------

Center Atomic Atomic Coordinates (Angstroms)

Number Number Type X Y Z

---------------------------------------------------------------------

1 6 0 -2.327552 -1.248402 0.871990

2 6 0 -2.018248 -0.211891 1.970361

3 6 0 -1.527561 1.112749 1.496775

4 6 0 -1.275887 1.298262 0.178667

5 8 0 -2.159012 -0.475995 3.148491

6 6 0 -1.326486 2.170177 2.554368

7 6 0 -0.798428 2.634878 -0.324963

8 6 0 0.724580 2.779882 -0.431820

9 8 0 1.487804 1.992488 0.084097

10 6 0 1.214291 3.982442 -1.208467

11 6 0 -1.201663 -1.206654 -0.177026

12 6 0 -1.526832 0.162030 -0.836108

13 6 0 -3.068543 0.076192 -0.883201

14 8 0 -3.819159 0.629387 -1.636117

15 8 0 -3.486411 -0.727866 0.125706

16 6 0 -0.922865 0.391332 -2.231910

17 6 0 -2.695129 -2.610632 1.411728

18 6 0 0.227788 -1.396492 0.349566

19 6 0 1.112272 -2.244617 -0.570189

20 6 0 2.575404 -2.104573 -0.505033

21 6 0 3.263045 -1.118909 0.110739

22 6 0 4.706907 -1.023277 0.114488

23 6 0 5.377513 -0.020114 0.705675

24 6 0 6.864630 0.122257 0.741074

25 8 0 0.615849 -3.074811 -1.318830

26 6 0 -1.179087 -0.727273 -3.248666

27 1 0 -1.406586 -2.006067 -0.893418

28 1 0 -0.320828 2.596176 2.520815

29 1 0 -2.042937 2.991712 2.444746

30 1 0 -1.476276 1.736786 3.542149

31 1 0 -1.241950 2.882775 -1.292607

32 1 0 -1.127070 3.434443 0.348393

33 1 0 0.648695 4.882984 -0.953228

34 1 0 2.276155 4.143280 -1.026139

35 1 0 1.063607 3.806430 -2.279329

36 1 0 -1.340340 1.314857 -2.640908

37 1 0 0.156206 0.549589 -2.128848

38 1 0 -3.531457 -2.537241 2.106942

39 1 0 -2.968676 -3.273073 0.588537

40 1 0 -1.855156 -3.051259 1.952120

41 1 0 0.696422 -0.438838 0.572352

42 1 0 0.210649 -1.940999 1.303144

43 1 0 3.108912 -2.877478 -1.053800

44 1 0 2.733239 -0.321112 0.623894

45 1 0 5.260076 -1.813339 -0.391879

46 1 0 4.803517 0.762461 1.201180

47 1 0 7.367585 -0.694040 0.217902

48 1 0 7.179736 1.067019 0.283274

49 1 0 7.232436 0.144069 1.773393

50 1 0 -0.778197 -0.434141 -4.222624

51 1 0 -0.700719 -1.666685 -2.969456

52 1 0 -2.248509 -0.908144 -3.381674

---------------------------------------------------------------------

**Table S4**. The CD data of 13-hydroxy-trichodermolide (**4**), and the reported trichodermolide, dihydro-trichodermolide, and 13-hydroxy-dihydro-trichodermolide

| Compound (Solvent) | Structure | CD λ_max_nm (Δε) |
| --- | --- | --- |
| 13-Hydroxy-trichodermolide (**4**) (MeOH) |  | 222 nm (˗9.9)  268 nm (+7.8) |
| Trichodermolide (MeOH) |  | 223 nm (˗55.2)  267nm (+36.2) |
| Dihydrotrichodermolide (EtOH) |  | 224 nm (˗15.4)  260 nm (+5.4) |
| 13-Hydroxy-dihydrotrichodermolide (MeOH) |  | 222 nm (˗48.1)  268 nm (+39.0) |
